# Thiophen-2-ylsulfonyl indole-3-carbaldehyde thiosemicarbazones as selective prostate cancer agents: biological profiling and molecular targeting studies

**DOI:** 10.1039/d6ra08602k

**Published:** 2026-11-09

**Authors:** Kazma Batool, Hande İpek İnce, Ayşe Merve Şenol, Fatma Ateş, Metab Alharbi, Abdullah F. Alasmari, Asif Rasool, Halil Şenol, Zahid Shafiq, Abdulrahman Alshammari

**Affiliations:** a Institute of Chemical Sciences, Bahauddin Zakariya University Multan 60800 Pakistan zahidshafiq@bzu.edu.pk; b Department of Biophysics, Faculty of Medicine, Bezmialem Vakif University Fatih Istanbul 34093 Türkiye; c Department of Medical Services and Techniques, Program of Medical Laboratory Techniques, University of Health Sciences Istanbul 34668 Türkiye; d Department of Pharmacology and Toxicology, College of Pharmacy, King Saud University Post Box 2455 Riyadh 11451 Saudi Arabia; e School of Chemistry and Chemical Engineering, Nanjing University Nanjing 210093 China; f Department of Pharmaceutical Chemistry, Faculty of Pharmacy, Bezmialem Vakif University Fatih Istanbul 34093 Türkiye hsenol@bezmialem.edu.tr

## Abstract

A series of thiophen-2-ylsulfonyl indole-3-carbaldehyde thiosemicarbazone derivatives 5a–5t was synthesized and evaluated for anticancer activity using biological and computational methods. The cytotoxic activity of these compounds was tested on A549, LNCaP, and HUVEC cell lines utilizing the MTT assay, with sorafenib used as a control drug. The activity of the derivatives was shown to be cell line- and substituent-dependent, with the highest cytotoxicity against LNCaP cells. Compound 5d, bearing a 2,3-dichlorophenyl group, was the most potent derivative against LNCaP cells (IC_50_ = 2.50 µM; SI = 33.7), while compounds 5b and 5c also showed notable activity (IC_50_ = 7.96 and 5.30 µM, respectively). These synthesized compounds showed preferential activity towards prostate cancer (LNCaP) cells specifically in comparison with lung cancer A549 cells. Thus, subsequent computational studies were directed to the molecular targets associated with prostate cancer. The docking study of compounds 5b–5d against AR, EGFR, VEGFR-2, AKT1, mTOR, and PI3Kα, followed by MM-GBSA analysis, indicated their good binding activity with particular reference to the AR target. An MD simulation of 250 ns on the AR-compound 5d complex confirmed the stability and constant interaction with important residues. ADME and DFT analyses determined the properties of the selected compounds. Overall, compound 5d emerged as a promising lead for further investigation against prostate cancer. Our data provide a foundation for the rational design and optimization of thiosemicarbazone-based anticancer agents and advance research into their molecular mechanisms, target selectivity, and preclinical therapeutic potential.

## Abstract 2

A series of thiophen-2-ylsulfonyl indole-3-carbaldehyde thiosemicarbazone derivatives 5a–5t was synthesized and evaluated for anticancer activity using biological and computational methods.

## Graphical abstract

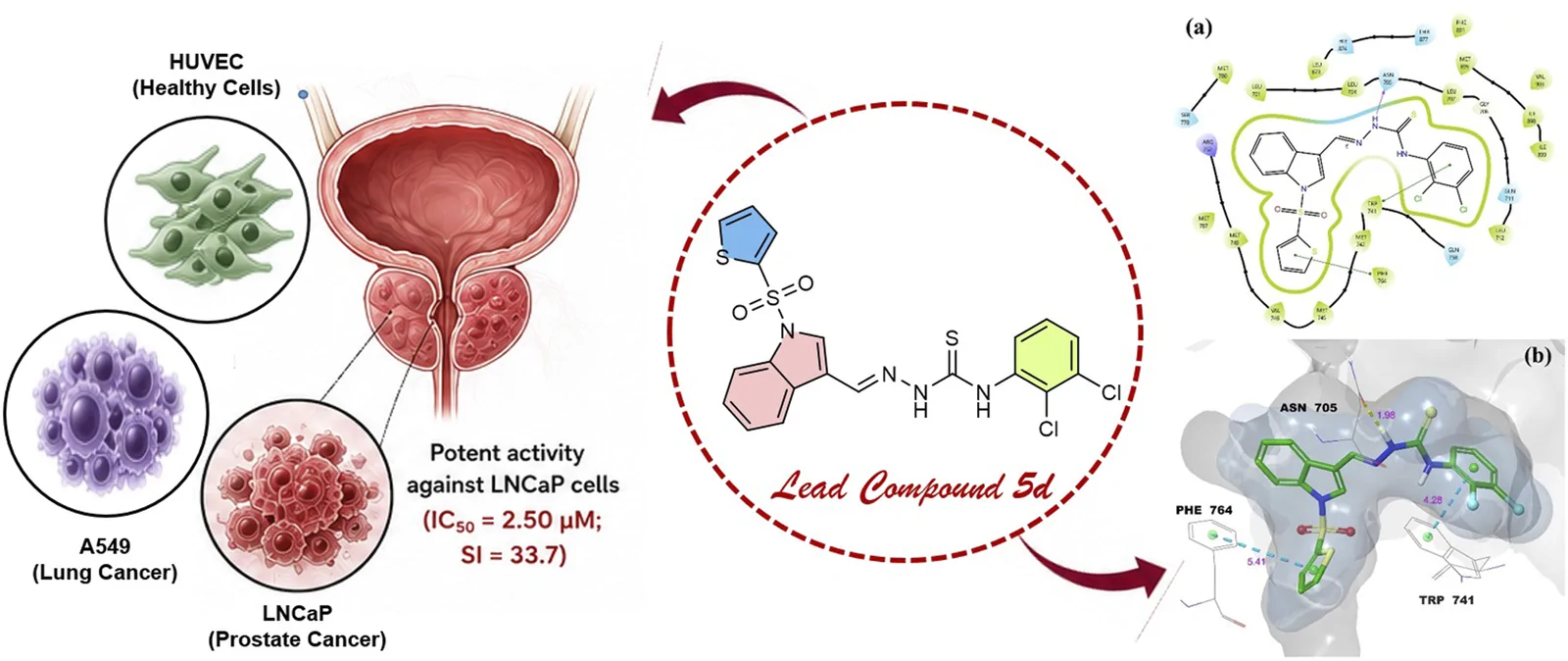

## 1 Introduction

Cancer is a significant contributor to mortality and morbidity worldwide. Based on GLOBOCAN 2024 estimates, there were about 20.6 million cancer cases and 9.7–9.8 million cancer-related deaths in 2024 worldwide, reflecting the continuing and increasing global burden of malignant diseases.^1–8^

Lung cancer constitutes a major disease burden. About 2.6 million people were diagnosed with lung cancer in 2024, making it the most commonly diagnosed type of cancer and the leading cause of cancer-related deaths. Smoking is the main preventable risk factor for cancer, although cancer can also be caused by other environmental factors.^9,10^ Prostate cancer is a common type of cancer in men, with about 1.5 million new cases and 420 000 deaths occurring worldwide in 2024. This type of cancer is the second most common in terms of diagnosis and is the fourth most common for cancer-related deaths among men worldwide.^11–13^

Lung cancer and prostate cancer are characterized by molecular signaling pathways that control cell growth, survival, angiogenesis, metabolism, and cancer progression. The main molecular targets in the treatment of lung cancer are EGFR, VEGFR2, and the PI3K/AKT/mTOR pathway, which govern cell growth, survival, angiogenesis, metabolic adaptation, and resistance to apoptosis.^14–23^ Prostate cancer progression is due to the androgen receptor (AR)-mediated transcriptional program for proliferation, differentiation, and survival of tumor cells. In addition, aberrant stimulation of EGFR and PI3K/AKT/mTOR signaling pathways leads to enhanced cell growth and metabolism and also confers resistance to apoptosis, whereas stimulation of the VEGFR2-mediated signaling pathway drives tumor angiogenesis. Notably, extensive interaction between these pathways helps maintain oncogenic signaling and may also cause drug resistance. Thus, AR, EGFR, VEGFR2, PI3K/P110α, AKT1, and mTOR represent complementary targets for developing anticancer drugs in prostate cancer.^24–28^

Indole is one of the privileged heterocyclic cores in the field of medicinal chemistry. It is extensively investigated for the development of anticancer drugs because of its diversity and capability to address multiple cancer pathways. *N*-Sulfonyl derivatives have additional potential for tuning their recognition, potency, and physicochemical properties.^29,30^ Thiosemicarbazones are versatile bioactive compounds containing the hydrazinecarbothioamide pharmacophore, with multiple heteroatom donor/acceptor sites and metal-chelating ability. They exhibit diverse biological activities, particularly anticancer effects mediated through metal-dependent processes, ribonucleotide reductase inhibition, oxidative stress, and apoptosis induction.^31–38^ Pharmacophore hybridization of indole and thiosemicarbazone moieties provides a rational strategy for developing structurally diverse anticancer candidates with improved biological profiles. Sulfonyl-indole thiosemicarbazones have attracted attention due to their promising biological activities and favorable interactions with relevant molecular targets.^39–41^

LNCaP is a well-established androgen-responsive prostate cancer model expressing functional androgen receptors (ARs). Therefore, preferential activity against LNCaP cells may indicate potential relevance for targeting AR-associated prostate cancer phenotypes.^42,43^ A549 cells and LNCaP cells have served as established models for identifying structurally diverse compounds (I–X) with potent antiproliferative activity against lung and prostate cancers, which are represented in Fig. 1.^44–53^

**Fig. 1:**
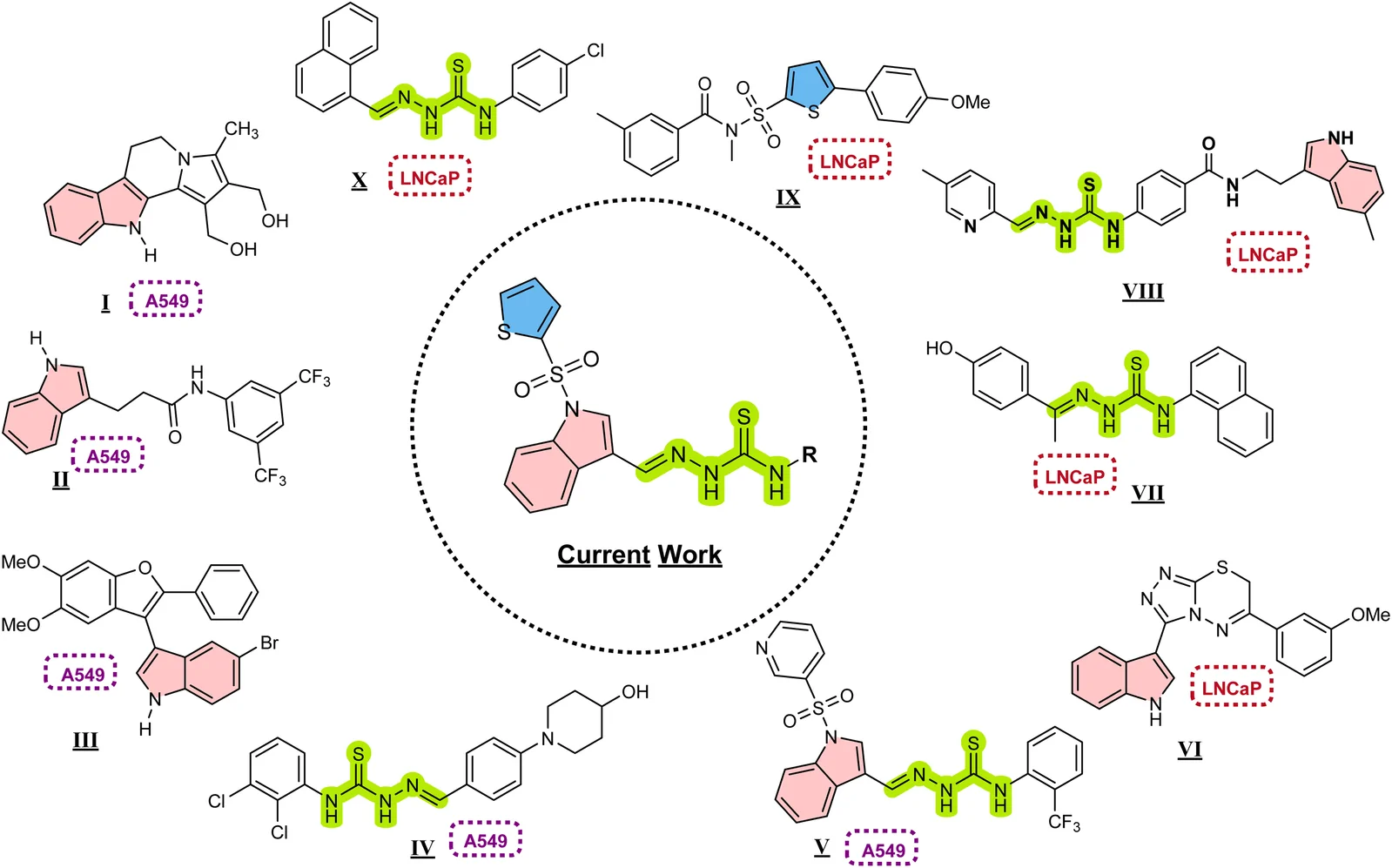
Reported anticancer compounds active against A549 and LNCaP cancer cell lines.

In the present study, a new series of thiophen-2-ylsulfonyl indole-3-carbaldehyde thiosemicarbazones was synthesized and evaluated for anticancer activity against cancer cells (LNCaP and A549), with HUVEC cells serving as a non-cancerous reference. The most promising derivatives were further investigated through molecular docking against AR, EGFR, VEGFR2, AKT1, mTOR, and PI3K/P110α, followed by MM-GBSA analysis and 250 ns molecular dynamics (MD) simulations. Complementary ADME and DFT analyses provided insights into their pharmacokinetic and electronic properties, supporting the assessment of structure–activity relationships.

Collectively, we identified compound 5d as a promising candidate for prostate cancer drug discovery, demonstrating potent and selective cytotoxicity against LNCaP cells (half-maximal inhibitory concentration (IC_50_) = 2.50 ± 0.28 µM; selectivity index (SI) = 33.7). The computational results further supported its potential interaction with the AR, offering a molecular basis for future structure-guided optimization. This work expands the exploration of thiosemicarbazone-based anticancer chemotypes and provides a foundation for investigating their therapeutic potential in prostate cancer.

## 2 Results and discussion

### 2.1 Synthesis and characterization

The synthesis of the target thiophen-2-ylsulfonyl indole-3-carbaldehyde thiosemicarbazone derivatives 5a–5t was accomplished through a two-step synthetic sequence, as illustrated in Scheme 1. The synthetic route provides a practical approach for the laboratory-scale preparation of the target compounds. The products were purified using the procedures described in the Experimental Section, and their structures were characterized using the reported spectroscopic methods. However, the synthesis was not systematically evaluated on a larger scale. Further studies are, therefore, required to assess scalability, characterize potential impurities and byproducts, and optimize purification procedures for larger-scale production.

**Scheme 1:**
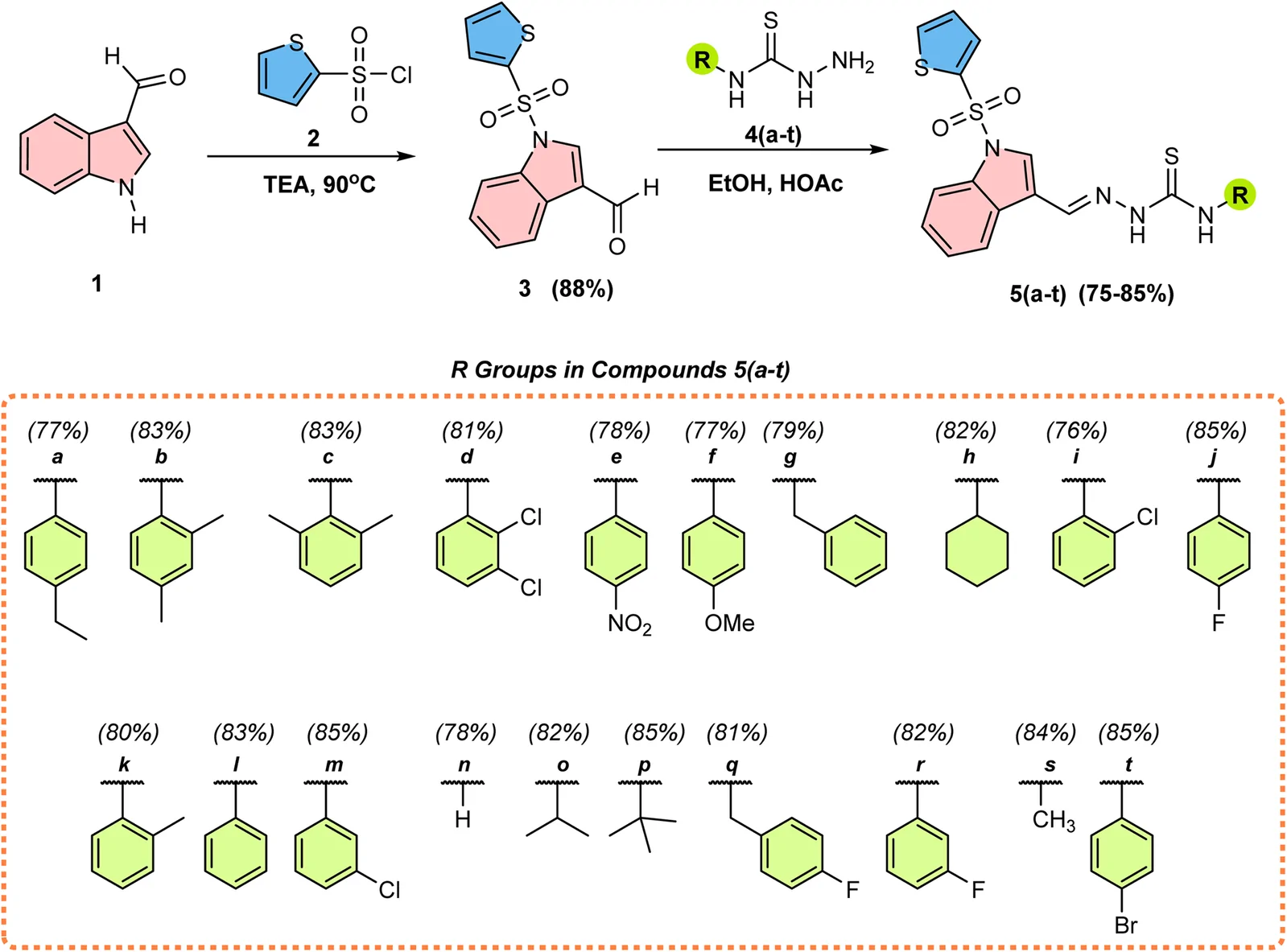
Synthesis of target compounds.

Initially, indole-3-carbaldehyde (1) was treated with thiophene-2-sulfonyl chloride (2) in TEA, affording 1-(thiophen-2-ylsulfonyl)-1*H*-indole-3-carbaldehyde (3) through selective *N*-sulfonylation of the indole nitrogen while preserving the C-3 aldehyde functionality. In the second step, intermediate 3 was subjected to condensation with a series of appropriately substituted thiosemicarbazides in EtOH under acidic conditions (HOAc), resulting in the formation of the corresponding thiosemicarbazone derivatives 5a–5t.

Based on ^1^H NMR spectroscopy, the synthesis of the thiosemicarbazone framework was evidenced by the appearance of distinctive singlets that corresponded to the protons of the thioamide group (S=C–NH) and the hydrazinic group (NH–N=C) at *δ* 11.5–12.2 and 8.4–10.5 ppm, respectively, and the singlet of the azomethine proton (HC=N) at *δ* 8.1–8.6 ppm. The protons of the aromatic and indole frameworks exhibited signal peaks in their respective spectral regions. For ^13^C NMR, the thiocarbonyl carbon (C=S) and azomethine carbon (C=N) appeared at *δ* 175–178 and 135–173 ppm, respectively.

The ^13^C NMR spectrum of compound 5q showed characteristic carbon–fluorine coupling patterns, confirming the presence of the fluorine-substituted aromatic ring. The signal at *δ* 161.23 ppm appeared as a doublet with a coupling constant of ^1^*J* = 242.0 Hz, corresponding to the directly bonded carbon–fluorine pair. Additional doublets were observed at *δ* 115.00 (^2^*J* = 21.3 Hz), 129.13 (^3^*J* = 8.1 Hz), and 135.70 ppm (^4^*J* = 2.9 Hz), attributable to long-range C–F coupling over two, three, and four bonds, respectively. HPLC of compound 5d was performed to confirm its purity. The corresponding HPLC chromatogram and purity data have been included in the SI. HPLC revealed two peaks with retention times of 4.72 and 5.21 min, accounting for 97.96% and 2.04% of the total peak area, respectively.

### 2.2 Biological activity

The cytotoxic activity of the synthesized compounds was tested on cancer cells (A549 and LNCaP), with the use of HUVEC cells as a non-cancer cell line control.^54^ LNCaP (prostate cancer) and A549 (lung cancer) cell lines were selected to evaluate the anticancer potential of the compounds against two major cancer types in men. The IC_50_ values and SI of the synthesized products are presented in Table 1.

**Table 1:** Cytotoxic activity of the synthesized indole derivatives against A549, HUVEC and LNCaP cells in terms of IC_50_ (µM) and the selectivity index (SI)^a^

Compounds	IC_50_ [µM]			Selectivity index	
	HUVEC	A549	LNCaP	A549	LNCaP
5a	64.56 ± 6.46	82.96 ± 8.30	35.88 ± 4.66	0.7	1.8
5b	**131.30 ± 11.82**	91.24 ± 10.04	**7.96 ± 0.72**	1.4	**16.5**
5c	**73.53 ± 8.09**	145.70 ± 13.11	**5.30 ± 0.53**	0.5	**13.8**
5d	**84.25 ± 10.11**	75.20 ± 8.27	**2.50 ± 0.28**	1.1	**33.7**
5e	24.66 ± 2.47	54.41 ± 5.99	12.92 ± 1.42	0.4	1.9
5f	63.11 ± 7.57	45.45 ± 4.55	70.20 ± 7.72	1.4	0.9
5g	35.76 ± 3.22	75.50 ± 9.06	60.31 ± 7.24	0.4	0.6
5h	40.54 ± 5.27	69.64 ± 7.66	53.52 ± 4.28	0.5	0.7
5i	63.42 ± 7.61	32.19 ± 3.54	15.33 ± 1.53	1.9	4.1
5j	58.73 ± 7.05	26.78 ± 2.41	39.00 ± 4.29	2.2	1.5
5k	23.96 ± 1.92	27.04 ± 3.25	29.67 ± 3.56	0.9	0.8
5l	40.64 ± 5.28	30.43 ± 3.04	8.51 ± 0.68	1.3	4.7
5m	37.24 ± 4.84	43.19 ± 5.18	24.85 ± 2.24	0.8	1.5
5n	20.04 ± 2.81	42.55 ± 3.40	24.42 ± 2.69	0.4	0.8
5o	37.89 ± 3.79	24.91 ± 2.74	41.80 ± 5.02	1.5	0.9
5p	**62.94 ± 8.18**	**9.45 ± 0.76**	21.02 ± 1.68	**6.6**	3.0
5q	41.16 ± 4.12	19.74 ± 2.57	23.01 ± 2.53	2.1	1.8
5r	27.52 ± 3.30	27.52 ± 2.48	31.88 ± 3.83	1.0	0.8
5s	99.97 ± 8.00	93.43 ± 10.28	25.81 ± 3.10	1.0	3.8
5t	82.50 ± 9.08	46.42 ± 5.57	60.39 ± 4.83	1.7	1.3
Sorafenib	43.20 ± 4.55	6.15 ± 1.10	16.12 ± 2.81	7.0	2.6

a A549: human lung carcinoma cell line; LNCaP: human AR-expressing, androgen-sensitive prostate adenocarcinoma cell line; HUVEC: human umbilical vein endothelial cells. IC_50_ values are the means ± SD for at least three separate experiments. SI was defined as IC_50_(HUVEC)/IC_50_(cancer cells). A larger SI value denotes higher selectivity against cancer cells.

The cytotoxicity findings showed that the series of molecules possessed a unique cell line- and substituent-specific biological profile. Even though the test molecules were tested against A549 and LNCaP cell lines, their most characteristic biological profile could be seen with LNCaP (prostate cancer) cells, where many derivatives possessed low-micromolar cytotoxicity along with high selectivity compared with HUVEC cells. However, unlike LNCaP derivatives, the activity of the molecules against the A549 cell line was relatively weak, except for 5p, which had an IC_50_ of 9.45 µM and SI of 6.6 against A549 cells. This profile differed from those of the most LNCaP-selective variants, indicating that the biological response of the entire series was heavily influenced by the cell environment.

In summary, the biological results showed that the 5b–5d derivatives had a distinctly different cytotoxic profile as opposed to being uniformly antiproliferative. In particular, several derivatives exhibited a preferential cytotoxic effect towards LNCaP cells. The best profile was observed in the case of derivative 5d, followed by 5b and 5c, which showed potent low-micromolar LNCaP activity along with a significant increase in selectivity compared with sorafenib. The different selectivities of derivatives such as 5p (which selectively targeted A549 cells) and 5b–5d derivatives (which selectively targeted LNCaP cells) demonstrated that the R substituent could significantly affect cellular selectivity. Hence, molecular docking, MD, MM-GBSA, ADME and DFT analyses were performed on derivatives demonstrating relevant profiles.

#### 2.2.1 Structure–activity relationship (SAR)

Of all the LNCaP-targeted compounds, 5d emerged as the most prominent derivative, having an IC_50_ of 2.50 µM and an SI of 33.7 for HUVEC/LNCaP cells. These data clearly implied that incorporation of a 2,3-dichlorophenyl group into the structure yielded the best results with regard to potency and selectivity. Derivatives 5b and 5c, having 2,4-dimethylphenyl and 2,6-dimethylphenyl, respectively, were also potent LNCaP agents, possessing an IC_50_ of 7.96 and 5.30 µM, and SI of 16.5 and 13.8, respectively. These three derivatives were highly selective against LNCaP cells as compared with the reference compound sorafenib, possessing an IC_50_ of 16.12 µM and SI of 2.6 for LNCaP cells.

The extended substituent series also showed the dependence of the identity and position of the R group upon LNCaP activity. The unsubstituted phenyl compound 5l also exhibited good activity (IC_50_ = 8.51 µM; SI = 4.7), suggesting that the aromatic ring *per se* did not adversely affect the activity against LNCaP cells. Introduction of a methyl group to various positions yielded different patterns, wherein dimethyl groups in the 2,6-position (5c) and 2,4-position (5b) significantly enhanced selectivity, while the 2-position methyl compound 5k was inactive and nonselective (SI = 0.8). Likewise, positional effects were evident among chloro- and fluoro-substituted derivatives. The 2-chlorophenyl derivative 5i showed moderate LNCaP activity and selectivity (15.33 µM; SI = 4.1), whereas the 3-chloro analogue 5m was less active (24.85 µM; SI = 1.5). Fluoro derivatives 5j and 5r were relatively unfavorable, with their SI being 1.5 and 0.8, respectively, and the 4-bromophenyl derivative 5t was only slightly selective towards LNCaP cells (SI = 1.3). Therefore, there was no direct link between activity and halogenation, and substitution position seemed to be an important factor influencing biological profile.

In general, non-aromatic derivatives did not display any favorable LNCaP-selective profiles. For example, the cyclohexyl derivative 5h was characterized by an SI of 0.7, and the isopropyl derivative 5o had an SI of 0.9. *Tert*-butyl derivative 5p, although possessing excellent activity and selectivity towards A549 cells, demonstrated only moderate activity against LNCaP cells (21.02 µM; SI = 3.0). The benzyl derivative 5g had an unfavorable LNCaP selectivity (SI = 0.6), while addition of a *para*-fluoro substituent into 5q resulted in an increase in the biological profile of this compound to SI = 1.8 without producing the highly LNCaP-selective compound as 5b–5d. Methyl derivative 5s was another example where a very simple substituent led to moderate LNCaP activity (25.81 µM) and SI = 3.8 (Fig. 2).

**Fig. 2:**
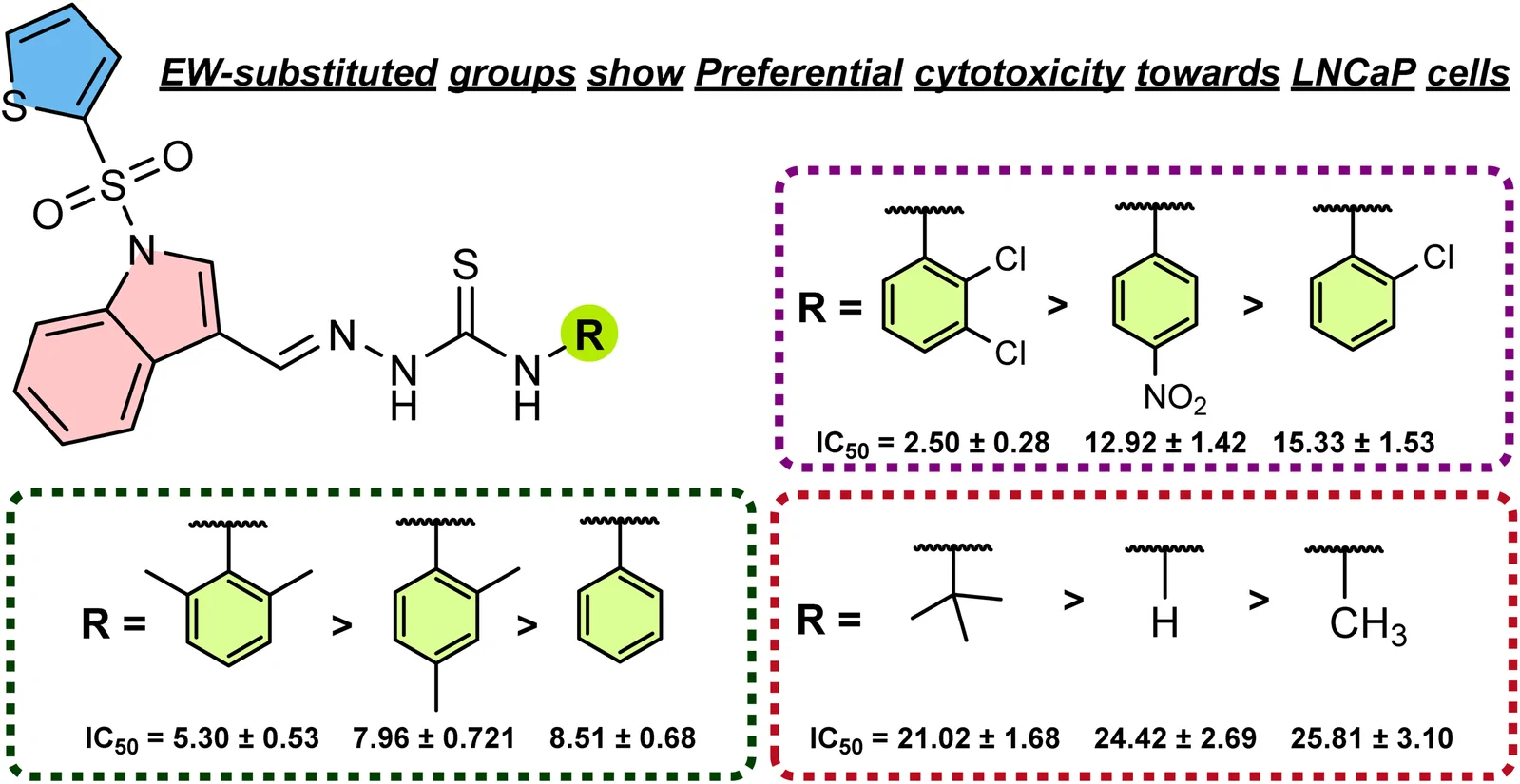
SAR of target compounds.

### 2.3 Molecular docking studies

Molecular docking studies were conducted against different molecular targets associated with cancer to analyse the underlying molecular basis of the preferentially observed cytotoxic activity against LNCaP cell lines. While the entire compound series was assessed against all the targeted molecules, the docking data described herein focus only on the three compounds, 5b, 5c, and 5d, which exhibited the most favorable combination of LNCaP potency and HUVEC/LNCaP selectivity in biological studies.

In addition to structural considerations, selection of the LNCaP-selective derivatives was based on their relative biological efficacy. None of the synthesized compounds exhibited improved biological activity against the A549 cancer cell line relative to the parent compound, while 5b–5d displayed considerably better LNCaP selectivity than sorafenib. Therefore, the subsequent molecular docking study was focused on the discovery of the possible molecular mechanisms of the LNCaP selectivity, rather than emphasizing the less distinctive A549 activity.

The chosen targets were AR, EGFR, VEGFR2, AKT1, mTOR, and PI3Kα (P110α). They represent important molecular points that are responsible for the development of prostate cancer, as well as general pathways that control cell proliferation, survival, angiogenesis, and metabolism. Special attention was paid to AR because LNCaP cells have AR- and androgen-dependent proliferation. The remaining targets were included to assess whether compounds 5b–5d could simultaneously engage other major oncogenic signaling pathways. Thus, the docking results were interpreted in conjunction with the experimental cytotoxicity and selectivity data to provide a structure-based perspective on the potential molecular mechanisms underlying the observed LNCaP-selective activity.

The docking results provided further support for the biological prioritization of compounds 5b–5d, particularly in the context of their LNCaP-selective cytotoxicity. Among the evaluated targets, AR showed consistently favorable docking profiles for all three compounds, with induced fit docking (IFD) scores of −13.324, −12.902, and −13.422 kcal mol^−1^ for 5b, 5c and 5d, respectively. Compound 5d exhibited the most favorable AR docking score among the synthesized derivatives (−13.422 kcal mol^−1^) and also showed the strongest predicted AR binding according to MM-GBSA (−99.05 kcal mol^−1^). Although sorafenib displayed a more favorable AR docking score (−14.865 kcal mol^−1^), its MM-GBSA (−85.96 kcal mol^−1^) was less favorable than that of compound 5d.

Compounds 5b and 5c also showed strong predicted interactions with AR, with MM-GBSA values of −93.07 and −85.66 kcal mol^−1^, respectively. Importantly, compound 5b displayed a favorable computational profile across several targets, including EGFR (−11.197 kcal mol; −87.58 kcal mol^−1^), AKT1 (−11.066 kcal mol; −79.50 kcal mol^−1^), and AR. Compound 5c showed particularly favorable MM-GBSA values for EGFR (−88.02 kcal mol^−1^) and mTOR (−80.64 kcal mol^−1^), while compound 5d demonstrated strong predicted binding towards AR, EGFR, VEGFR2, and mTOR. The MM-GBSA values reported in Table 2 were calculated using a single protein–ligand pose generated by the IFD protocol and, therefore, represent single-pose estimates rather than trajectory-averaged binding energy values.

**Table 2:** IFD docking scores and MM-GBSA binding free energies of compounds 5b–5d against selected prostate- and cancer-associated molecular targets

Target	IFD docking scores (kcal mol^−1^)						MM-GBSA Δ*G*_bind_ (kcal mol^−1^)					
	AR	EGFR	VEGFR2	AKT1	mTOR	P110α	AR	EGFR	VEGFR2	AKT1	mTOR	P110α
PDB	2PNU	3W2S	4ASE	4EJN	4JT5	4L23	2PNU	3W2S	4ASE	4EJN	4JT5	4L23
5b	−13.324	−11.197	−10.846	−11.066	−9.176	−8.271	−93.07	−87.58	−71.47	−79.50	−57.80	−47.91
5c	−12.902	−10.538	−10.815	−11.008	−11.672	−8.459	−85.66	−88.02	−66.73	−55.63	−80.64	−68.33
5d	−13.422	−9.394	−10.250	−10.675	−10.749	−8.472	−99.05	−81.60	−70.75	−69.49	−71.65	−53.27
Srf	−14.865	−10.240	−13.315	−10.833	−8.791	−8.674	−85.96	−88.08	−95.23	−76.64	−47.63	−60.77

The comparison with sorafenib also indicated that the computational behavior of compounds 5b–d was target-dependent rather than uniformly superior to the reference drug. Sorafenib showed better docking performance on AR and VEGFR2, whereas several synthesized ligands exhibited higher binding energies of MM-GBSA for AR and mTOR. Specifically, the highest AR-MM-GBSA interaction energy of −99.05 kcal mol^−1^ from compound 5d showed the best protein–ligand interaction energy among all compounds 5b–5d against these targets. These results revealed that AR was especially important in regard to the selective action of the synthesized LNCaP ligands, while their favorable interaction with EGFR, AKT1, mTOR, VEGFR2, and P110α might reflect the involvement of multiple signaling pathways in their mechanism of action. The subsequent interaction analysis, therefore, focused on elucidating the molecular recognition of compounds 5b–5d, with particular emphasis on their interactions within the AR binding site.

#### 2.3.1 Ligand–protein interaction analysis on AR

To further characterize the predicted binding mode of the most promising LNCaP-selective derivative, compound 5d was selected for detailed ligand–protein interaction analysis within the AR binding site. The analysis focused on the key amino acid residues and non-covalent interactions contributing to ligand recognition and binding stability. The 2D and 3D ligand–protein interaction modes of compound 5d within the AR binding site are presented in Fig. 3.

**Fig. 3:**
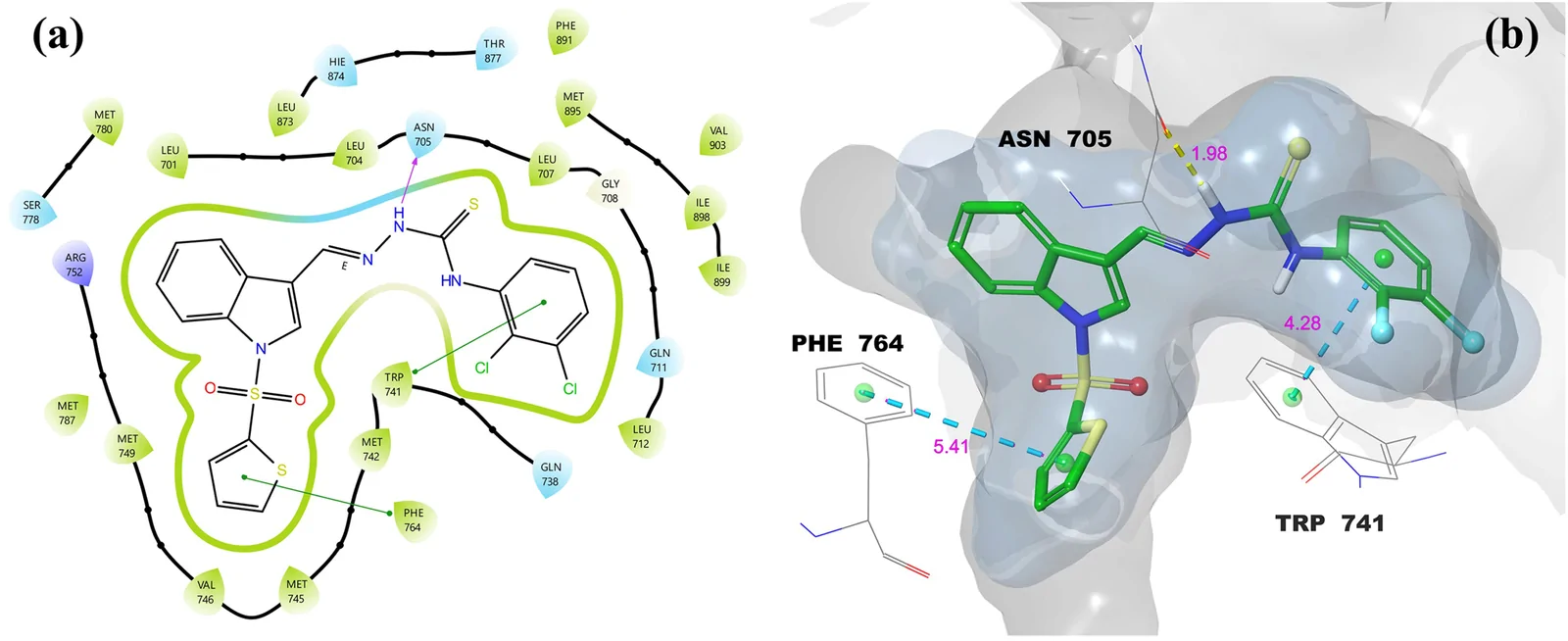
(a) 2D and (b) 3D ligand–protein interactions of compound 5d within the androgen receptor (AR) binding site.

Compound 5d adopted a favorable orientation within the AR binding cavity, allowing its key functional groups to establish complementary interactions with the surrounding residues (Fig. 3). The thiosemicarbazone moiety formed a hydrogen bond with Asn-705 (1.98 Å), which may serve as an important anchoring interaction and help maintain the ligand in an appropriate position within the pocket. In parallel, the aromatic segments of the molecule formed π–π interactions with Trp-741 and Phe-764, offering further stabilization *via* aromatic recognition. The 3D representation further demonstrated that these interactions occurred within a well-defined protein binding region, with the ligand surface appropriately accommodated in the corresponding cavity. Thus, the combination of a directional hydrogen bond with complementary π–π stacking interactions suggested that Asn-705, Trp-741, and Phe-764 collectively contributed to the recognition and stabilization of compound 5d in the AR binding site, supporting its potential relevance to the observed LNCaP-selective activity.

#### 2.3.2 MM-GBSA energy decomposition analysis

To further characterize the energetic determinants of AR recognition by the most promising LNCaP-selective derivatives, an MM-GBSA energy decomposition analysis was performed for compounds 5b–5d. This analysis was used to identify the individual energetic contributions governing ligand binding and to determine which interaction components predominantly supported the predicted affinity of these compounds toward AR (Table 3).

**Table 3:** MM-GBSA energy decomposition components for compounds 5b–5d in complex with AR

Parameters (kcal mol^−1^)	5b	5c	5d
MM-GBSA Δ*G*_bind_ Coulomb	7.94	3.76	3.46
MM-GBSA Δ*G*_bind_ covalent	2.88	8.96	7.39
MM-GBSA Δ*G*_bind_ Hbond	−0.54	−0.45	−0.54
MM-GBSA Δ*G*_bind_ lipo	−38.04	−38.22	−38.34
MM-GBSA Δ*G*_bind_ SelfCont	0.00	0.00	0.00
MM-GBSA Δ*G*_bind_ solv GB	12.19	7.21	7.71

The energy decomposition analysis revealed that the lipophilic contribution was the dominant favorable component for all three compounds, with values of −38.04, −38.22, and −38.34 kcal mol^−1^ for compounds 5b, 5c, and 5d, respectively. This highly consistent contribution indicated that hydrophobic interactions within the AR binding cavity had a major role in stabilizing the complexes. Notably, compound 5d had the best lipophilic contribution (−38.34 kcal mol^−1^), which agreed well with the large amount of hydrophobic and aromatic interactions revealed in the ligand–protein interaction analysis.

The hydrogen-bonding contribution was also favorable and similar for 5b and 5d (−0.54 kcal mol^−1^), while it was slightly less favorable for compound 5c (−0.45 kcal mol^−1^). These data showed the contribution of polar interactions in ligand binding. On the contrary, the coulombic contribution as well as the covalent contribution was positive for all compounds, which meant that they were not the major factors contributing to the binding energy. The generalized Born solvation contributions were also positive, partially opposing the favorable interactions. MM-GBSA energy decomposition was performed using the initial protein–ligand complexes obtained from the IFD protocol, with each analysis based on a single IFD-generated pose rather than structures sampled from the MD trajectories.

### 2.4 MD simulations

The stability of the AR-complex with compound 5d was investigated through a 250 ns MD simulation. Protein–ligand stability was analyzed mainly through root mean square deviation (RMSD) and root mean square fluctuation (RMSF). RMSD and RMSF plots of the AR-complex with compound 5d are shown in Fig. 4.

**Fig. 4:**
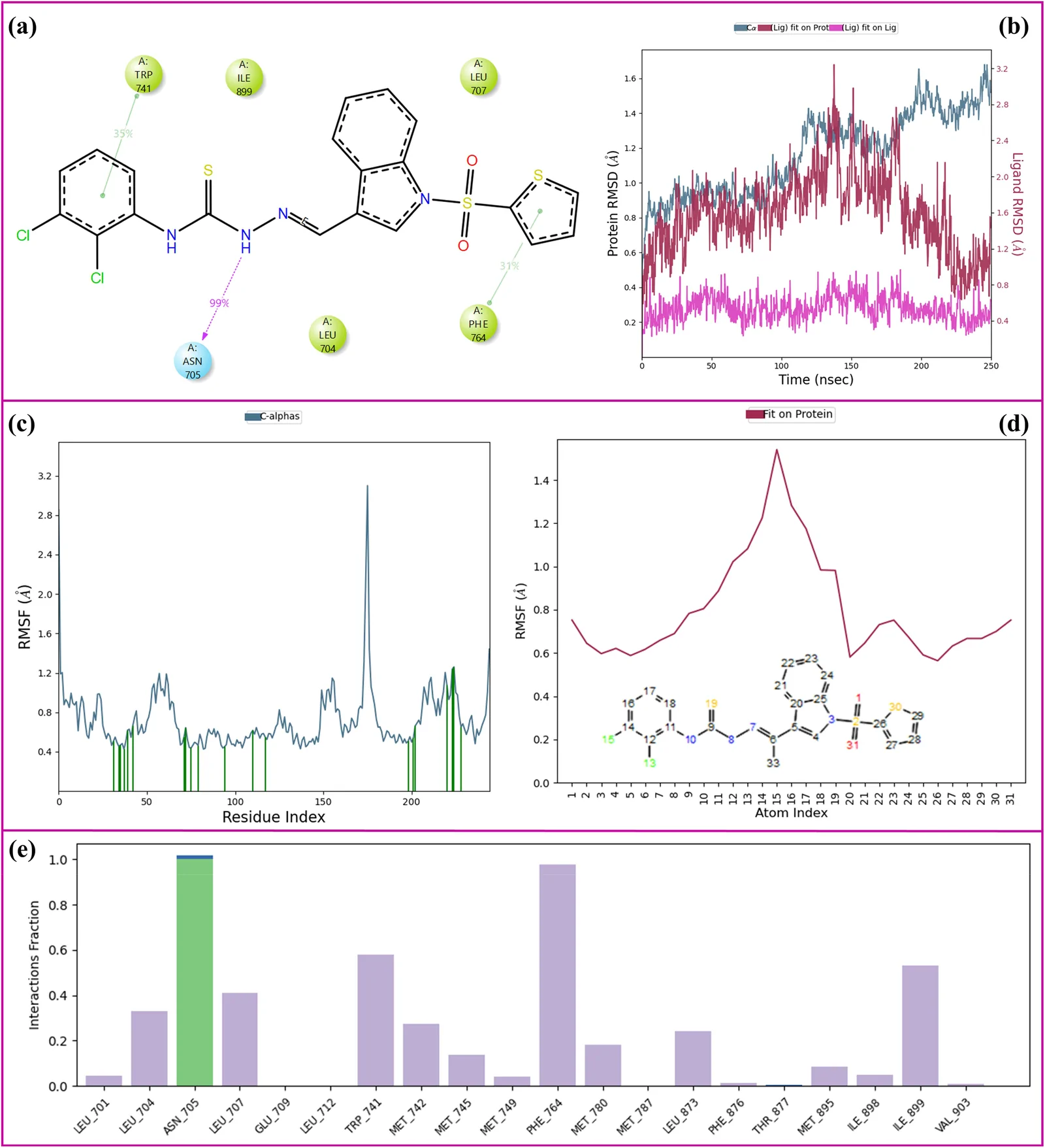
Analysis of the AR-ligand 5d complex by molecular dynamics simulations over 250 ns. (a) Important interactions between the ligand and protein during the simulation. (b) RMSD plot of protein Cα atoms and ligand. (c) RMSF plot of protein Cα atoms. (d) RMSF plot of ligand atoms. (e) Histogram denoting the interaction fraction showing the persistence of ligand–protein interactions throughout the simulation.

Fig. 4a shows the important protein–ligand interactions identified during the 250 ns MD simulation. Compound 5d maintained interactions with Leu-704 (33%), Asn-705 (99%), Leu-707 (41%), Trp-741 (58%), Met-742 (27%), Met-745 (14%), Phe-764 (97%), Met-780 (18%), Leu-873 (24%), Met-895 (8%), Ile-898 (5%), and Ile-899 (53%). Among these residues, Asn-705 and Phe-764 exhibited the highest level of interaction stability, followed by Trp-741 and Ile-899, which confirmed the important role of these residues in stabilizing compound 5d in the AR binding site. Fig. 4b shows the RMSD profiles of the protein and ligand throughout the simulation. The protein Cα RMSD remained within approximately 0.6–1.6 Å. Although the ligand RMSD after fitting to the protein increased transiently from ∼0.8 Å to 2.6–2.8 Å between 50 and 150 ns, it subsequently decreased. This fluctuation may reflect transient conformational or positional adjustments of compound 5d within the binding pocket during the simulation.

The RMSF plot of the Cα atoms of the protein is shown in Fig. 4c. Most residues exhibited relatively low fluctuations, generally not exceeding 1.2 Å. However, a more pronounced fluctuation of ∼3.1 Å was observed around residue 160, potentially reflecting increased flexibility in this region. This could possibly be because the region was flexible but did not show overall instability. Fig. 4d shows the RMSF plot of the compound 5d atoms. Most atoms showed fluctuations below 1.0 Å, while the central region of the ligand exhibited somewhat higher mobility, reaching ∼1.5 Å. Nevertheless, the relatively limited overall fluctuations suggested that compound 5d retained its binding-site conformation while allowing localized conformational flexibility.

Fig. 4e shows the interaction fraction histogram obtained throughout the MD trajectory. The most persistent interactions were observed with Asn-705 (99%), Phe-764 (97%), Trp-741 (58%), Ile-899 (53%), and Leu-707 (41%). These residues represent the major interaction contributors and are likely to play an important part in maintaining the stable accommodation of compound 5d within the AR binding pocket during the 250 ns simulation.

### 2.5 Post-MD MM-GBSA analysis based on MD trajectories

To complement the initial IFD-based MM-GBSA calculations and further investigate the binding energetics of compound 5d in complex with AR, an additional post-MD MM-GBSA analysis was performed using representative structures extracted from MD trajectories. Unlike the initial calculations, which were based on single IFD-generated poses, this analysis incorporated multiple conformations sampled during the 250 ns MD simulations. Fifty representative structures were selected at 5 ns intervals, and MM-GBSA calculations were performed for each structure. The resulting energy components were averaged to provide a trajectory-based assessment of the energetic contributions to ligand binding and to examine whether the favorable binding profiles observed in the initial IFD-based analysis were maintained during the simulations.

The post-MD MM-GBSA analysis of the 5d–AR complex yielded an average binding free energy of −96.26 ± 5.85 kcal mol^−1^ over the 250 ns MD trajectory. The consistently negative energy values suggested favorable ligand–receptor binding energetics. These findings complemented the initial IFD-based calculations by incorporating representative conformations sampled during the MD simulation. However, MM-GBSA results should be interpreted alongside RMSD profiles and protein–ligand interaction analyses to provide a more comprehensive assessment of complex stability.

### 2.6 ADME prediction

To further assess the drug-like properties of the most active LNCaP-selective compounds, compounds 5b–5d and sorafenib were subjected to *in silico* ADME prediction using QikProp. Key physicochemical and pharmacokinetic descriptors, including molecular weight, hydrogen-bonding capacity, permeability, lipophilicity, aqueous solubility, blood–brain barrier penetration, oral absorption, and Rule of Five and Rule of Three parameters, were evaluated to provide an initial assessment of their pharmacokinetic suitability. Results are given in Table 5.

**Table 4:** MM-GBSA binding free energies of the compound 5d–AR complex over a 250 ns MD simulation, expressed as mean ± SD

Time (ns)	MM-GBSA (kcal mol^−1^)	Time (ns)	MM-GBSA (kcal mol^−1^)	Time (ns)	MM-GBSA (kcal mol^−1^)
5	−106.13	90	−94.26	175	−95.71
10	−104.86	95	−92.17	180	−98.24
15	−103.74	100	−91.28	185	−96.38
20	−105.28	105	−90.37	190	−94.17
25	−104.39	110	−89.26	195	−92.46
30	−102.87	115	−88.36	200	−90.37
35	−103.91	120	−87.42	205	−91.26
40	−102.63	125	−89.16	210	−93.18
45	−103.17	130	−88.47	215	−95.42
50	−101.76	135	−87.26	220	−97.36
55	−100.83	140	−89.63	225	−99.24
60	−99.64	145	−87.84	230	−101.18
65	−98.72	150	−89.17	235	−102.73
70	−97.83	155	−90.76	240	−104.17
75	−96.91	160	−90.24	245	−103.28
80	−96.24	165	−91.82	250	−102.46
85	−94.83	170	−94.36	**Average**	**−96.26 ± 5.85**

**Table 5:** ADME prediction of the most potent compounds as well as sorafenib

ADME parameters	5b	5c	5d	Sorafenib	Reference values
mol *M*_W_	468.60	468.60	509.44	464.83	130–725
accptHB	9	9	9	6	2–20
donorHB	2	2	2	3	0–6
QPPCaco	714	951	899	323	<25 poor; >500 great
QPPMDCK	1203	1388	5800	1713	<25 poor; >500 great
QP log BB	−1.035	−0.766	−0.531	−0.977	−3.0 to 1.2
QP log *P*_o/w_	4.443	4.265	4.634	4.102	−2.0 to 6.5
QP log *S*	−7.035	−6.441	−7.028	−7.058	−6.5 to 0.5
Rule of Five	0	0	1	0	≤4 violations
Rule of Three	1	1	1	1	≤3 violations
Percent human oral absorption	100	100	93	95	>80 high; <25 poor
hERG	−7.647	−7.124	−7.314	−5.862	Concern below −5.0

The predicted ADME profiles of compounds 5b–5d were generally comparable to that of sorafenib and indicated acceptable drug-like properties (Table 4). All three compounds showed favorable hydrogen-bonding profiles and high predicted permeability, with QPPCaco values of 714–951 and QPPMDCK values of 1203–5800. Their predicted QP log BB values (−1.035 to −0.531) suggested limited brain penetration, while the relatively high QP log *P*_o/w_ values (4.265–4.634) indicated considerable lipophilicity. Although compounds 5b and 5d showed QP log *S* values slightly below the reference range, their predicted human oral absorption remained high (93–100%). Compounds 5b and 5c did not show Rule of Five violations, whereas compound 5d had one violation. Overall, the ADME predictions supported the favorable drug-like profile of the most potent compounds, with compound 5d displaying a profile broadly comparable to that of sorafenib. The predicted QP log HERG values for all compounds were below −5.0, indicating potential hERG-related safety concerns that require experimental validation.

### 2.7 DFT-based frontier molecular orbital, electrostatic potential (ESP) and global chemical reactivity (GCR) descriptor analyses

The electronic characteristics of compounds 5b–5d were investigated using DFT calculations at the B3LYP/6-31G** level. The HOMO and LUMO distributions, ESP maps, and GCR descriptors were analyzed to characterize their electronic structures and reactivity profiles. The frontier molecular orbitals and ESP surfaces for the above molecules are illustrated in Fig. 5, while the computed energy levels and GCR parameters for them are shown in Table 6.

**Fig. 5:**
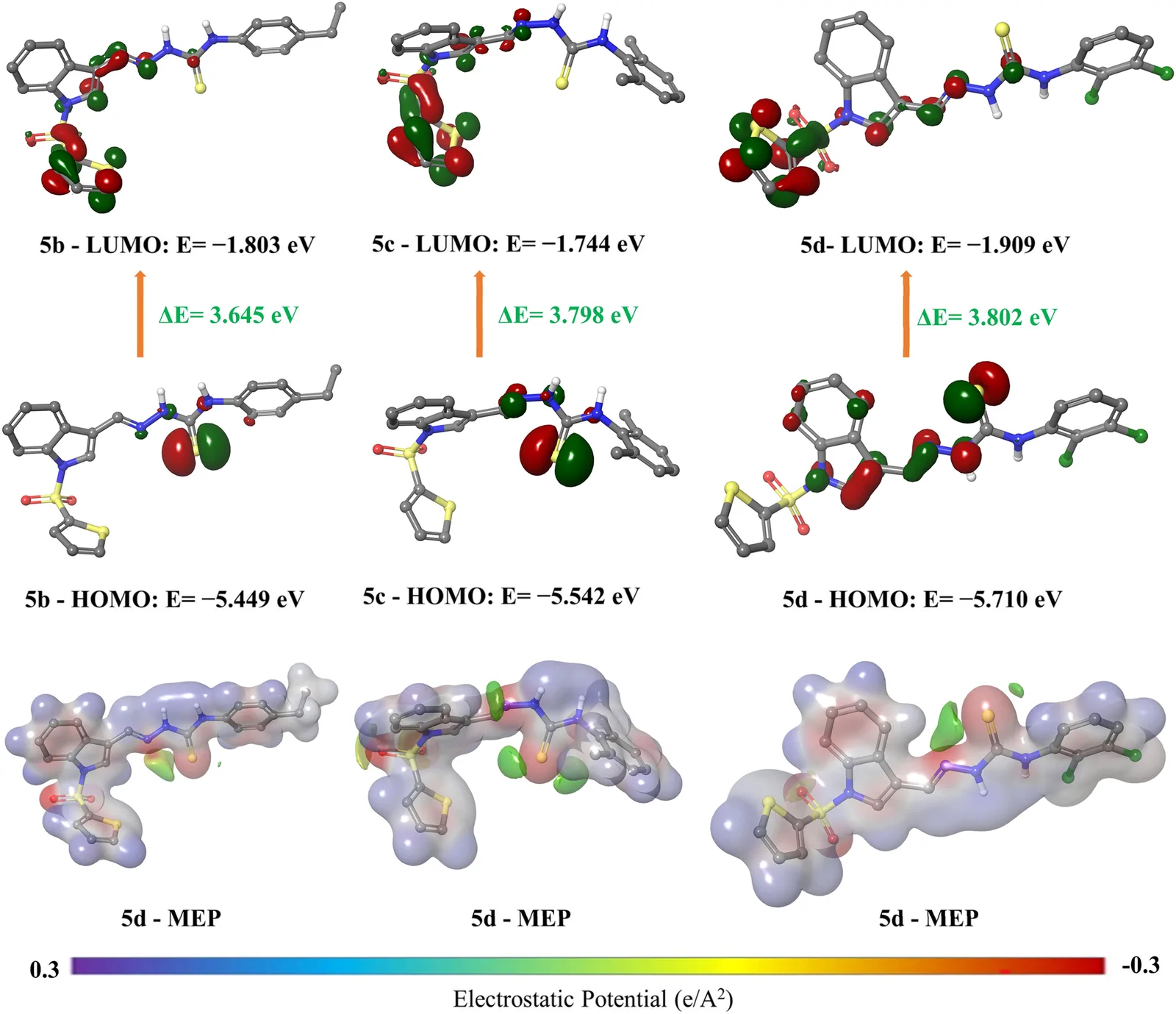
Frontier molecular orbitals and molecular electrostatic potential (MEP) maps of compounds 5b–5d.

**Table 6:** Frontier molecular orbital energies and GCR parameters of compounds 5b–5d using DFT calculations

Parameters^a^	Compound 5b	Compound 5c	Compound 5d
*E*_HOMO_ (eV)	−5.449	−5.542	−5.710
*E*_LUMO_ (eV)	−1.803	−1.744	−1.909
Δ*E* (LUMO–HOMO) (eV)	3.645	3.798	3.802
Ionization potential (*I*) (eV)	5.449	5.542	5.710
Electron affinity (*A*) (eV)	1.803	1.744	1.909
Electronegativity (*χ*) (eV)	3.626	3.643	3.809
Chemical potential (*µ*) (eV)	−3.626	−3.643	−3.809
Global hardness (*η*) (eV)	1.823	1.899	1.901
Global softness (*S*) (eV^−1^)	0.274	0.263	0.263
Electrophilicity index (*ω*) (eV)	3.606	3.495	3.817

a *E*_HOMO_, energy of the highest occupied molecular orbital; *E*_LUMO_, energy of the lowest unoccupied molecular orbital; Δ*E* = *E*_LUMO_ − *E*_HOMO_; *I* (ionization potential) = −*E*_HOMO_; *A* (electron affinity) = −*E*_LUMO_; *χ* (electronegativity) = (*I* + *A*)/2; *µ* (chemical potential) = −*χ*; *η* (global hardness) = (*I* − *A*)/2; *S* (global softness) = 1/(2*η*); *ω* (electrophilicity index) = *µ*^2^/(2*η*). All energies are expressed in eV and calculated according to Koopman's theorem.

The DFT results indicated that the electronic properties of compounds 5b–5d were generally similar, with HOMO–LUMO energy gaps of 3.645, 3.798, and 3.802 eV, respectively. Compound 5b exhibited the smallest energy gap and the highest global softness (0.274 eV^−1^), suggesting comparatively greater electronic softness than compounds 5c and 5d. In contrast, compound 5d had the lowest HOMO energy (−5.710 eV), as well as the highest electronegativity (3.809 eV) and electrophilicity index (3.817 eV), indicating a comparatively stronger electron-accepting tendency. The similar hardness values (1.823–1.901 eV) indicated broadly comparable electronic resistance to deformation among the three derivatives. Overall, the DFT descriptors revealed modest (but distinguishable) differences in their electronic properties, with compound 5d exhibiting the highest electrophilicity index.

It was established from the analysis of frontier molecular orbital densities for compounds 5b–5d that HOMO and LUMO densities were localized on the heteroatomic and conjugated regions within the molecule, specifically on the sulfonyl and thiosemicarbazone groups. The HOMO–LUMO gap of compound 5b was the smallest (3.645 eV) among the compounds, suggesting its relative softness in comparison with compounds 5c and 5d. Compounds 5c and 5d had very close gaps of 3.798 and 3.802 eV, which corresponded to their similar electronic behavior. Notably, compound 5d displayed the most stabilized HOMO (−5.710 eV) and LUMO (−1.909 eV) levels.

In addition to the above, MEP maps showed electrostatic negativity associated with oxygen, sulfur, and nitrogen atoms, and electrostatic positivity associated with the hydrogen-containing parts of the molecules. Hence, the regions containing heteroatoms were the most active sites for electrostatic interactions and potential hydrogen-bond acceptance or donation. In general, the orbital and MEP maps showed that the three molecules had similar electronic properties; however, the slightly different localization and energies of the frontier orbitals could lead to their unique abilities for molecular recognition.

### 2.8 Molecular docking validations

The accuracy of the molecular docking procedure was evaluated by redocking the native ligands co-crystallized with the receptors into their respective binding sites using the same docking procedure as that used for the studied compounds. The calculated RMSD values were 0.1525 Å for AR (2PNU), 1.9344 Å for EGFR (3W2S), 0.0890 Å for VEGFR-2 (4ASE), 1.0943 Å for AKT1 (4EJN), 0.1923 Å for mTOR (4JT5), and 0.1867 Å for PI3Kα (4L23). The RMSD of all the results was <2.0 Å, which is considered the threshold for acceptable results. Hence, the experimentally determined conformation of the ligands was similar to the redocked one. This demonstrated that the docking procedure used was reliable and reproducible.

### 2.9 Statistical analyses

All experiments were performed at least thrice (*n* ≥ 3). Results are the mean ± standard deviation (SD). IC_50_ values were determined from concentration–response data. Statistical comparisons among multiple experimental groups were performed using one-way analysis of variance (ANOVA), followed by Tukey's *post hoc* multiple-comparison test if a significant overall difference was detected. Comparisons between individual compounds and the reference drug were evaluated using the same multiple-comparison procedure. *P* < 0.05 was considered significant.

### 2.10 Limitations

We described the biological potential of the thiophene–indole scaffold, but the effect of substituents on the thiophene and indole rings was beyond the scope of the present work. Future studies involving systematic modification of these rings and using heterocyclic thiosemicarbazide could provide deeper insight into the SAR and facilitate further optimization of biological activity. Cytotoxicity was assessed only after 24 h of compound exposure, so the effects of longer treatment periods on IC_50_ values remain to be investigated.

## 3 Conclusions

A series of thiophen-2-ylsulfonyl indole-3-carbaldehyde thiosemicarbazones was synthesized and characterized using *in vitro* and *in silico* methods. There was cytotoxicity against cancer cell lines depending on their nature and substituent position, with the LNCaP (prostate cancer) line showing the strongest response. Compound 5d emerged as the lead candidate (IC_50_ = 2.50 ± 0.28 µM; SI = 33.7 *vs.* HUVEC cells), while 5b and 5c also showed notable LNCaP potency (IC_50_ = 7.96 ± 0.72 and 5.30 ± 0.53 µM; SI = 16.5 and 13.8), all surpassing the selectivity profile of sorafenib in this model. Several compounds exhibited promising cytotoxic activity against A549 cells, but none showed greater potency than sorafenib in this cell line. Therefore, our findings do not support a claim of superior anti-A549 activity or broader dual-cancer utility.

Based on molecular docking and MM-GBSA study of 5b–5d against cancer-related targets, AR was revealed to be the likely target of these derivatives, where 5d showed specific and stable interactions with Asn-705, Phe-764, Trp-741, and Ile-899 during 250 ns MD simulation. In the case of ADME evaluation, 5b–5d exhibited good pharmacokinetic profiles with drug-likeness and oral availability, but solubility in water needs optimization in future work. DFT calculations provided additional information about the reactivity of the lead compounds. Collectively, these findings position 5d as a promising AR-targeted lead for prostate cancer, warranting future biochemical, mechanistic, and *in vivo* validation.

## 4 Experimental section

### 4.1 General

All chemicals and solvents necessary for the synthesis of thiosemicarbazone derivatives starting from 1*H*-indole-3-carbaldehyde (97%), thiophene-2-sulfonyl chloride (98%), petroleum ether (99%), glacial acetic acid (99.7%), triethylamine (99.5%), ethyl acetate (99.5%) and ethanol (99.9%) were purchased from Merck. Reaction progress was monitored by TLC analysis of the products on silica-coated aluminum plates. Structural studies of the synthesized compounds were done through ^1^H and ^13^C NMR spectroscopic methods at 25 °C using the Bruker Ascend 600 MHz NMR spectrometer operating at 400 MHz for ^1^H and 101 MHz for ^13^C with DMSO-*d*_6_ and CDCl_3_ as solvents. Chemical shifts are provided in ppm, *J* coupling constants in Hz, while multiplicities are indicated appropriately. HRMS analyses of the synthesized compounds to check their molecular masses were performed using the Thermo Fisher Scientific Q Exactive™ Hybrid Quadrupole-Orbitrap™ mass spectrometer.

### 4.2 Synthesis of target compounds

#### 4.2.1 General procedure for the synthesis of 1-(thiophen-2-ylsulfonyl)-1*H*-indole-3-carbaldehyde (3)

1-(Thiophen-2-ylsulfonyl)-1*H*-indole-3-carbaldehyde (3) was synthesized by adding 1*H*-indole-3-carbaldehyde (1) (10 mmol) and triethylamine (10 mL) in a flask. The solution was stirred at 90 °C for 30 min. After that, the solution of thiophene-2-sulfonyl chloride (2) (15 mmol) in TEA (2 mL) was added to the reaction mixture and further heated at 90 °C for 2 h. TLC was employed to check the reaction progress. Upon completion, the resulting mixture was poured into iced distilled water with constant stirring. The resulting precipitates were filtered and washed with cold H_2_O and dried under vacuum at room temperature^55^ to yield 1-(thiophen-2-ylsulfonyl)-1*H*-indole-3-carbaldehyde as a dark-brown solid.

#### 4.2.2 General procedure for the synthesis of thiosemicarbazones 5a–5t

Indole-based thiosemicarbazones 5a–5t were synthesized by reacting 1-(thiophen-2-ylsulfonyl)-1*H*-indole-3-carbaldehyde (3) (1 mmol) with substituted thiosemicarbazides 4a–t (1 mmol) in ethanol (10 mL) by adding acetic acid (2–3 drops) for 3–4 h at 60–70 °C. Reaction progress was monitored *via* TLC. The resulting precipitates were filtered, rinsed with ethanol, and dried. The targeted thiosemicarbazones 5a–5t were obtained in excellent yields (75–85%).

The resulting compounds were characterized as detailed below.

##### 4.2.2.1 (*E*)-*N*-(4-Ethylphenyl)-2-{[1-(thiophen-2-ylsulfonyl)-1*H*-indol-3-yl]methylene}hydrazine-1-carbothioamide (5a)

Light-brown; m.p.: 208–210 °C; yield: 77% ^1^H NMR (400 MHz, CDCl_3_) *δ* 10.49 (s, 1H, NH), 8.87 (s, 1H, NH), 8.18 (s, 1H, CH), 8.06 (t, *J* = 8.5 Hz, 2H, Ar–H), 7.85 (s, 1H, Ar–H), 7.74 (dd, *J* = 11.9, 4.2 Hz, 2H, Ar–H), 7.60 (d, *J* = 5.0 Hz, 1H, Ar–H), 7.43 (d, *J* = 7.9 Hz, 1H, Ar–H), 7.33 (q, *J* = 5.3 Hz, 4H, Ar–H), 7.04 (t, *J* = 4.4 Hz, 1H, Ar–H), 2.74 (d, *J* = 7.6 Hz, 2H, CH_2_), 1.28 (s, 3H, CH_3_). ^13^C NMR (101 MHz, CDCl_3_) *δ* 176.74 (C=S), 139.74 (HC = N), 137.81 (Ar), 137.64 (Ar), 135.85 (Ar), 135.56 (Ar), 134.56 (Ar), 134.08 (Ar), 129.80 (Ar), 129.08 (Ar), 128.14 (Ar), 127.96 (Ar), 127.87 (Ar), 127.06 (Ar), 126.59 (Ar), 126.30 (Ar), 124.68 (Ar), 122.42 (Ar), 117.74 (Ar), 114.02 (Ar), 24.76 (CH_2_), 14.63 (CH_3_). ESI-HRMS (*m*/*z*): chemical formula: C_22_H_20_N_4_O_2_S_3_, calculated [M + H]^+^: 469.08266, found [M + H]^+^: 469.08160.

##### 4.2.2.2 (*E*)-*N*-(2,4-Dimethylphenyl)-2-{[1-(thiophen-2-ylsulfonyl)-1*H*-indol-3-yl]methylene}hydrazine-1-carbothioamide (5b)

Off-white; m.p: 220–222 °C; yield: 83%; ^1^H NMR (400 MHz, CDCl_3_) *δ* 10.34 (s, 1H, NH), 8.75 (s, 1H, NH), 8.14 (s, 1H, CH), 8.06 (t, *J* = 7.4 Hz, 2H, Ar–H), 7.85 (s, 1H, Ar–H), 7.75 (d, *J* = 3.9 Hz, 1H, Ar–H), 7.60 (d, *J* = 5.0 Hz, 1H, Ar–H), 7.53 (d, *J* = 7.9 Hz, 1H, Ar–H), 7.44 (t, *J* = 7.8 Hz, 1H, Ar–H), 7.34 (t, *J* = 7.6 Hz, 1H, Ar–H), 7.12 (d, *J* = 8.2 Hz, 2H, Ar–H), 7.04 (t, *J* = 4.5 Hz, 1H, Ar–H), 2.35 (d, *J* = 12.2 Hz, 6H, [CH_3_]_2_). ^13^C NMR (101 MHz, CDCl_3_) *δ* 176.13 (C=S), 137.02 (HC = N), 136.98 (Ar), 136.95 (Ar), 134.95 (Ar), 133.92 (Ar), 133.58 (Ar), 133.44 (Ar), 133.25 (Ar), 131.00 (Ar), 129.08 (Ar), 127.33 (Ar), 126.95 (Ar), 126.78 (Ar), 126.46 (Ar), 125.68 (Ar), 124.10 (Ar), 121.82 (Ar), 117.18 (Ar), 113.39 (Ar), 20.63 (CH_3_), 17.52 (CH_3_). ESI-HRMS (*m*/*z*): chemical formula: C_22_H_20_N_4_O_2_S_3_, calculated [M + H]^+^: 469.08266, found [M + H]^+^: 469.08129.

##### 4.2.2.3 (*E*)-*N*-(2,6-Dimethylphenyl)-2-{[1-(thiophen-2-ylsulfonyl)-1*H*-indol-3-yl]methylene}hydrazine-1-carbothioamide (5c)

Light-brown; m.p.: 238–240 °C; yield: 83%; ^1^H NMR (400 MHz, CDCl_3_) *δ* 10.84 (s, 1H, NH), 8.54 (s, 1H, NH), 8.22 (s, 1H, CH), 8.06 (dd, *J* = 8.1, 4.6 Hz, 2H, Ar–H), 7.86 (s, 1H, Ar–H), 7.77 (dd, *J* = 3.8, 1.3 Hz, 1H, Ar–H), 7.62 (dd, *J* = 5.0, 1.3 Hz, 1H, Ar–H), 7.46 (t, *J* = 7.6 Hz, 1H, Ar–H), 7.37 (t, *J* = 7.6 Hz, 1H, Ar–H), 7.28 (s, 1H, Ar–H), 7.22 (d, *J* = 6.7 Hz, 2H, Ar–H), 7.09–7.03 (m, 1H, Ar–H), 2.38 (s, 6H, [CH_3_]_2_). ^13^C NMR (101 MHz, CDCl_3_) *δ* 177.09 (C=S), 137.84 (HC = N), 137.63 (Ar), 137.08 (Ar), 135.56 (Ar), 135.47 (Ar), 134.52 (Ar), 134.05 (Ar), 129.75 (Ar), 128.50 (Ar), 127.94 (Ar), 127.09 (Ar), 126.27 (Ar), 124.80 (Ar), 122.45 (Ar), 117.86 (Ar), 113.99 (Ar), 18.63 (CH_3_). ESI-HRMS (*m*/*z*): chemical formula: C_22_H_20_N_4_O_2_S_3_, calculated [M + H]^+^: 469.08266, found [M + H]^+^: 469.08233.

##### 4.2.2.4 (*E*)-*N*-(2,3-Dichlorophenyl)-2-{[1-(thiophen-2-ylsulfonyl)-1*H*-indol-3-yl]methylene}hydrazine-1-carbothioamide (5d)

Off-white; m.p.: 232–234 °C; yield: 81%; ^1^H NMR (400 MHz, DMSO) *δ* 12.14 (s, 1H, NH), 9.80 (s, 1H, NH), 8.41 (d, *J* = 6.9 Hz, 2H, Ar–H), 8.37 (d, *J* = 7.9 Hz, 1H, CH), 8.08 (dd, *J* = 5.0, 1.4 Hz, 1H, Ar–H), 8.02 (dd, *J* = 3.9, 1.4 Hz, 1H, Ar–H), 7.97 (dt, *J* = 8.4, 0.9 Hz, 1H, Ar–H), 7.82 (dd, *J* = 8.1, 1.5 Hz, 1H, Ar–H), 7.57 (dd, *J* = 8.1, 1.5 Hz, 1H, Ar–H), 7.48 (d, *J* = 1.2 Hz, 1H, Ar–H), 7.43–7.32 (m, 2H, Ar–H), 7.20 (dd, *J* = 4.9, 3.9 Hz, 1H, Ar–H). ^13^C NMR (101 MHz, DMSO) *δ* 176.04 (C=S), 138.86 (HC = N), 138.60 (Ar), 136.99 (Ar), 135.89 (Ar), 135.29 (Ar), 134.65 (Ar), 131.61 (Ar), 130.32 (Ar), 128.96 (Ar), 128.62 (Ar), 128.32 (Ar), 128.05 (Ar), 127.59 (Ar), 126.70 (Ar), 126.07 (Ar), 124.57 (Ar), 123.28 (Ar), 117.99 (Ar), 113.18 (Ar). ESI-HRMS (*m*/*z*): chemical formula: C_20_H_14_Cl_2_N_4_O_2_S_3_, calculated [M + H]^+^: 508.97342, found [M + H]^+^: 508.97387; HPLC-PDA: *λ*_max_, MeCN : MeOH (1 : 1), flow rate 5.0 mL min^−1^, pressure 154 bar, *R*_t_: 4.72 min, purity: 97.96%.

##### 4.2.2.5 (*E*)-*N*-(4-Nitrophenyl)-2-{[1-(thiophen-2-ylsulfonyl)-1*H*-indol-3-yl]methylene}hydrazine-1-carbothioamide (5e)

Yellow; m.p.: 187–189 °C; yield: 78%; ^1^H NMR (400 MHz, DMSO) *δ* 12.24 (s, 1H, NH), 10.24 (s, 1H, NH), 8.44 (s, 2H, CH), 8.25 (d, *J* = 7.7 Hz, 3H, Ar–H), 8.07 (dd, *J* = 15.7, 6.7 Hz, 4H, Ar–H), 7.98 (d, *J* = 8.1 Hz, 1H, Ar–H), 7.48 (t, *J* = 7.5 Hz, 1H, Ar–H), 7.40 (t, *J* = 6.4 Hz, 1H, Ar–H), 7.20 (d, *J* = 3.9 Hz, 1H, Ar–H). ^13^C NMR (101 MHz, DMSO) *δ* 175.33 (C=S), 145.63 (HC = N), 143.33 (Ar), 139.18 (Ar), 136.99 (Ar), 135.85 (Ar), 135.28 (Ar), 134.63 (Ar), 130.14 (Ar), 128.59 (Ar), 126.87 (Ar), 126.03 (Ar), 124.61 (Ar), 124.03 (Ar), 123.89 (Ar), 123.06 (Ar), 118.03 (Ar), 113.20 (Ar). ESI-HRMS (*m*/*z*): chemical formula: C_20_H_14_N_5_O_4_S_3_, calculated [M − H]^+^: 484.02079, found [M + H]^+^: 484.02198.

##### 4.2.2.6 (*E*)-*N*-(4-Methoxyphenyl)-2-{[1-(thiophen-2-ylsulfonyl)-1*H*-indol-3-yl]methylene}hydrazine-1-carbothioamide (5f)

Brown; m.p.: 203–205 °C; yield: 77%; ^1^H NMR (400 MHz, CDCl_3_) *δ* 9.86 (s, 1H, NH), 8.90 (s, 1H, NH), 8.08 (s, 1H, CH), 8.07–8.03 (m, 2H, Ar–H), 7.86 (s, 1H, Ar–H), 7.76 (dd, *J* = 3.9, 1.4 Hz, 1H, Ar–H), 7.61 (dd, *J* = 5.0, 1.4 Hz, 1H, Ar–H), 7.51 (d, *J* = 8.8 Hz, 2H, Ar–H), 7.45 (t, *J* = 7.8 Hz, 1H, Ar–H), 7.37 (t, *J* = 7.6 Hz, 1H, Ar–H), 7.04 (dd, *J* = 5.0, 3.9 Hz, 1H, Ar–H), 6.98–6.95 (m, 2H, Ar–H), 3.84 (s, 3H, CH_3_). ^13^C NMR (101 MHz, CDCl_3_) *δ* 176.50 (C=S), 158.30 (HC = N), 137.63 (Ar), 137.29 (Ar), 135.56 (Ar), 134.58 (Ar), 134.09 (Ar), 130.83 (Ar), 129.50 (Ar), 127.97 (Ar), 126.98 (Ar), 126.33 (Ar), 124.80 (Ar), 122.34 (Ar), 117.69 (Ar), 114.28 (Ar), 114.02 (Ar), 55.66 (O–CH_3_). ESI-HRMS (*m*/*z*): chemical formula: C_21_H_18_N_4_O_3_S_3_, calculated [M + H]^+^: 471.06193, found [M + H]^+^: 471.06151.

##### 4.2.2.7 (*E*)-*N*-Benzyl-2-{[1-(thiophen-2-ylsulfonyl)-1*H*-indol-3-yl]methylene}hydrazine-1-carbothioamide (5g)

Brown; m.p.: 220–222 °C; yield: 79%; ^1^H NMR (400 MHz, CDCl_3_) *δ* 9.85 (s, 1H, NH), 8.04 (s, 1H, NH), 8.01 (d, *J* = 8.4 Hz, 1H, CH), 7.87 (d, *J* = 7.9 Hz, 1H, Ar–H), 7.81 (s, 1H, Ar–H), 7.73 (dd, *J* = 3.8, 1.4 Hz, 1H, Ar–H), 7.64–7.55 (m, 2H, Ar–H), 7.42 (dd, *J* = 7.7, 3.0 Hz, 3H, Ar–H), 7.38 (d, *J* = 7.4 Hz, 2H, Ar–H), 7.35–7.30 (m, 1H, Ar–H), 7.23 (d, *J* = 7.7 Hz, 1H, Ar–H), 7.02 (t, *J* = 4.4 Hz, 1H, Ar–H), 4.98 (s, 2H, CH_2_). ^13^C NMR (101 MHz, CDCl_3_) *δ* 177.21 (C=S), 137.31 (HC = N), 137.26 (Ar), 136.69 (Ar), 135.18 (Ar), 134.18 (Ar), 133.70 (Ar), 128.83 (Ar), 128.71 (Ar), 127.66 (Ar), 127.60 (Ar), 127.44 (Ar), 126.79 (Ar), 125.89 (Ar), 124.32 (Ar), 121.99 (Ar), 117.49 (Ar), 113.63 (Ar), 48.29 (CH_2_). ESI-HRMS (*m*/*z*): chemical formula: C_21_H_18_N_4_O_2_S_3_, calculated [M + H]^+^: 455.06701, found [M + H]^+^: 455.06654.

##### 4.2.2.8 (*E*)-*N*-Cyclohexyl-2-{[1-(thiophen-2-ylsulfonyl)-1*H*-indol-3-yl]methylene}hydrazine-1-carbothioamide (5h)

Off-white; mp.: 246–248 °C; yield; 82%; ^1^H NMR (400 MHz, CDCl_3_) *δ* 9.63 (s, 1H), 8.05 (d, *J* = 8.3 Hz, 1H), 8.00 (d, *J* = 10.7 Hz, 2H), 7.82 (s, 1H), 7.75 (d, *J* = 3.7 Hz, 1H), 7.60 (d, *J* = 4.9 Hz, 1H), 7.45 (t, *J* = 7.7 Hz, 1H), 7.38 (t, *J* = 7.6 Hz, 1H), 7.04 (t, *J* = 4.4 Hz, 1H), 4.36–4.28 (m, 1H), 2.13 (dd, *J* = 12.3, 3.1 Hz, 2H), 1.77 (dd, *J* = 9.2, 4.1 Hz, 2H), 1.69–1.61 (m, 2H), 1.50 (d, *J* = 11.0 Hz, 2H), 1.39 (dd, *J* = 17.1, 7.4 Hz, 2H), 1.34–1.29 (m, 1H). ^13^C NMR (101 MHz, CDCl_3_) *δ* 175.89 (C=S), 137.66 (HC = N), 136.40 (Ar), 135.58 (Ar), 134.49 (Ar), 134.00 (Ar), 128.97 (Ar), 127.92 (Ar), 127.25 (Ar), 126.22 (Ar), 124.66 (Ar), 122.22 (Ar), 118.00 (Ar), 114.05 (Ar), 52.90 (cyclohexyl), 32.81 (cyclohexyl), 25.65 (cyclohexyl), 24.70 (cyclohexyl). ESI-HRMS (*m*/*z*): chemical formula: C_20_H_22_N_4_O_2_S_3_, calculated [M + H]^+^: 447.09831, found [M + H]^+^: 447.09752.

##### 4.2.2.9 (*E*)-*N*-(2-Chlorophenyl)-2-{[1-(thiophen-2-ylsulfonyl)-1*H*-indol-3-yl]methylene}hydrazine-1-carbothioamide (5i)

Gray; m.p.: 223–225 °C; yield: 76%; ^1^H NMR (400 MHz, CDCl_3_) *δ* 9.68 (s, 1H, NH), 9.49 (s, 1H, NH), 8.78 (d, *J* = 7.4 Hz, 1H, CH), 8.30 (d, *J* = 6.2 Hz, 1H, Ar–H), 8.06 (s, 2H, Ar–H), 7.87 (s, 1H, Ar–H), 7.77 (s, 1H, Ar–H), 7.61 (s, 1H, Ar–H), 7.46 (s, 2H, Ar–H), 7.37 (s, 2H, Ar–H), 7.17 (s, 1H, Ar–H), 7.05 (s, 1H, Ar–H). ^13^C NMR (101 MHz, DMSO) *δ* 175.77 (C=S), 138.58 (HC = N), 136.97 (Ar), 136.28 (Ar), 135.87 (Ar), 135.27 (Ar), 134.63 (Ar), 130.29 (Ar), 129.35 (Ar), 129.26 (Ar), 128.79 (Ar), 128.60 (Ar), 127.39 (Ar), 127.09 (Ar), 126.68 (Ar), 126.04 (Ar), 124.49 (Ar), 123.14 (Ar), 117.98 (Ar), 113.20 (Ar). ESI-HRMS (*m*/*z*): chemical formula: C_20_H_14_ClN_4_O_2_S_3_, calculated [M − H]^+^: 472.99674, found [M + H]^+^: 472.99792.

##### 4.2.2.10 (*E*)-*N*-(4-Fluorophenyl)-2-{[1-(thiophen-2-ylsulfonyl)-1*H*-indol-3-yl]methylene}hydrazine-1-carbothioamide (5j)

Off-white; m.p.: 234–236 °C; yield: 85%; ^1^H NMR (400 MHz, CDCl_3_) *δ* 9.74 (s, 1H, NH), 8.97 (s, 1H, NH), 8.09–8.02 (m, 3H, Ar–H), 7.88 (s, 1H, CH), 7.80–7.75 (m, 1H, Ar–H), 7.61 (dd, *J* = 8.6, 4.8 Hz, 3H, Ar–H), 7.46 (t, *J* = 8.0 Hz, 1H, Ar–H), 7.39 (t, *J* = 7.5 Hz, 1H, Ar–H), 7.13 (t, *J* = 8.6 Hz, 2H, Ar–H), 7.05 (t, *J* = 4.4 Hz, 1H, Ar–H). ^13^C NMR (101 MHz, CDCl_3_) *δ* 176.77 (C=S), 138.64 (HC = N), 137.98 (Ar), 137.23 (Ar), 136.04 (Ar), 135.13 (Ar), 134.64 (Ar), 130.11 (Ar), 128.48 (Ar), 127.53 (Ar), 127.44 (Ar), 126.87 (Ar), 125.31 (Ar), 122.69 (Ar), 117.95 (Ar), 116.50 (Ar), 116.27 (Ar), 114.56 (Ar). ^19^F NMR (471 MHz, DMSO) *δ* −117.04. ESI-HRMS (*m*/*z*): chemical formula: C_20_H_15_FN_4_O_2_S_3_, calculated [M + H]^+^: 459.04194, found [M + H]^+^: 459.04065.

##### 4.2.2.11 (*E*)-2-{[1-(Thiophen-2-ylsulfonyl)-1*H*-indol-3-yl]methylene}-*N*-(*o*-tolyl)hydrazine-1-carbothioamide (5k)

Off-white; m.p.: 226–228 °C; yield: 80%; ^1^H NMR (400 MHz, CDCl_3_) *δ* 10.15 (s, 1H, NH), 8.85 (s, 1H, NH), 8.13 (s, 1H, CH), 8.06 (t, *J* = 8.8 Hz, 2H, Ar–H), 7.86 (s, 1H, Ar–H), 7.78–7.73 (m, 2H, Ar–H), 7.61 (d, *J* = 4.0 Hz, 1H, Ar–H), 7.45 (t, *J* = 7.6 Hz, 1H, Ar–H), 7.36 (d, *J* = 7.8 Hz, 1H, Ar–H), 7.33–7.28 (m, 3H, Ar–H), 7.06–7.03 (m, 1H, Ar–H), 2.39 (s, 3H, CH_3_). ^13^C NMR (101 MHz, CDCl_3_) *δ* 176.42 (C=S), 137.55 (HC = N), 137.47 (Ar), 136.38 (Ar), 135.48 (Ar), 134.49 (Ar), 134.00 (Ar), 133.89 (Ar), 130.81 (Ar), 129.66 (Ar), 127.87 (Ar), 127.48 (Ar), 127.32 (Ar), 126.96 (Ar), 126.63 (Ar), 126.24 (Ar), 124.64 (Ar), 122.32 (Ar), 117.62 (Ar), 113.94 (Ar), 18.12 (CH_3_). ESI-HRMS (*m*/*z*): chemical formula: C_21_H_18_N_4_O_2_S_3_, calculated [M + H]^+^: 455.06701, found [M + H]^+^: 455.06616.

##### 4.2.2.12 (*E*)-*N*-Phenyl-2-{[1-(thiophen-2-ylsulfonyl)-1*H*-indol-3-yl]methylene}hydrazine-1-carbothioamide (5l)

Brown; m.p.: 228–230 °C; yield: 83%; ^1^H NMR (400 MHz, DMSO) *δ* 11.87 (s, 1H, NH), 9.82 (s, 1H, NH), 8.40 (s, 2H, Ar–H), 8.28 (d, *J* = 7.9 Hz, 1H, CH), 8.08 (dd, *J* = 5.0, 1.4 Hz, 1H, Ar–H), 8.02 (dd, *J* = 3.9, 1.4 Hz, 1H, Ar–H), 7.98 (d, *J* = 8.3 Hz, 1H, Ar–H), 7.62–7.55 (m, 2H, Ar–H), 7.51–7.43 (m, 1H, Ar–H), 7.38 (td, *J* = 8.0, 4.1 Hz, 3H, Ar–H), 7.26–7.17 (m, 2H, Ar–H). ^13^C NMR (101 MHz, DMSO) *δ* 175.84 (C=S), 139.21(HC = N), 137.85 (Ar), 136.90 (Ar), 135.91 (Ar), 135.20 (Ar), 134.62 (Ar), 129.49 (Ar), 128.58 (Ar), 128.17 (Ar), 126.97 (Ar), 125.96 (Ar), 125.76 (Ar), 125.35 (Ar), 124.59 (Ar), 122.99 (Ar), 118.22 (Ar), 113.18 (Ar). ESI-HRMS (*m*/*z*): chemical formula: C_20_H_16_N_4_O_2_S_3_, calculated [M + H]^+^: 441.05136, found [M + H]^+^: 441.05048.

##### 4.2.2.13 (*E*)-*N*-(3-Chlorophenyl)-2-{[1-(thiophen-2-ylsulfonyl)-1*H*-indol-3-yl]methylene}hydrazine-1-carbothioamide (5m)

Light-brown; m.p.: 211–213 °C; yield: 85%; ^1^H NMR (400 MHz, DMSO) *δ* 11.99 (s, 1H, NH), 9.89 (s, 1H, NH), 8.40 (s, 2H, Ar–H), 8.27 (d, *J* = 7.9 Hz, 1H, CH), 8.08 (d, *J* = 5.0 Hz, 1H, Ar–H), 8.02 (d, *J* = 3.6 Hz, 1H, Ar–H), 7.97 (d, *J* = 8.3 Hz, 1H, Ar–H), 7.77 (d, *J* = 2.2 Hz, 1H, Ar–H), 7.54 (s, 1H, Ar–H), 7.47 (s, 1H, Ar–H), 7.43–7.34 (m, 2H, Ar–H), 7.27 (s, 1H, Ar–H), 7.21 (d, *J* = 4.4 Hz, 1H, Ar–H). ^13^C NMR (101 MHz, DMSO) *δ* 176.20 (C=S), 141.20 (HC = N), 138.85 (Ar), 137.67 (Ar), 137.39 (Ar), 136.33 (Ar), 135.68 (Ar), 135.06 (Ar), 132.66 (Ar), 130.14 (Ar), 129.03 (Ar), 127.37 (Ar), 126.43 (Ar), 125.61 (Ar), 125.46 (Ar), 125.04 (Ar), 124.75 (Ar), 123.51 (Ar), 118.58 (Ar), 113.61 (Ar). ESI-HRMS (*m*/*z*): chemical formula: C_20_H_15_ClN_4_O_2_S_3_, calculated [M + H]^+^: 475.01239, found [M + H]^+^: 475.01181.

##### 4.2.2.14 (*E*)-2-{[1-(Thiophen-2-ylsulfonyl)-1*H*-indol-3-yl]methylene}hydrazine-1-carbothioamide (5n)

Brown; m.p.: 232–234 °C; yield: 78%; ^1^H NMR (400 MHz, DMSO) *δ* 11.48 (s, 1H, NH), 8.31 (d, *J* = 7.9 Hz, 1H, NH), 8.28 (s, 2H, NH_2_), 8.25 (s, 1H, CH), 8.06 (dd, *J* = 5.0, 1.3 Hz, 1H, Ar–H), 7.99 (dd, *J* = 3.9, 1.3 Hz, 1H, Ar–H), 7.95 (d, *J* = 8.3 Hz, 1H, Ar–H), 7.65 (s, 1H, Ar–H), 7.48–7.43 (m, 1H, Ar–H), 7.38–7.33 (m, 1H, Ar–H), 7.18 (dd, *J* = 4.9, 4.0 Hz, 1H, Ar–H). ^13^C NMR (101 MHz, DMSO) *δ* 177.61 (C=S), 137.66 (HC = N), 136.82 (Ar), 135.94 (Ar), 135.14(Ar), 134.64 (Ar), 129.64 (Ar), 128.56 (Ar), 126.77 (Ar), 125.94 (Ar), 124.62 (Ar), 123.49 (Ar), 118.33 (Ar), 113.07 (Ar). ESI-HRMS (*m*/*z*): chemical formula: C_14_H_12_N_4_O_2_S_3_, calculated [M + H]^+^: 365.02006, found [M + H]^+^: 365.01928.

##### 4.2.2.15 (*E*)-*N*-Isopropyl-2-{[1-(thiophen-2-ylsulfonyl)-1*H*-indol-3-yl]methylene}hydrazine-1-carbothioamide (5o)

Light-brown; mp.: 242–244 °C; yield: 82%; ^1^H NMR (400 MHz, CDCl_3_) *δ* 9.89 (s, 1H, NH), 8.06 (s, 1H, CH), 8.03 (s, 1H, CH), 7.98 (d, *J* = 7.8 Hz, 1H, Ar–H), 7.83 (s, 1H, Ar–H), 7.75 (d, *J* = 3.1 Hz, 1H, Ar–H), 7.59 (d, *J* = 4.9 Hz, 1H, Ar–H), 7.45 (t, *J* = 7.7 Hz, 1H, Ar–H), 7.38 (t, *J* = 7.5 Hz, 1H, Ar–H), 7.14 (d, *J* = 8.2 Hz, 1H, Ar–H), 7.03 (t, *J* = 4.4 Hz, 1H, Ar–H), 4.62 (dq, *J* = 13.3, 6.6 Hz, 1H, CH), 1.36 (d, *J* = 6.5 Hz, 6H [CH_3_]_2_). ^13^C NMR (101 MHz, CDCl_3_) *δ* 175.94 (C=S), 137.64 (HC = N), 136.60 (Ar), 135.55 (Ar), 134.48 (Ar), 134.00 (Ar), 128.93 (Ar), 127.92 (Ar), 127.26 (Ar), 126.20 (Ar), 124.66 (Ar), 122.12 (Ar), 117.97 (Ar), 114.03 (Ar), 46.52 (CH-isopropyl), 22.79 (CH_3_-isopropyl). ESI-HRMS (*m*/*z*): chemical formula: C_17_H_18_N_4_O_2_S_3_, calculated [M + H]^+^: 407.06701, found [M + H]^+^: 407.06613.

##### 4.2.2.16 (*E*)-*N*-(*Tert*-butyl)-2-{[1-(thiophen-2-ylsulfonyl)-1*H*-indol-3-yl]methylene}hydrazine-1-carbothioamide (5p)

Brown; m.p.: 232–234 °C; yield: 85%; ^1^H NMR (400 MHz, CDCl_3_) *δ* 9.74 (s, 1H, NH), 8.05 (s, 1H, NH), 8.03 (s, 1H, CH), 8.00 (d, *J* = 8.0 Hz, 1H, Ar–H), 7.80 (s, 1H, Ar–H), 7.74 (d, *J* = 3.5 Hz, 1H, Ar–H), 7.59 (d, *J* = 4.9 Hz, 1H, Ar–H), 7.47 (d, *J* = 5.6 Hz, 1H, Ar–H), 7.43 (d, *J* = 7.8 Hz, 1H, Ar–H), 7.35 (t, *J* = 7.6 Hz, 1H, Ar–H, Ar–H), 7.03 (t, *J* = 4.4 Hz, 1H, Ar–H), 1.64 (s, 9H, [CH_3_]_3_). ^13^C NMR (101 MHz, CDCl_3_) *δ* 175.53 (C=S), 137.64 (HC = N), 135.61 (Ar), 134.44 (Ar), 133.96 (Ar), 128.97 (Ar), 127.90 (Ar), 127.20 (Ar), 126.17 (Ar), 124.58 (Ar), 122.29 (Ar), 118.17 (Ar), 114.03 (Ar), 53.77 (*tert*-butyl), 29.11 (CH_3_-*tert*-butyl). ESI-HRMS (*m*/*z*): chemical formula: C_18_H_20_N_4_O_2_S_3_, calculated [M + H]^+^: 421.08266, found [M + H]^+^: 421.08141.

##### 4.2.2.17 (*E*)-*N*-(4-Fluorobenzyl)-2-{[1-(thiophen-2-ylsulfonyl)-1*H*-indol-3-yl]methylene}vhydrazine-1-carbothioamide (5q)

Brown; m.p.: 215–217 °C; yield: 81%; ^1^H NMR (400 MHz, DMSO) *δ* 11.66 (s, 1H, NH), 8.69 (t, *J* = 6.0 Hz, 1H, Ar–H), 8.34 (s, 1H, NH), 8.31 (s, 1H, CH), 8.28 (d, *J* = 7.9 Hz, 1H, Ar–H), 8.07 (d, *J* = 4.2 Hz, 1H, Ar–H), 8.00 (d, *J* = 2.9 Hz, 1H, Ar–H), 7.97 (d, *J* = 8.3 Hz, 1H, Ar–H), 7.47 (t, *J* = 7.7 Hz, 1H, Ar–H), 7.38 (dt, *J* = 14.9, 6.7 Hz, 3H, Ar–H), 7.21–7.13 (m, 3H, Ar–H), 4.87 (d, *J* = 6.0 Hz, 2H, CH_2_). ^13^C NMR (101 MHz, DMSO) *δ* 177.46 (C=S), 162.43 (HC = N), 160.03 (Ar), 137.79 (Ar), 136.95 (Ar), 135.99 (Ar), 135.71 (Ar), 135.69 (Ar), 135.26 (Ar), 134.73 (Ar), 129.54 (Ar), 129.17 (Ar), 129.09 (Ar), 128.64 (Ar), 126.92 (Ar), 126.05 (Ar), 124.63 (Ar), 123.26 (Ar), 118.37 (Ar), 115.11 (Ar), 114.90 (Ar), 113.24 (Ar), 46.14 (CH_2_). C–F Coupling: 161.23 (d, ^1^*J* = 242.0 Hz; 162.43, 160.03), 135.70 (d, ^4^*J* = 2.9 Hz; 135.7, 135.6), 129.13 (d, ^3^*J* = 8.1 Hz; 129.17, 128.99), 115.00 (d, ^2^*J* = 21.3 Hz; 115.11, 114.90). ^19^F NMR (471 MHz, DMSO) *δ* −116.46. ESI-HRMS (*m*/*z*): chemical formula: C_21_H_16_FN_4_O_2_S_3_, calculated [M − H]^+^: 471.04194, found [M − H]^+^: 471.04241.

##### 4.2.2.18 (*E*)-*N*-(3-Fluorophenyl)-2-{[1-(thiophen-2-ylsulfonyl)-1*H*-indol-3-yl]methylene}hydrazine-1-carbothioamide (5r)

Off-white; m.p.: 200–202 °C; yield: 82%; ^1^H NMR (400 MHz, CDCl_3_) *δ* 9.56 (s, 1H, NH), 9.13 (s, 1H, NH), 8.07 (s, 1H, CH), 8.05 (s, 1H, Ar–H), 8.04 (s, 1H, Ar–H), 7.89 (s, 1H, Ar–H), 7.77 (d, *J* = 3.4 Hz, 1H, Ar–H), 7.71 (d, *J* = 10.4 Hz, 1H, Ar–H), 7.62 (d, *J* = 4.9 Hz, 1H, Ar–H), 7.47 (t, *J* = 7.7 Hz, 1H, Ar–H), 7.40 (dd, *J* = 16.2, 9.2 Hz, 3H, Ar–H), 7.05 (t, *J* = 4.4 Hz, 1H, Ar–H), 6.97 (t, *J* = 7.5 Hz, 1H, Ar–H). ^13^C NMR (101 MHz, CDCl_3_) *δ* 175.07 (C=S), 137.28 (HC = N), 137.04 (Ar), 135.26 (Ar), 134.36 (Ar), 133.86 (Ar), 129.86 (Ar), 129.77 (Ar), 129.40 (Ar), 127.69 (Ar), 126.70 (Ar), 126.12 (Ar), 124.57 (Ar), 121.83 (Ar), 119.10 (Ar), 117.06 (Ar), 113.79 (Ar), 112.88 (Ar), 112.78 (Ar). ^19^F NMR (471 MHz, DMSO) *δ* −113.15. ESI-HRMS (*m*/*z*): chemical formula: C_20_H_15_FN_4_O_2_S_3_, calculated [M + H]^+^: 459.04194, found [M + H]^+^: 459.04108.

##### 4.2.2.19 (*E*)-*N*-Methyl-2-{[1-(thiophen-2-ylsulfonyl)-1*H*-indol-3-yl]methylene}hydrazine-1-carbothioamide (5s)

Brown; m.p.: 231–233 °C; yield: 84%; ^1^H NMR (400 MHz, CDCl_3_) *δ* 9.43 (s, 1H), 8.03 (t, *J* = 8.4 Hz, 2H, Ar–H), 7.97 (s, 1H, NH), 7.83 (s, 1H), 7.75 (d, *J* = 3.7 Hz, 1H), 7.60 (d, *J* = 4.9 Hz, 1H), 7.44 (d, *J* = 8.0 Hz, 1H), 7.39 (d, *J* = 7.7 Hz, 1H, CH), 7.31 (s, 1H, Ar–H), 7.04 (t, *J* = 4.4 Hz, 1H, Ar–H), 3.31 (d, *J* = 4.7 Hz, 3H, CH_3_). ^13^C NMR (101 MHz, CDCl_3_) *δ* 178.22 (C=S), 136.55 (HC = N), 134.36 (Ar), 134.34 (Ar), 133.96 (Ar), 128.82 (Ar), 127.76 (Ar), 127.00 (Ar), 126.09 (Ar), 124.48 (Ar), 122.13 (Ar), 119.92 (Ar), 113.80 (Ar), 31.39 (CH_3_). ESI-HRMS (*m*/*z*): chemical formula: C_15_H_14_N_4_O_2_S_3_, calculated [M + H]^+^: 379.03571, found [M + H]^+^: 379.03495.

##### 4.2.2.20 (*E*)-*N*-(4-Bromophenyl)-2-{[1-(thiophen-2-ylsulfonyl)-1*H*-indol-3-yl]methylene}hydrazine-1-carbothioamide (5t)

Off-white; m.p.: 226–228 °C; yield: 85%; ^1^H NMR (400 MHz, CDCl_3_) *δ* 9.36 (s, 1H, NH), 9.03 (s, 1H, NH), 8.07 (s, 1H, CH), 8.05 (s, 1H, Ar–H), 8.02 (s, 2H, Ar–H), 7.89 (s, 1H, Ar–H), 7.77 (d, *J* = 3.7 Hz, 1H, Ar–H), 7.61 (d, *J* = 8.8 Hz, 2H, Ar–H), 7.54 (d, *J* = 8.7 Hz, 2H, Ar–H), 7.47 (t, *J* = 7.7 Hz, 1H, Ar–H), 7.40 (t, *J* = 7.6 Hz, 1H, Ar–H), 7.06 (t, *J* = 4.4 Hz, 1H, Ar–H). ^13^C NMR (101 MHz, DMSO) *δ* 175.76 (C=S), 138.66 (HC = N), 138.22 (Ar), 136.94 (Ar), 135.88 (Ar), 135.22 (Ar), 134.60 (Ar), 130.95 (Ar), 129.61 (Ar), 128.57 (Ar), 127.72 (Ar), 126.93 (Ar), 125.96 (Ar), 124.57 (Ar), 123.03 (Ar), 118.14 (Ar), 117.53 (Ar), 113.16 (Ar). ESI-HRMS (*m*/*z*): chemical formula: C_20_H_15_BrN_4_O_2_S_3_, calculated [M + H]^+^: 518.96187, found [M + H]^+^: 518.96118.

### 4.3 Biological activity

Human lung adenocarcinoma A549 (American Type Culture Collection (ATCC)^®^ CCL-185™), human prostate adenocarcinoma LNCaP (ATCC^®^ CRL-1740™), and human umbilical vein endothelial HUVEC (ATCC^®^ CRL-1730™) cells were obtained from ATCC (VA, USA). Cells were cultured in DMEM/F12 medium (Gibco, MA, USA) supplemented with 10% fetal bovine serum (FBS), 100 000 U L^−1^ penicillin, and 100 000 µg L^−1^ streptomycin. Cell cultures were maintained at 37 °C in a humidified atmosphere containing 5% CO_2_ and routinely monitored for appropriate morphology and confluence.^56^

The cytotoxic effects of the synthesized compounds were evaluated using the MTT assay. Briefly, A549, LNCaP, and HUVEC cells were seeded into 96-well plates at 5000 cells per well and allowed to attach for 24 h under standard culture conditions. Then, cells were treated with serial concentrations of the synthesized compounds, with the highest tested concentration being 200 µM and incubated for an additional 24 h. Untreated cells were used as the control group. Following treatment, 5 mg mL^−1^ MTT solution (Thermo Fisher Scientific, MA, USA) was added to each well, and the plates were incubated for 3 h at 37 °C. The medium was subsequently removed, and 100 µL of DMSO (ISOLAB, Germany) was added to dissolve the formed formazan crystals. Absorbance was measured at 540 nm using a BioTek Synergy H1 ELISA reader.^57^

Cell viability was expressed as a percentage relative to untreated control cells. IC_50_ values were calculated from concentration–response curves using Prism 9.0 (GraphPad, CA, USA). All experiments were performed in triplicate, and the results are expressed as mean ± SD. The SI was calculated as the ratio of the IC_50_ value obtained in HUVEC cells to that obtained in the corresponding cancer cell line.

### 4.4 Computational studies

Molecular docking, MM-GBSA, MD simulations, ADME, and DFT calculations were performed using Schrödinger Suite (Release 2025-1) through the Maestro interface (version 14.3), with Desmond used for MD simulations. The crystal structures of AR (2PNU-Chain A), EGFR (3W2S Chain A), VEGFR-2 (4ASE Chain A), AKT1 (4EJN Chain A), mTOR (4JT5 Chain A), and PI3Kα/P110α (4L23 Chain A) were obtained from the Protein Data Bank and prepared using the Protein Preparation Wizard. Missing hydrogens were added, bond orders were assigned, and hydrogen-bonding networks were optimized using default settings. Synthesized compounds were prepared using the LigPrep module. For molecular docking, the Glide XP protocol was initially applied using receptor grids centered on the corresponding co-crystallized ligands with a 15 Å grid box. Up to 20 poses were generated for each ligand, and the best-ranked poses were subsequently subjected to IFD. The resulting complexes were further evaluated using Prime MM-GBSA with the VSGB solvation model.^58^ The docking protocol was validated by redocking the co-crystallized native ligands into their respective binding sites. MM-GBSA energy decomposition analysis was additionally performed to characterize the energetic contributions of individual interaction components for the selected AR complexes. MD simulations were performed for the AR–compound 5d complex using Desmond (Release 2024-3) with the OPLS4 force field and explicit TIP4P water. The system was neutralized and supplemented with 0.15 M NaCl, followed by equilibration under NVT and NPT ensembles. A 250 ns production simulation was performed at 300 K and 1.01325 bar. The Nosé–Hoover thermostat and Martyna–Tobias–Klein barostat were used for temperature and pressure control, respectively. The dynamic stability of the complex was assessed primarily through RMSD and RMSF analyses.^59^

The ADME properties of compounds 5b–5d and sorafenib were predicted using QikProp in Schrödinger Suite 2025-1. Molecular weight, hydrogen-bond acceptor and donor counts, QP log *P*_o/w_, QP log *S*, QP log BB, QPPCaco, QPPMDCK, % human oral absorption, Lipinski's Rule of Five and Jorgensen's Rule of Three were evaluated. DFT calculations were performed using the Jaguar module at the B3LYP/6-31G** level. The HOMO and LUMO energies, HOMO–LUMO energy gap, molecular electrostatic potential (MEP), and global chemical reactivity descriptors, including ionization potential, electron affinity, electronegativity, chemical potential, global hardness, global softness, and electrophilicity index, were calculated from the optimized structures.^60^

## Author contributions

Kazma Batool and Metab Alharbi: synthesis of new compounds and purification, taking physical data of new compounds. Halil Şenol and Ayşe Merve Şenol: performed computational studies; molecular docking, MD simulation studies, ADMET studies, created figures, wrote the Discussion section and original draft of the manuscript. Asif Rasool and Abdullah F. Alasmari: literature survey, SAR discussion, performed NMR spectroscopy and interpreted the data, provided resources for analyses and funding to purchase chemicals. Hande İpek İnce and Fatma Ateş: performed cytotoxicity experiments, created graphs, wrote the Discussion section. Zahid Shafiq and Abdulrahman Alshammari: supervision of doctorals student and project, conceptualization, writing and reviewing the original and revised draft, resources and laboratory facilities.

## Conflicts of interest

The authors declare that they have no known competing financial interests or personal relationships that could have appeared to influence the work reported in this paper.

## Supplementary Material

RA-OLF-D6RA08602K-s001

## Data Availability

All data generated or analyzed during this study are included in this published article and its supplementary information (SI) files. Supplementary information is available. See DOI: https://doi.org/10.1039/d6ra08602k.

## References

[cit1] A. C. Society , Cancer Facts & Figures 2025, 2025, https://www.cancer.org/research/cancer-facts-statistics/allcancer-facts-figures/2025-cancer-facts-figures.html

[cit2] Corn B. W., Feldman D. B. (2025). Cancer statistics, 2025: A hinge moment for optimism to morph into hope?. Ca-Cancer J. Clin..

[cit3] Siegel R. L., Kratzer T. B., Giaquinto A. N., Sung H., Jemal A. (2025). Cancer statistics, 2025. Ca-Cancer J. Clin..

[cit4] Kratzer T. B., Bandi P., Freedman N. D., Smith R. A., Travis W. D., Jemal A., Siegel R. L. (2024). Lung cancer statistics, 2023. Cancer.

[cit5] Miller K. D., Nogueira L., Mariotto A. B., Rowland J. H., Yabroff K. R., Alfano C. M., Jemal A., Kramer J. L., Siegel R. L. (2019). Cancer treatment and survivorship statistics, 2019. Ca-Cancer J. Clin..

[cit6] Verma N., Arora S. (2025). Navigating the Global Regulatory Landscape for Exosome-Based Therapeutics: Challenges, Strategies, and Future Directions. Pharmaceutics.

[cit7] Verma N., Arora S., Croghan K. (2026). Tiny Messengers, Actionable Targets: sEV-Driven Epigenetic Changes in Cancer. Targets.

[cit8] Verma N., Arora S. (2026). Regulatory Convergence in Cell and Gene Therapy: Harmonizing Quality, CMC, and Approval Pathways Across the FDA, EMA, PMDA, and Emerging Markets. BioTech.

[cit9] Bray F., Laversanne M., Sung H., Ferlay J., Siegel R. L., Soerjomataram I., Jemal A. (2024). Global cancer statistics 2022: GLOBOCAN estimates of incidence and mortality worldwide for 36 cancers in 185 countries. Ca-Cancer J. Clin..

[cit10] Gamal H., Ismail K. A., Omar A., Teleb M., Abu-Serie M. M., Huang S., Abdelsattar A. S., Zamponi G. W., Fahmy H. (2024). Non-small cell lung cancer sensitisation to platinum chemotherapy *via* new thiazole-triazole hybrids acting as dual T-type CCB/MMP-9 inhibitors. J. Enzyme Inhib. Med. Chem..

[cit11] Acar Ö. Ö., Gazioğlu I., Oruç H., Kale E., Şenol H., Topçu G., Şen A. (2026). A Small Indole Derivative Isolated From Caper (Capparis ovata) as an Inducer of P53-Mediated Apoptosis in Prostate Cancer: Comprehensive *In Vitro* and *In Silico* Studies. J. Biochem. Mol. Toxicol..

[cit12] Wang Y., Chen J., Kuang J., Wang X. (2026). Development and Validation of A Nomogram for Prostate Cancer Diagnosis Based on Inflammatory Ratios and PSA Derived Indicators. Arch. Esp. Urol..

[cit13] Şenol H., Ghaffari-Moghaddam M., Bulut Ş., Akbaş F., Köse A., Topçu G. (2023). Synthesis and Anticancer Activity of Novel Derivatives of α,β-Unsaturated Ketones Based on Oleanolic Acid: *in Vitro* and *in Silico* Studies against Prostate Cancer Cells. Chem. Biodiversity.

[cit14] Hacıosmanoğlu-Aldoğan E., Lama D., Yetke H., Şenol H., Yöntem F. D. (2025). Necroptotic Suppression of Lung Cancer Cell Proliferation and Migration: A Comprehensive *In Vitro* and *In Silico* Study to Determine New Molecular Targets for Pexidartinib. Cell Biochem. Funct..

[cit15] Zafar A., Khatoon S., Khan M. J., Abu J., Naeem A. (2025). Advancements and limitations in traditional anti-cancer therapies: a comprehensive review of surgery, chemotherapy, radiation therapy, and hormonal therapy. Discover Oncol..

[cit16] Tokalı P., Şenol A. M., Ateşoğlu Ş., Çakır F., Şenol H., Tokali F. S., Akbaş F. (2026). New quinazolinone-thiazolidinedione hybrids as selective anti-lung cancer agents and promising EGFR inhibitors. Future Med. Chem..

[cit17] Patil T., Gao D. X., Watson A., Sakamoto M., Nie Y. A., Gibson A., Dean M. L., Yoder B. A., Miller E., Stalker M., Aisner D. L., Bunn P. A., Schenk E. L., Marmarelis M. E., Bennati C., Navani V., Zhang Y. C., Camidge D. R. (2025). The efficacy of continuing osimertinib with platinum pemetrexed chemotherapy upon progression in patients with metastatic non-small cell lung cancer harboring sensitizing EGFR mutations. Lung Cancer.

[cit18] Zengin Kurt B., Özmen Ö., Öztürk Civelek D., Şenol H., Sonmez F. (2025). Exploring anticancer properties of new triazole-linked benzenesulfonamide derivatives against colorectal carcinoma: Synthesis, cytotoxicity, and *in silico* insights. Bioorg. Med. Chem..

[cit19] Wang S., Shi J. T., Wang X. R., Mu H. X., Wang X. T., Xu K. Y., Wang Q. S., Chen S. W. (2023). 1*H*-Indazoles derivatives targeting PI3K/AKT/mTOR pathway: Synthesis, anti-tumor effect and molecular mechanism. Bioorg. Chem..

[cit20] Li C., Han Y., Wang Z., Yu Y., Wang C., Ren Z., Guo Y., Zhu T., Li X., Dong S., Ma M. (2023). Function-oriented synthesis of Imidazo[1,2-*a*]pyrazine and Imidazo[1,2-*b*]pyridazine derivatives as potent PI3K/mTOR dual inhibitors. Eur. J. Med. Chem..

[cit21] Yang Y. Y., Wang W. L., Hu X. T., Chen X., Ni Y., Lei Y. H., Qiu Q. Y., Tao L. Y., Luo T. W., Wang N. Y. (2023). Design, synthesis and biological evaluation of novel 9-methyl-9*H*-purine and thieno[3, 2-*d*]pyrimidine derivatives as potent mTOR inhibitors. Bioorg. Chem..

[cit22] Han Y., Tian Y., Wang R., Fu S., Jiang J., Dong J., Qin M., Hou Y., Zhao Y. (2020). Design, synthesis and biological evaluation of thieno[3,2-*d*]pyrimidine derivatives containing aroyl hydrazone or aryl hydrazide moieties for PI3K and mTOR dual inhibition. Bioorg. Chem..

[cit23] Krishan S., Sahni S., Richardson D. R. (2020). The anti-tumor agent, Dp44mT, promotes nuclear translocation of TFEB *via* inhibition of the AMPK-mTORC1 axis. Biochim. Biophys. Acta, Mol. Basis Dis..

[cit24] Walker L., Duncan R., Adamson B., Kendall H., Brittain N., Luzzi S., Jones D., Chaytor L., Peel S., Crafter C., O'Neill D. J., Gaughan L. (2024). Defining Splicing Factor Requirements for Androgen Receptor Variant Synthesis in Advanced Prostate Cancer. Mol. Cancer Res..

[cit25] Fattahi M. R., Shoja F., Mojaradi M. A., Nazarinezhad P., Dehghanbanadaki H., Arefnezhad R., Rezaei-Tazangi F., Azimi H. (2026). Therapeutic potentials of curcumin in prostate cancer: a comprehensive review of pathways and clinical prospects. Prostate Int..

[cit26] Zhou J., Guo Z., Peng X., Wu B., Meng Q., Lu X., Feng L., Guo T. (2025). Chrysotoxine regulates ferroptosis and the PI3K/AKT/mTOR pathway to prevent cervical cancer. J. Ethnopharmacol..

[cit27] Wang A., Luo X., Meng X., Lu Z., Chen K., Yang Y. (2023). Discovery of a Novel Bifunctional Steroid Analog, YXG-158, as an Androgen Receptor Degrader and CYP17A1 Inhibitor for the Treatment of Enzalutamide-Resistant Prostate Cancer. J. Med. Chem..

[cit28] Crumbaker M., Khoja L., Joshua A. M. (2017). AR Signaling and the PI3K Pathway in Prostate Cancer. Cancers.

[cit29] Batool Z., Toraman G. Ö. A., Çakır F., Topçu G., Taslimi P., Alharthy R. D., Khan A., Al-Harrasi A., Shafiq Z., Şenol H. (2025). Dual targeting of neuroblastoma and cholinesterase by morpholino/pyrrolidino-sulfonyl-indole thiosemicarbazones: Synthesis, characterization, enzyme inhibition, cytotoxicity, docking and dynamics studies. Bioorg. Chem..

[cit30] Zhou Y., Tang Y., Luo Q., Hu Y., Peng W. (2024). Indole-3-Carboxylic Acid Enhanced Anti-cancer Potency of Doxorubicin *via* Induction of Cellular Senescence in Colorectal Cells. Acta Pol. Pharm. – Drug Res..

[cit31] Sousa G. L. S., Nadur N. F., Ferreira L. D. P., Honório T. D., Simon A., Cabral L. M., Santos M. L. M., Andrade B., de Lima E. V., Clarke J. R., Castro R. N., Moura R. O. D., Kümmerle A. E. (2025). Discovery of novel thiosemicarbazone-acridine targeting butyrylcholinesterase with antioxidant, metal complexing and neuroprotector abilities as potential treatment of Alzheimer's disease: *In vitro*, *in vivo*, and *in silico* studies. Eur. J. Med. Chem..

[cit32] Munir I., Aftab H., Farooq A. A., Şenol H., Taslimi P., Sadeghian N., Alharthy R. D., Rasool A., Ghaffar U., Schenone S., Shafiq Z. (2025). Synthesis of 6-ethoxyphenyl 4-fluorobenzenesulfonate-tagged thiosemicarbazones as carbonic anhydrase inhibitors: *In vitro* and *in silico* approach. Bioorg. Med. Chem..

[cit33] Aftab H., Islam M., Şenol H., Batool Z., Ateşoğlu Ş., Çakır F., Akbaş F., Taslimi P., Alanazi A. K., Noreen F., Shafiq Z. (2025). Synthesis, antiproliferative activity targeting lung cancer and *in silico* studies of hydroxypiperidine substituted thiosemicarbazones. Sci. Rep..

[cit34] Tokali F. S., Şenol H., Ateşoğlu Ş., Tokalı P., Akbaş F. (2025). Design, synthesis, and cytotoxic evaluation of new thiosemicarbazone/thiazolidin-4-one derivatives on PC3 cells. Future Med. Chem..

[cit35] Mohammed M. O., Alkubaisi H. M. M., Haj N. Q. (2024). Synthesis, Identification, and anti-cancer Evaluation of Some heterocyclic Chitosan-Thiosemicarbazones compounds and their nickle (II) complexes. Results Chem..

[cit36] Nicolás A., Quero J. G., Barroso M., Gandara Z., Gude L. (2024). DNA Interactions and Biological Activity of 2,9-Disubstituted 1,10-Phenanthroline Thiosemicarbazone-Based Ligands and a 4-Phenylthiazole Derivative. Biology.

[cit37] Demir Y., Türkeş C., Çavuş M. S., Erdoğan M., Muğlu H., Yakan H., Beydemir Ş. (2023). Enzyme inhibition, molecular docking, and density functional theory studies of new thiosemicarbazones incorporating the 4-hydroxy-3,5-dimethoxy benzaldehyde motif. Arch. Pharm..

[cit38] Verma N., Arora S., Singh A. K., Kumar A. (2025). Extracellular Vesicle-Associated miRNAs in Cornea Health and Disease: Diagnostic Potential and Therapeutic Implications. Targets.

[cit39] Tokalı F. S., Acar O. O., Demir Y., Şenol H., Acar B., Çakır F., Sen A. (2026). Targeting cholinergic dysfunction and neuroinflammation through rationally designed Thieno[3,2-*d*]pyrimidine hybrids. Bioorg. Chem..

[cit40] Çakır F., Ateşoğlu Ş., Müderrisoğlu Z. R., Demirel F., Akbaş F., Tokalı F. S., Şenol H. (2025). Targeting Lung Cancer With Carvacrol-Triazole-Arylidene Hydrazide Hybrids: *In Vitro* and *In Silico* Cytotoxicity Assessments. Chem. Biodiversity.

[cit41] Tokalı F. S., Demir Y., Tokalı P., Ateşoğlu Ş., Şenol H. (2025). New Quinazolin-4(3*H*)-One-Thiazolidine-2,4-Dione Hybrids as Dual Inhibitors of α-Glycosidase and Aldose Reductase: The Synthetic, *In Vitro*, and *In Silico* Approaches. J. Biochem. Mol. Toxicol..

[cit42] Benavides M. A., Hagen K. L., Fang W., Du P., Lin S., Moyer M. P., Yang W., Bland K. I., Grizzle W. E., Bosland M. C. (2010). Suppression by L-methionine of cell cycle progression in LNCaP and MCF-7 cells but not benign cells. Anticancer Res..

[cit43] Ogawa T., Shiga K., Hashimoto S., Kobayashi T., Horii A., Furukawa T. (2003). APAF-1-ALT, a novel alternative splicing form of APAF-1, potentially causes impeded ability of undergoing DNA damage-induced apoptosis in the LNCaP human prostate cancer cell line. Biochem. Biophys. Res. Commun..

[cit44] Chang S.-M., Christian W., Wu M.-H., Chen T.-L., Lin Y.-W., Suen C.-S., Pidugu H. B., Detroja D., Shah A., Hwang M.-J. (2017). Novel indolizino [8, 7-*b*] indole hybrids as anti-small cell lung cancer agents: Regioselective modulation of topoisomerase II inhibitory and DNA crosslinking activities. Eur. J. Med. Chem..

[cit45] Song D., Lim S. H., Kim Y., Lee H., Kim T., Lim H., Min D. S., Han G. (2025). Development and evaluation of indole-based phospholipase D inhibitors for lung cancer immunotherapy. J. Med. Chem..

[cit46] Lee Y., Lee S., Lee Y., Song D., Park S.-H., Kim J., Namkung W., Kim I. (2024). Anticancer evaluation of novel benzofuran–indole hybrids as epidermal growth factor receptor inhibitors against non-small-cell lung cancer cells. Pharmaceuticals.

[cit47] Aftab H., Islam M., Şenol H., Batool Z., Ateşoğlu Ş., Çakır F., Akbaş F., Taslimi P., Alanazi A. K., Noreen F. (2025). Synthesis, antiproliferative activity targeting lung cancer and *in silico* studies of hydroxypiperidine substituted thiosemicarbazones. Sci. Rep..

[cit48] Batool Z., Şenol H., Masood R. S., Salduz T., Yoladi F. B., Akbas F., Tokali F. S., Albekairi N. A., Rasool A., Shafiq Z. (2026). Identification of selective N-pyridinsulfonyl indole based thiosemicarbazone derivatives as potential antiproliferative agents against lung cancer cells. RSC Adv..

[cit49] Xu Z., Shi Y., Pan B., Wang H. (2026). Current landscape and therapeutic prospects of indole hybrids for prostate cancer treatment: A mini-review. Eur. J. Med. Chem..

[cit50] Altintop M. D., Sever B., Özdemir A., Kuş G., Oztopcu-Vatan P., Kabadere S., Kaplancikli Z. A. (2016). Synthesis and evaluation of naphthalene-based thiosemicarbazone derivatives as new anticancer agents against LNCaP prostate cancer cells. J. Enzyme Inhib. Med. Chem..

[cit51] He Z. X., Huo J. L., Gong Y. P., An Q., Zhang X., Qiao H., Yang F. F., Zhang X. H., Jiao L. M., Liu H. M., Ma L. Y., Zhao W. (2021). Design, synthesis and biological evaluation of novel thiosemicarbazone-indole derivatives targeting prostate cancer cells. Eur. J. Med. Chem..

[cit52] Jebamani J., Shivalingappa J., Pranesh S., Pasha M., Pawar C. (2025). Molecular docking, ADME properties and synthesis of thiophene sulfonamide derivatives. Drug Chem. Toxicol..

[cit53] Anisree S., Shanmugavelan P., Palanisamy S., Kishore R., Srivastava N. (2024). Synthesis and anticancer screening of substituted (phenyl)-1-((naphthalen-5-yl) methylene) thiosemicarbazide against PC3 prostate cancer cells. J. Indian Chem. Soc..

[cit54] Arora S., Mao C. (2024). Light-regulated RNA interference induced by *p*-hydroxyphenacyl-modified siRNA in mammalian cells. Nucleosides, Nucleotides Nucleic Acids.

[cit55] Batool Z., Ullah S., Khan A., Mali S. N., Gurav S. S., Jawarkar R. D., Alshammari A., Albekairi N. A., Al-Harrasi A., Shafiq Z. (2024). Design, synthesis, QSAR modelling and molecular dynamic simulations of N-tosyl-indole hybrid thiosemicarbazones as competitive tyrosinase inhibitors. Sci. Rep..

[cit56] Kassi E., Papoutsi Z., Pratsinis H., Aligiannis N., Manoussakis M., Moutsatsou P. (2007). Ursolic acid, a naturally occurring triterpenoid, demonstrates anticancer activity on human prostate cancer cells. J. Cancer Res. Clin. Oncol..

[cit57] Tokalı F. S., Şenol H., Yetke H. I., Hacıosmanoğlu-Aldogan E. (2023). Novel quinazoline-chromene hybrids as anticancer agents: Synthesis, biological activity, molecular docking, dynamics and ADME studies. Arch. Pharm..

[cit58] Zhu Y., Xie D. Y. (2020). Docking Characterization and *in vitro* Inhibitory Activity of Flavan-3-ols and Dimeric Proanthocyanidins Against the Main Protease Activity of SARS-Cov-2. Front. Plant Sci..

[cit59] Fatima A., Ghaffar U., Agrawal M., Sadeghian N., Taslimi P., Ismail M. A., Alharthy R. D., Zhao X., Zaki M. E. A., Gomha S. M., Şenol H., Shafiq Z. (2026). Multi-Target Vanillin-Derived Hydrazones: Cholinesterase and MAO-A Inhibition, Antioxidant Activity and Molecular Modeling toward Alzheimer's Disease. J. Mol. Struct..

[cit60] Çelik Turgut G., Pepe N. A., Ekiz Y. C., Şenol H., Şen A. (2025). Therapeutic Potential of Nitrogen-Substituted Oleanolic Acid Derivatives in Neuroinflammatory and Cytokine Pathways: Insights From Cell-Based and Computational Models. Chem. Biodiversity.

