# Supplementary material for: Thiophen-2-ylsulfonyl indole-3-carbaldehyde thiosemicarbazones as selective prostate cancer agents: biological profiling and molecular targeting studies
Source: RSC Adv. 2026 Nov 9. Online ahead of print. doi: 10.1039/d6ra08602k (PMC13746118; doi:10.1039/d6ra08602k)

Supplementary Information (SI) for RSC Advances.
This journal is © The Royal Society of Chemistry

Contents:

- ^{1}H & ^{13}C NMR Spectra of 5(a-t)
- HRMS of 5(a-t)
- IC_{50} Graphics of 5(a-t)

^{1}H & ^{13}C NMR Spectra of 5a

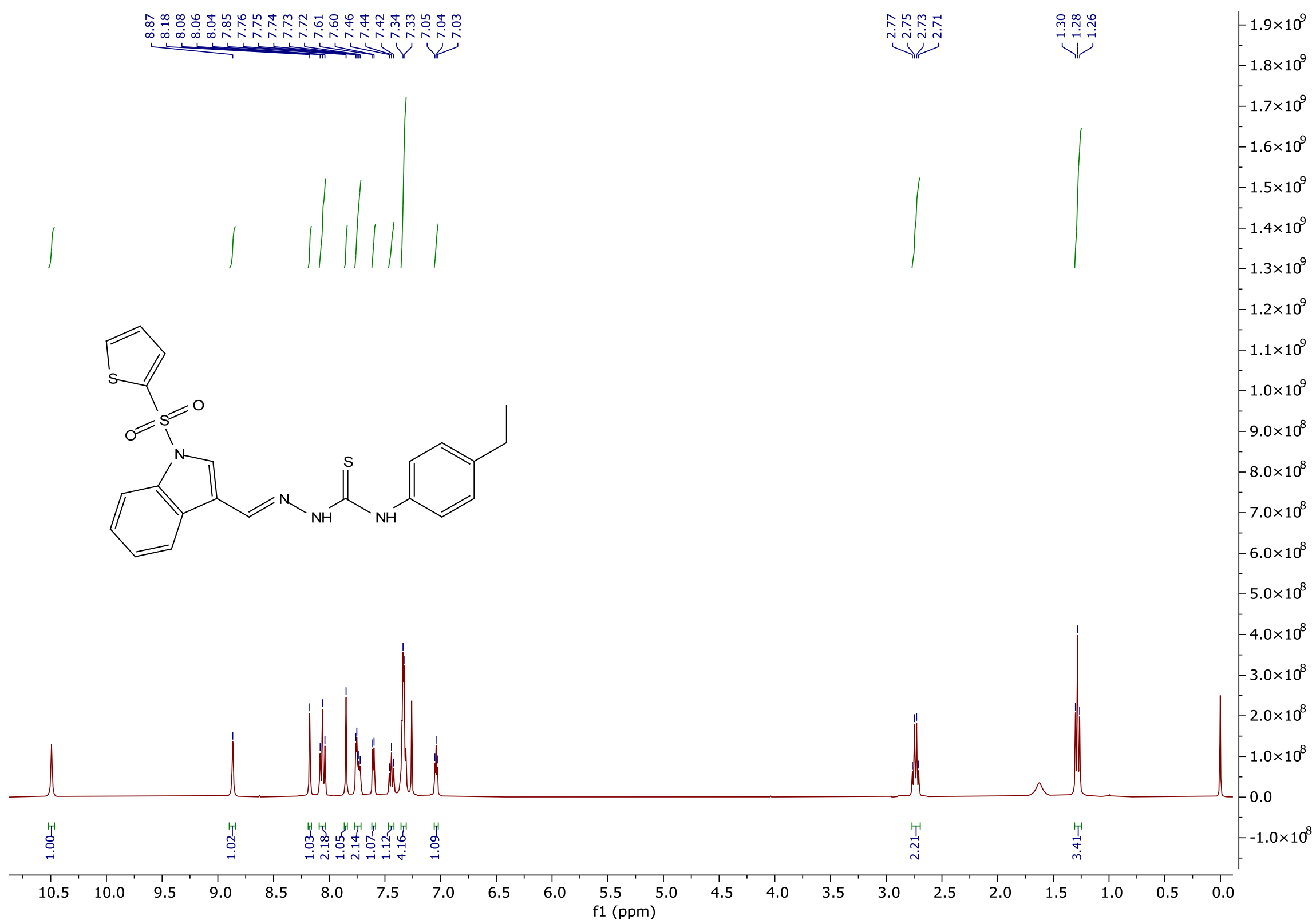

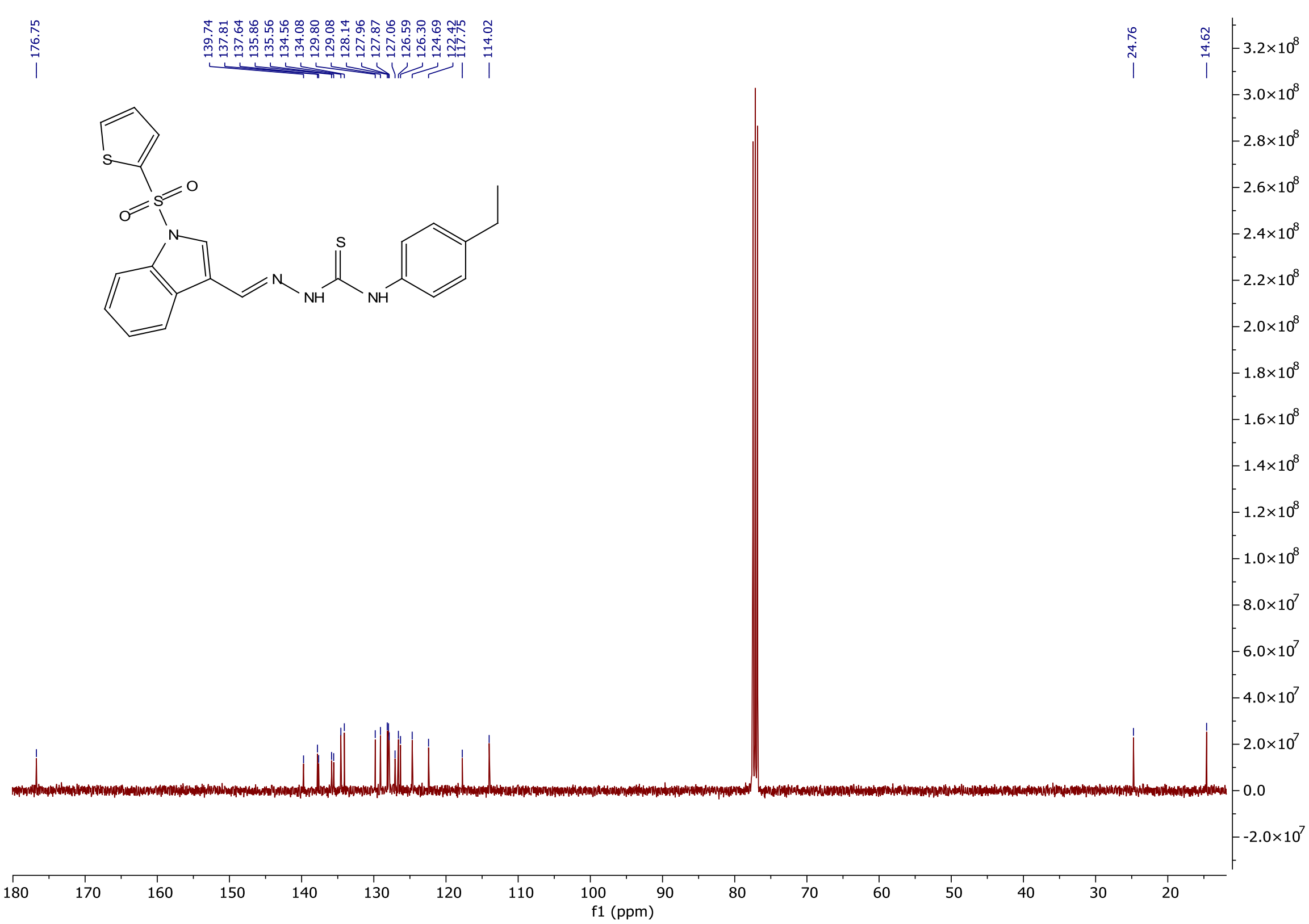

HRMS of 5a

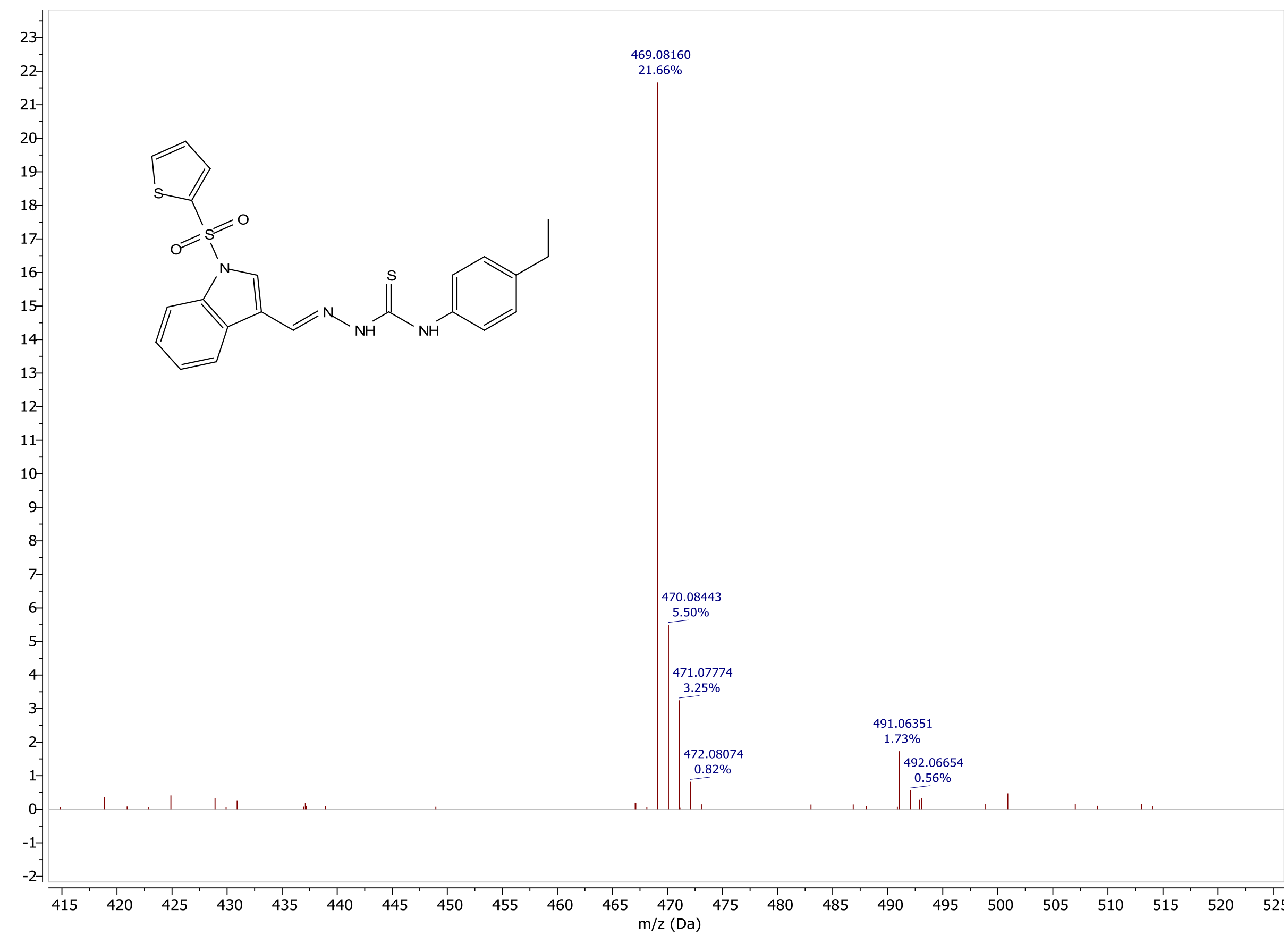
469.08160
21.66%
470.08443
5.50%
471.07774
3.25%
472.08074
0.82%
491.06351
1.73%
492.06654
0.56%
m/z (Da)

^{1}H & ^{13}C NMR Spectra of 5b

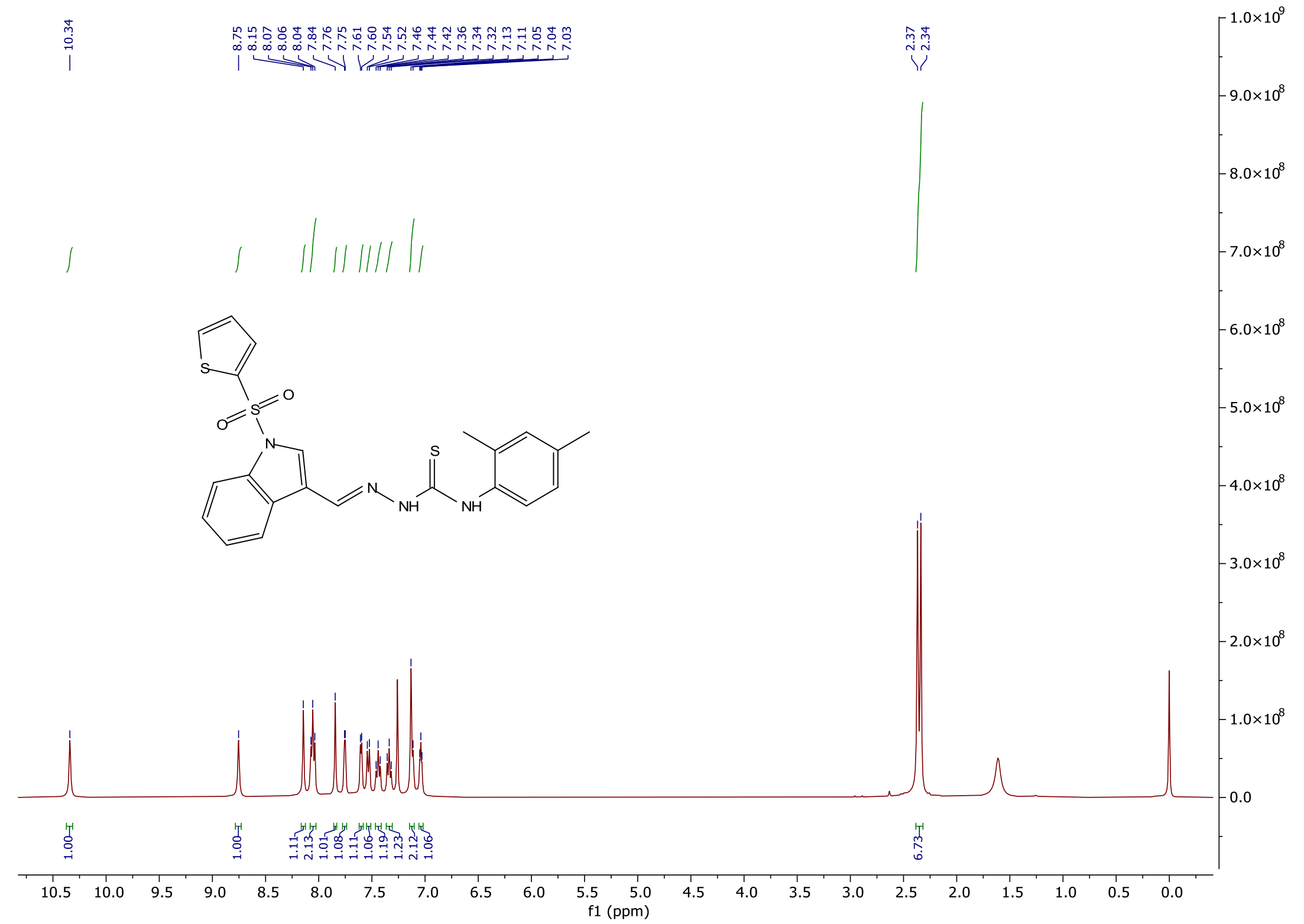

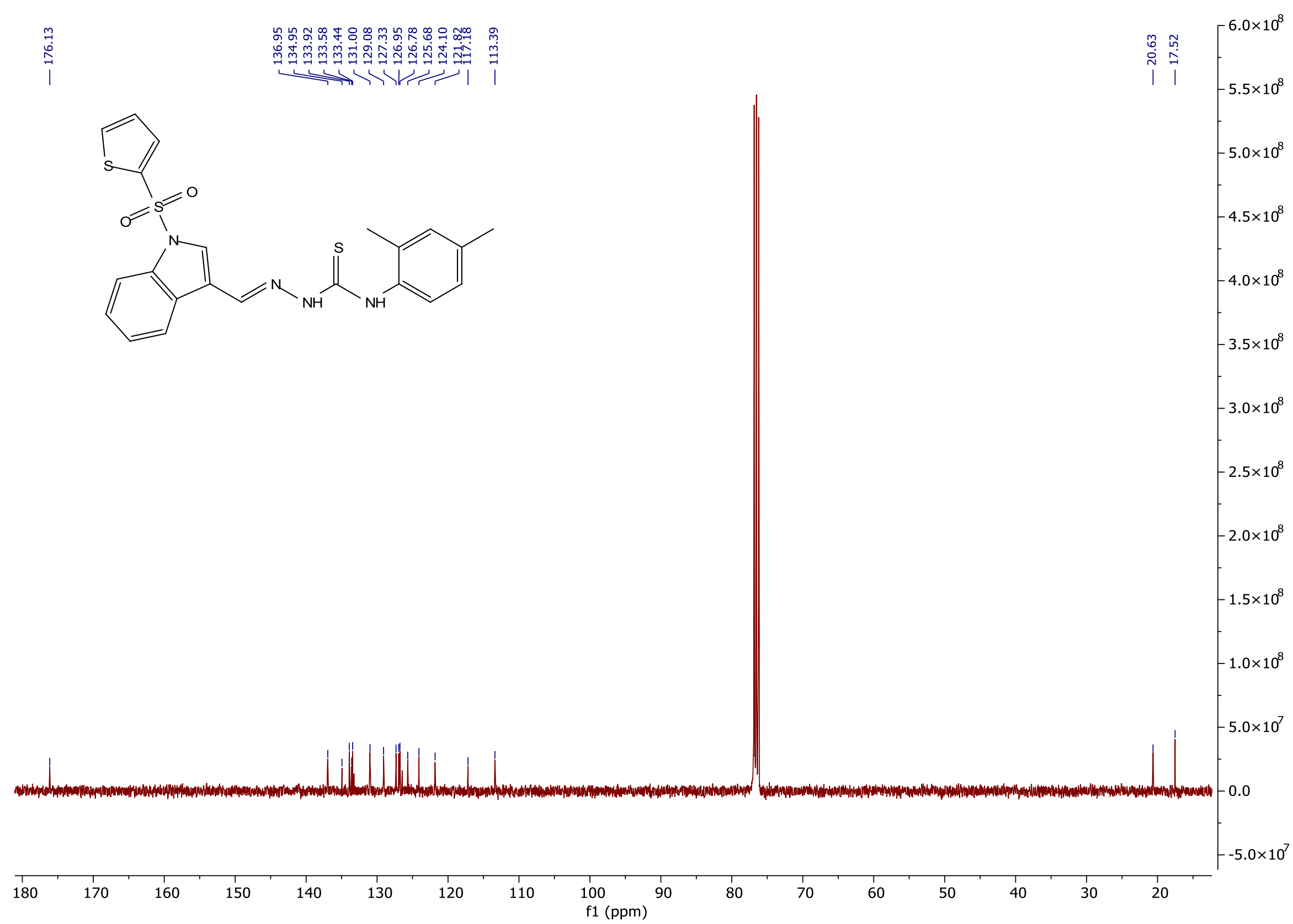

HRMS of 5b

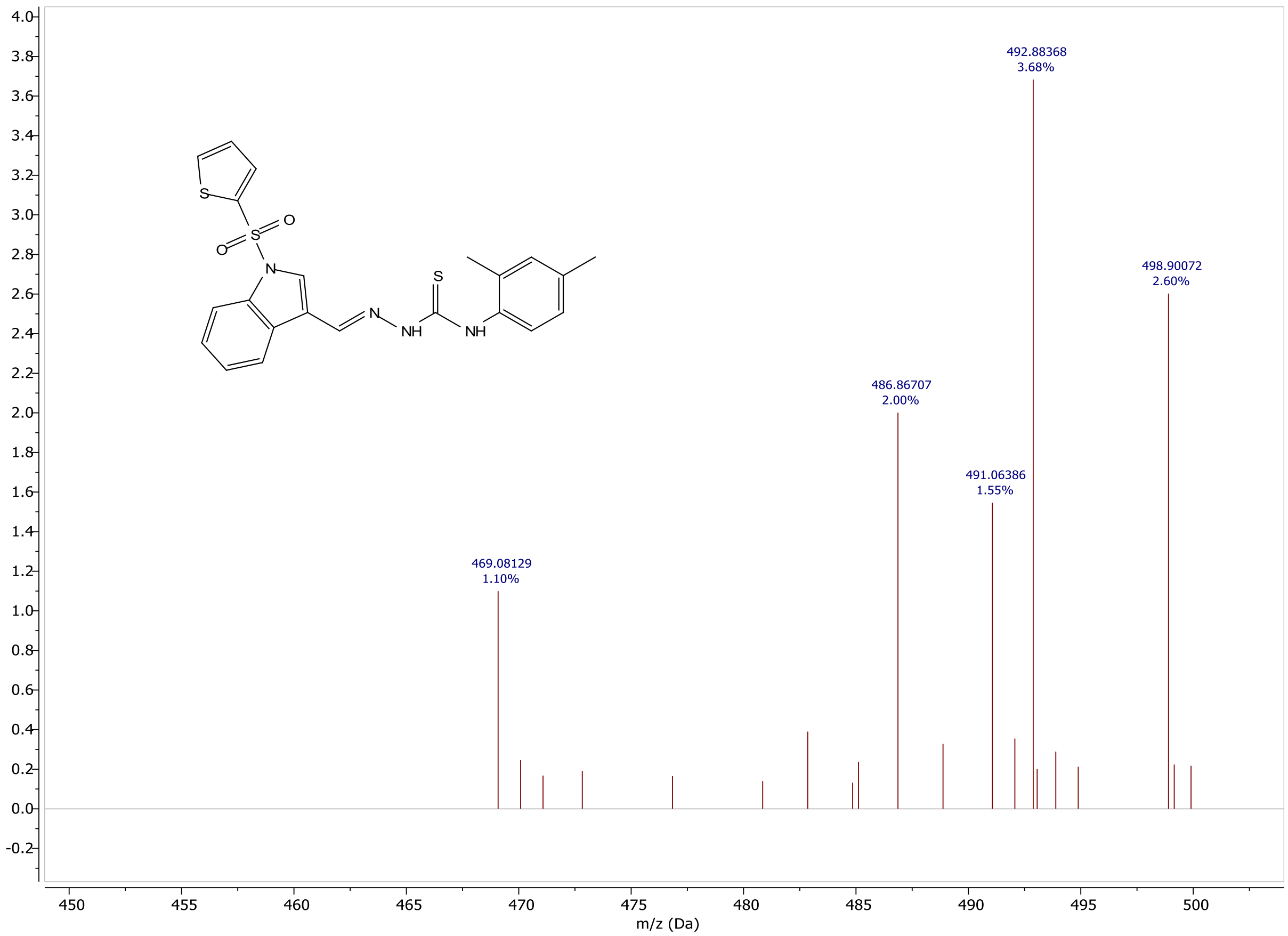
492.88368
3.68%
498.90072
2.60%
486.86707
2.00%
491.06386
1.55%
469.08129
1.10%
m/z (Da)
S
O
O
N
N
NH
NH
S

^{1}H & ^{13}C NMR Spectra of 5c

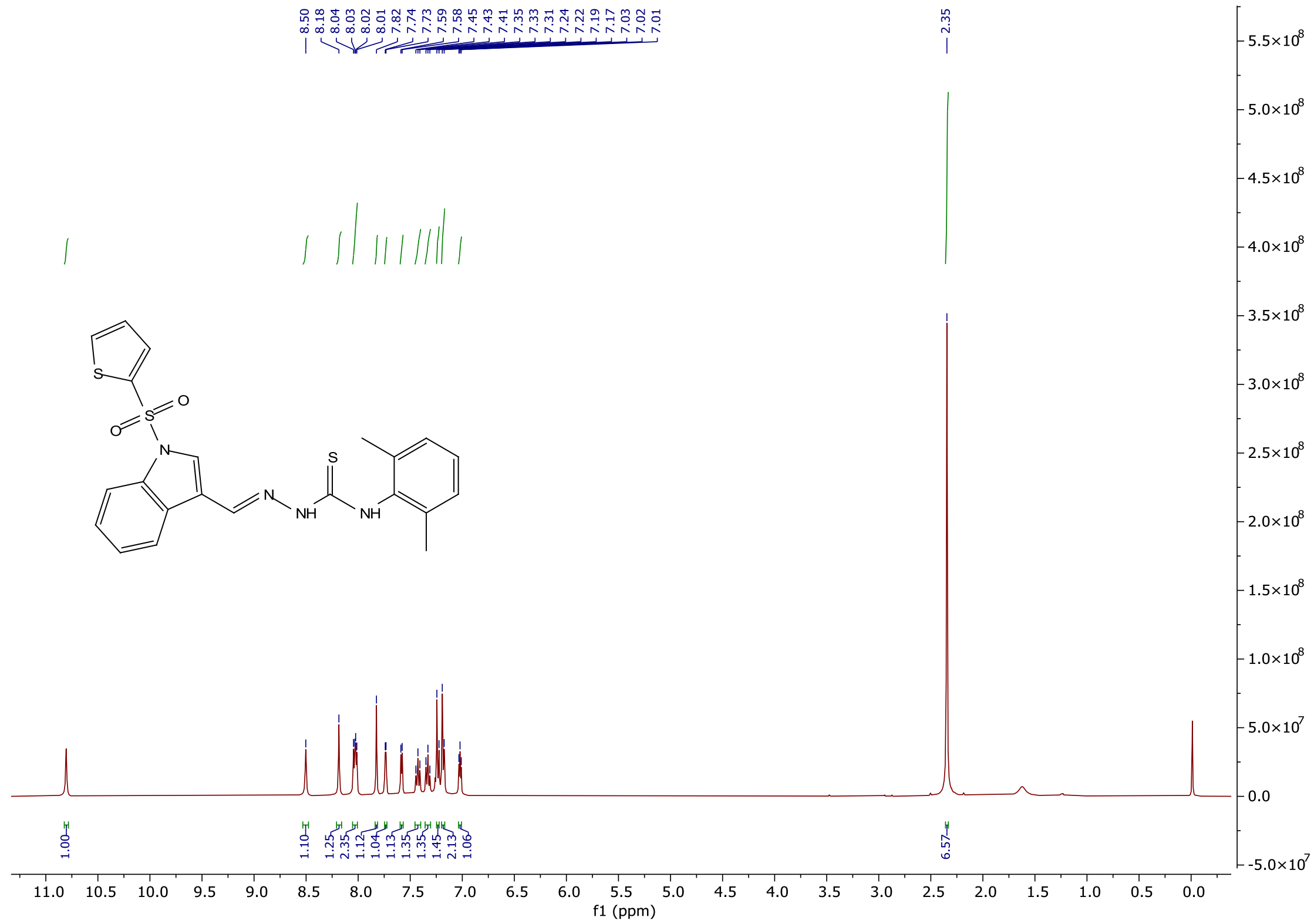

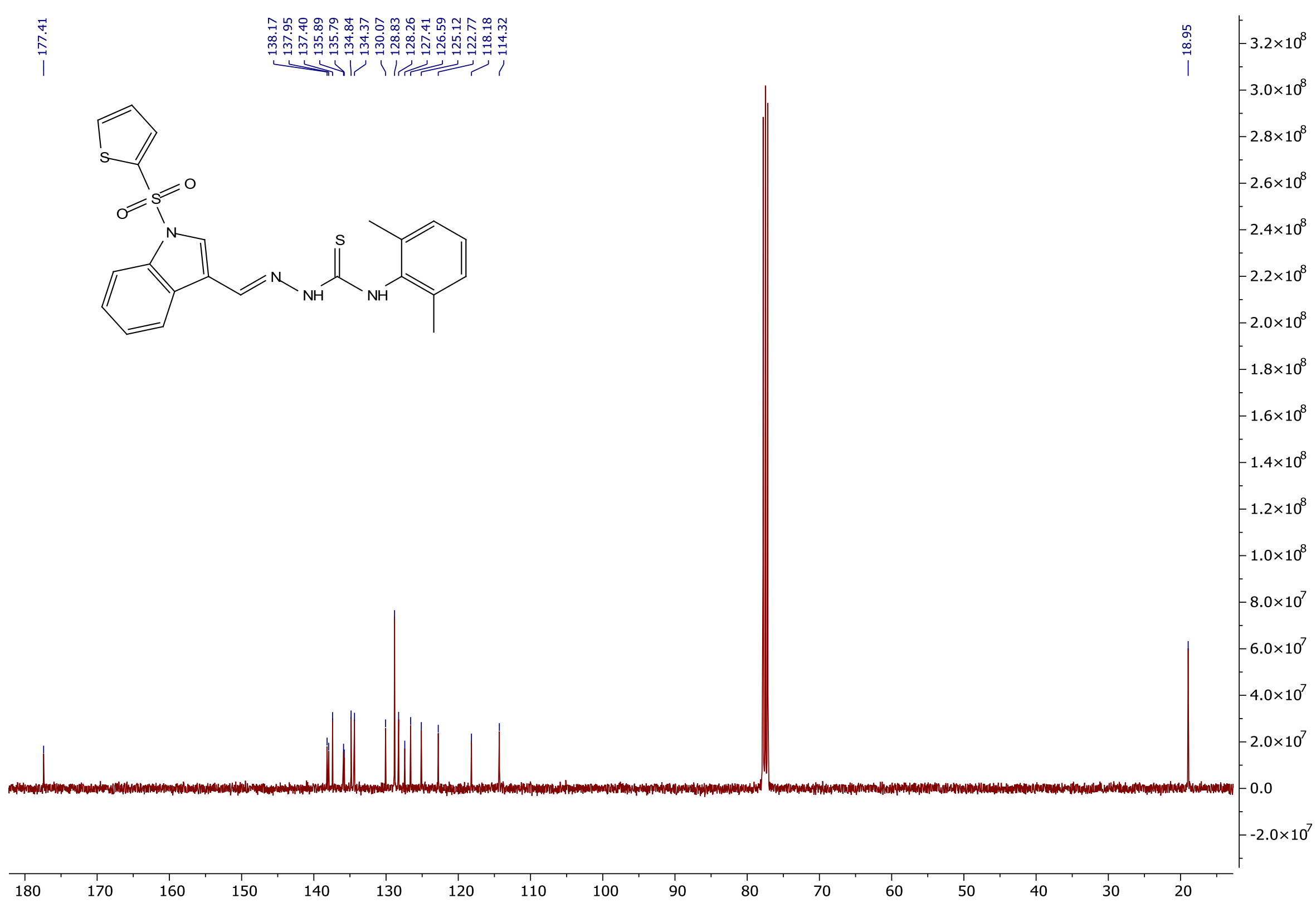

HRMS of 5c

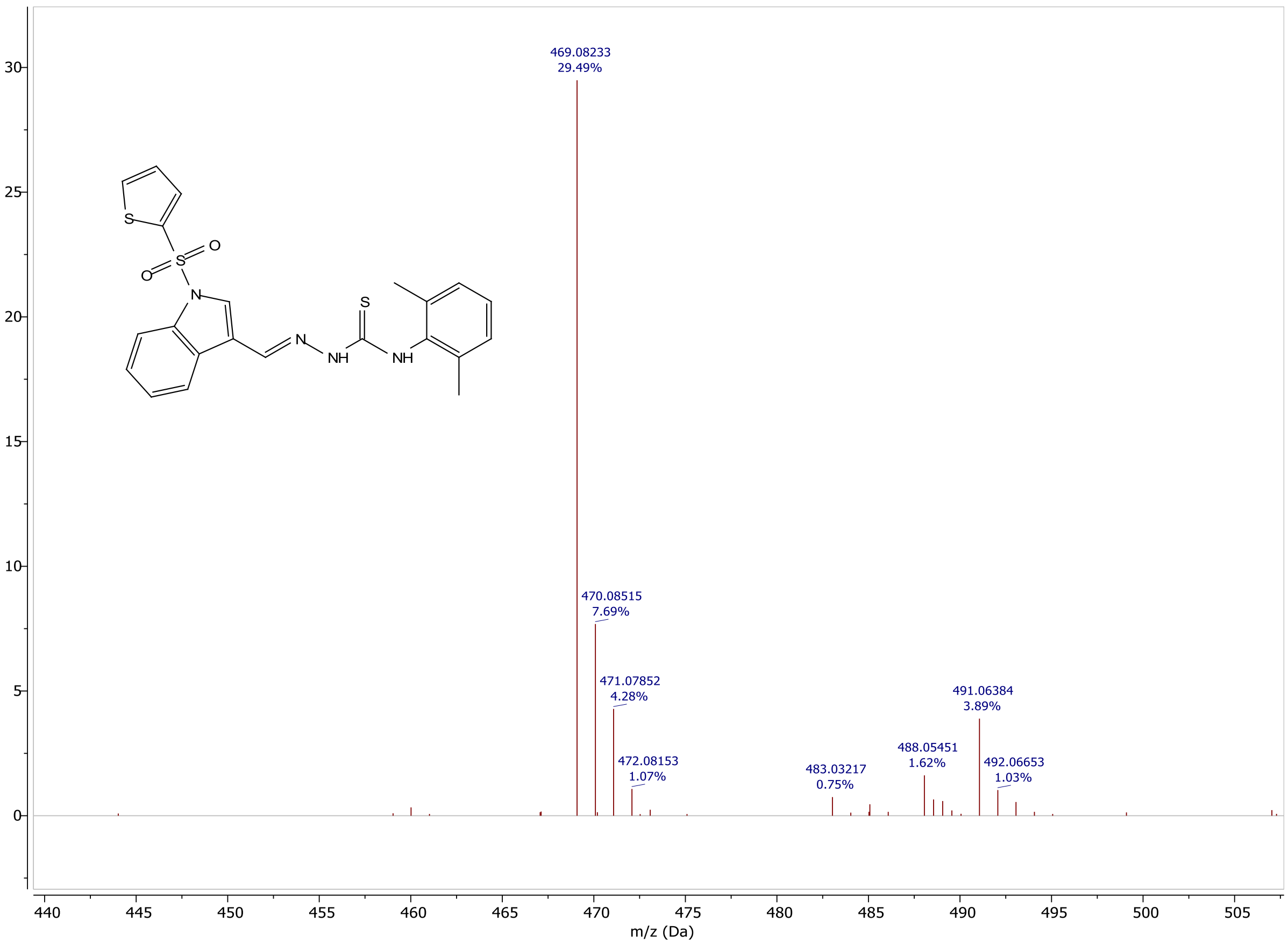
469.08233
29.49%
470.08515
7.69%
471.07852
4.28%
472.08153
1.07%
483.03217
0.75%
488.05451
1.62%
491.06384
3.89%
492.06653
1.03%
m/z (Da)

^{1}H & ^{13}C NMR Spectra of 5d

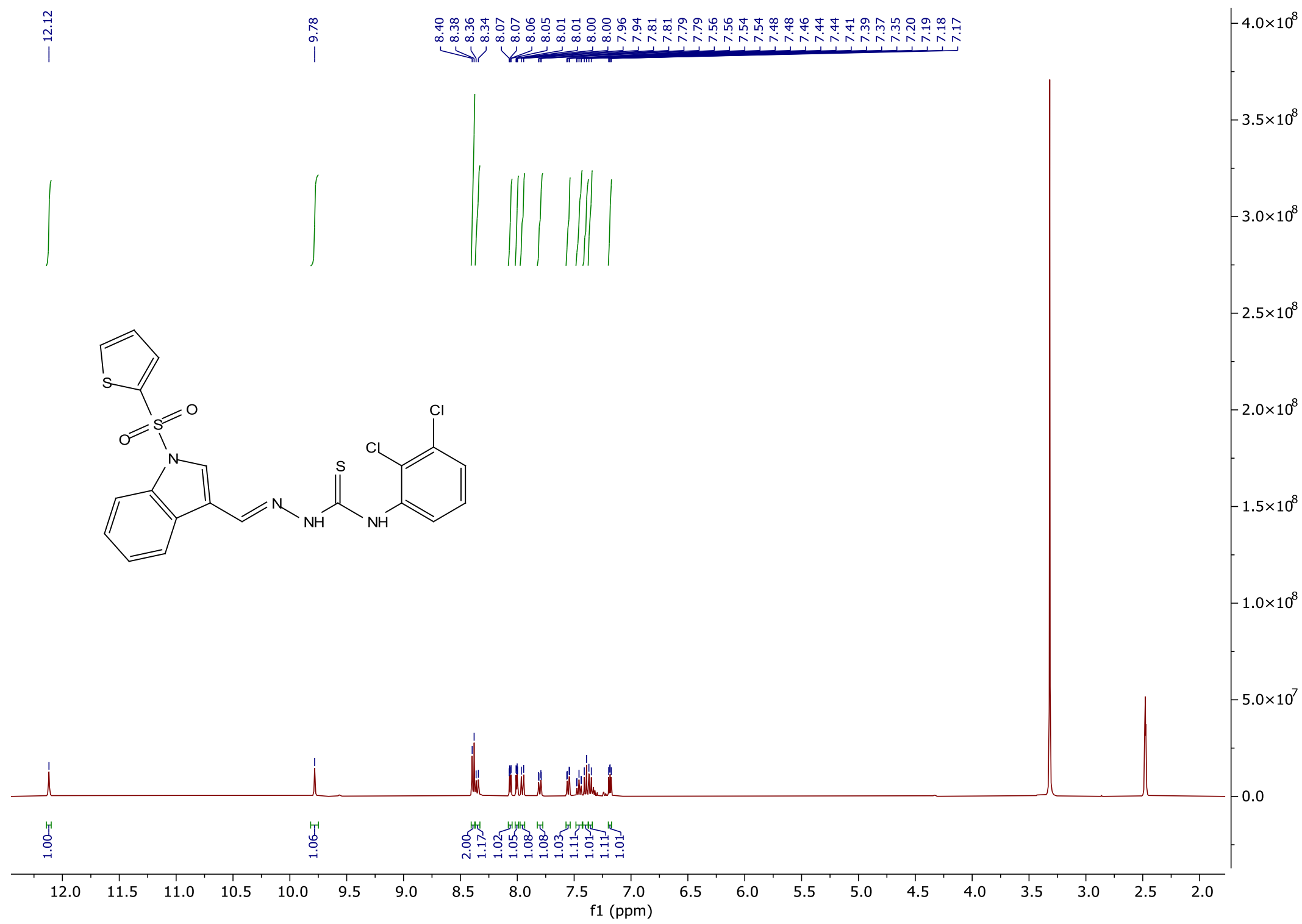

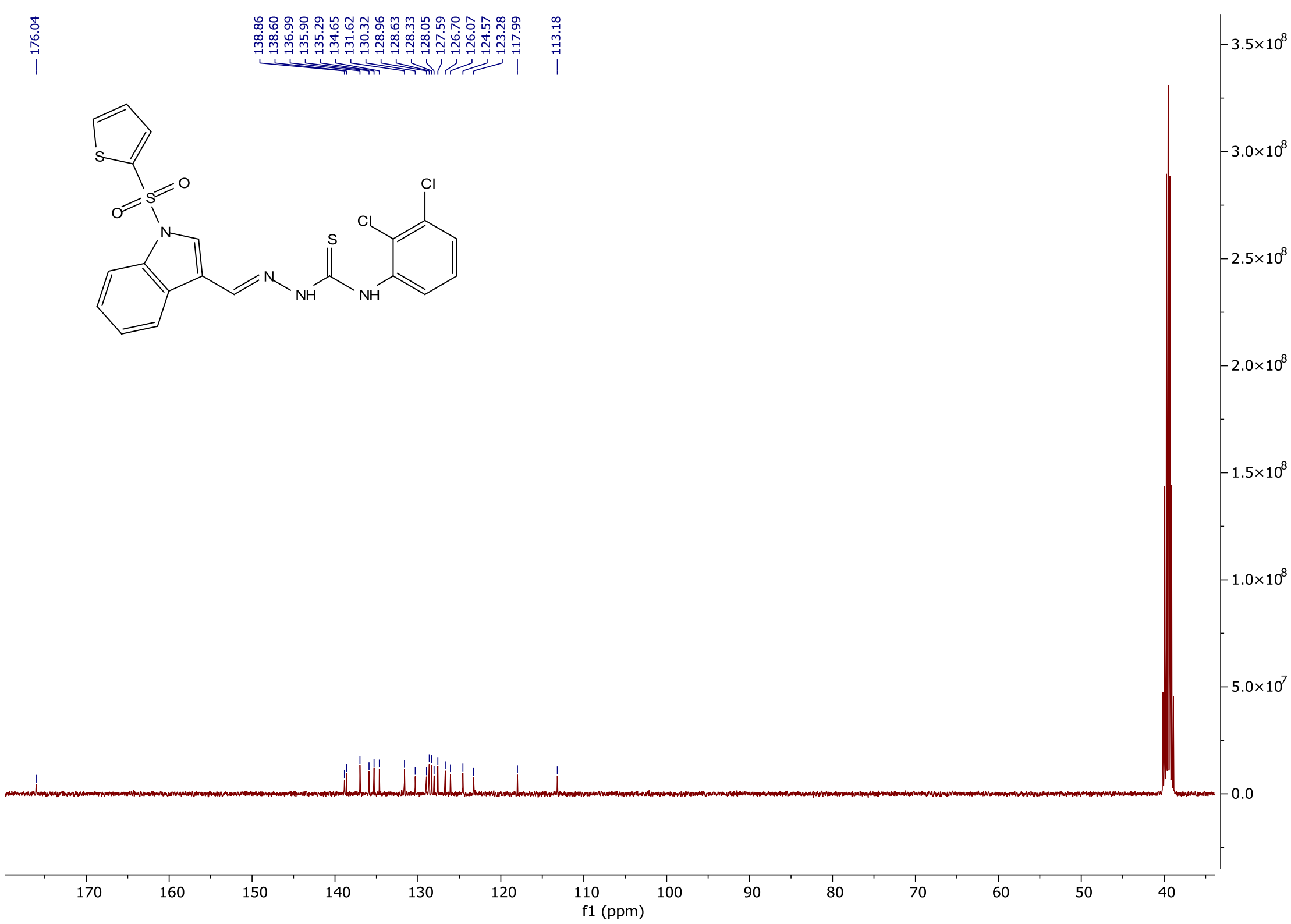

HRMS of 5d

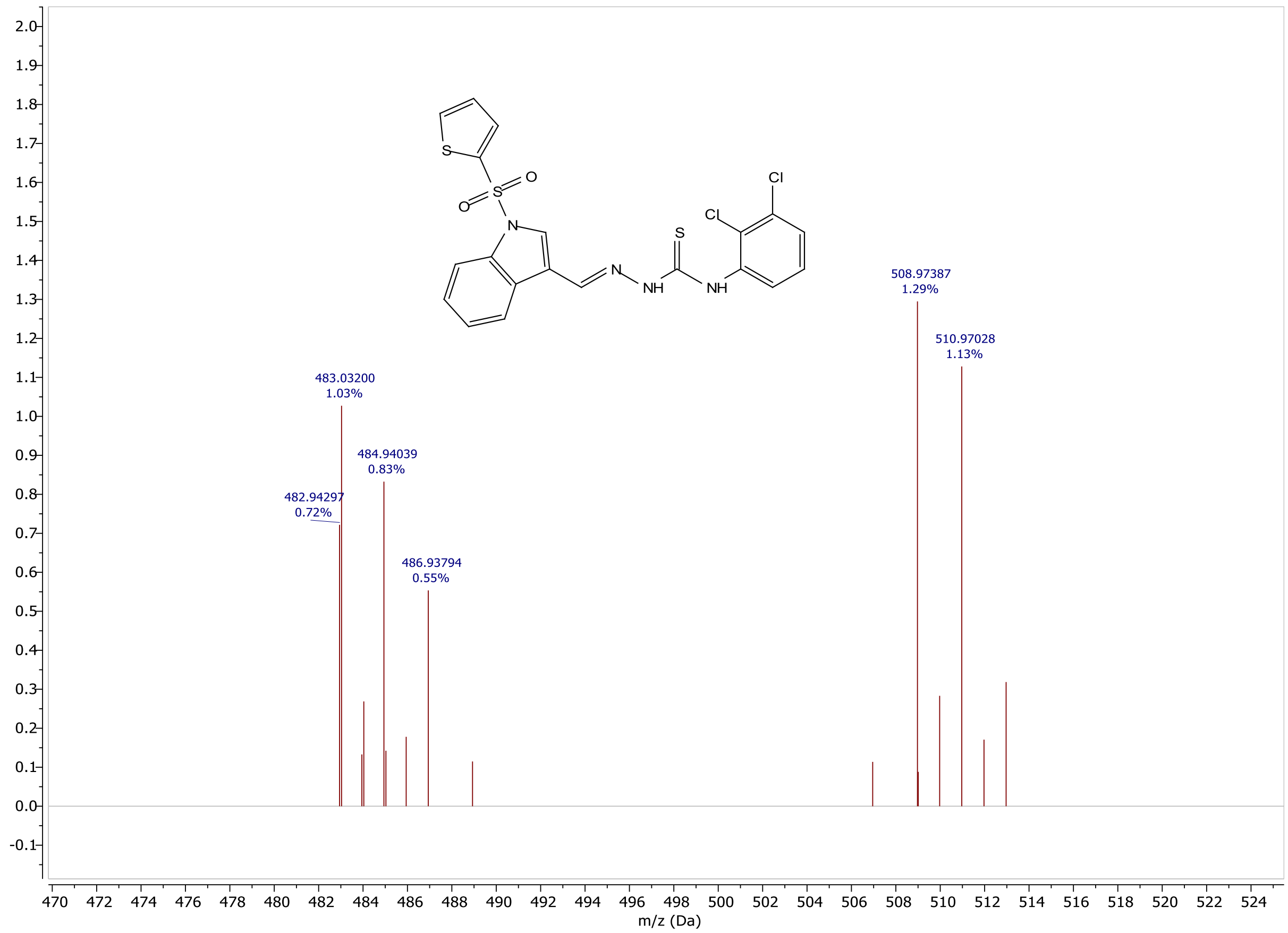

HPLC of 5d

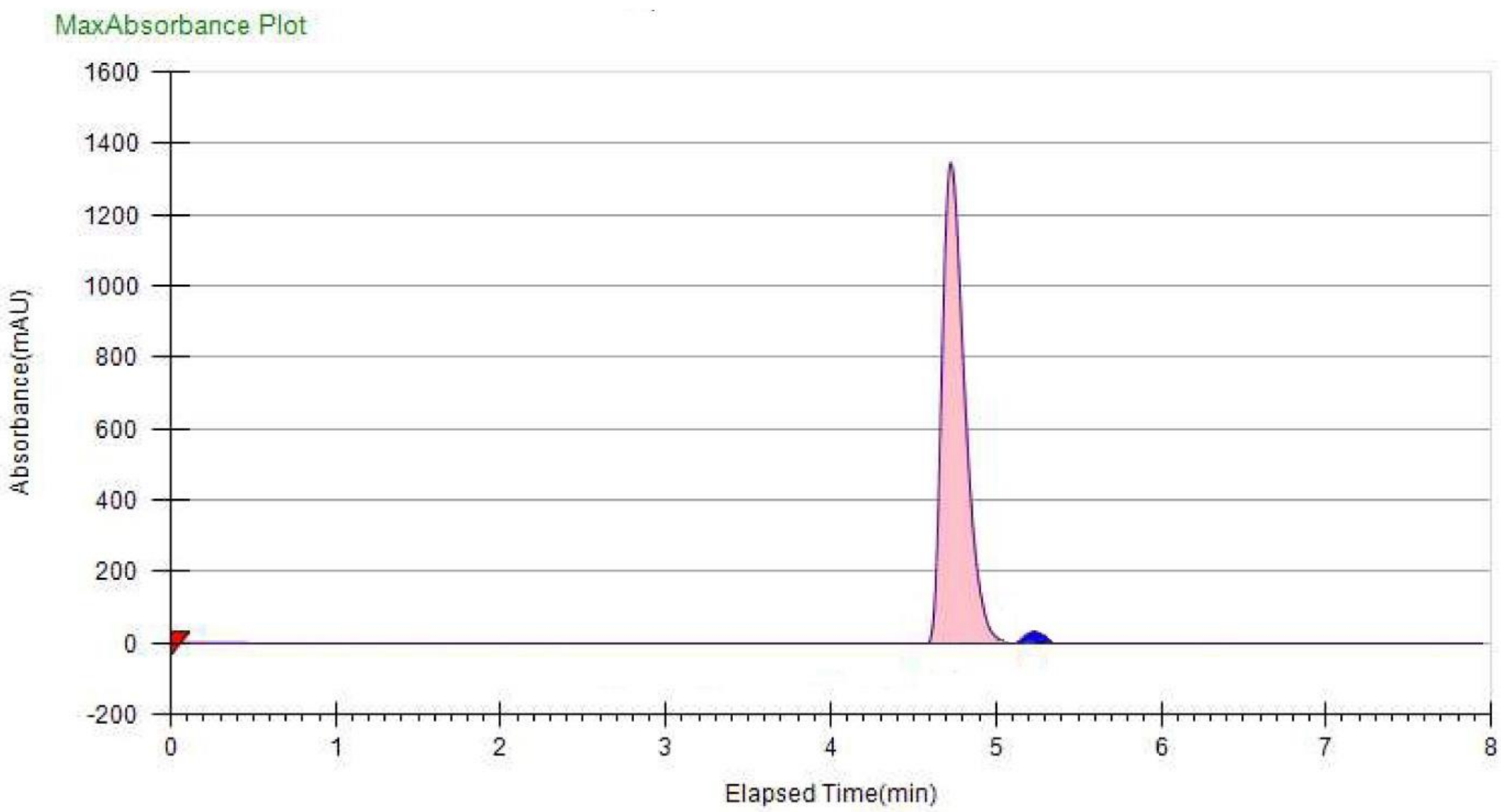
MaxAbsorbance Plot
1600
1400
1200
1000
800
600
400
200
0
-200
Absorbance(mAU)
0
1
2
3
4
5
6
7
8
Elapsed Time(min)

^{1}H & ^{13}C NMR Spectra of 5e

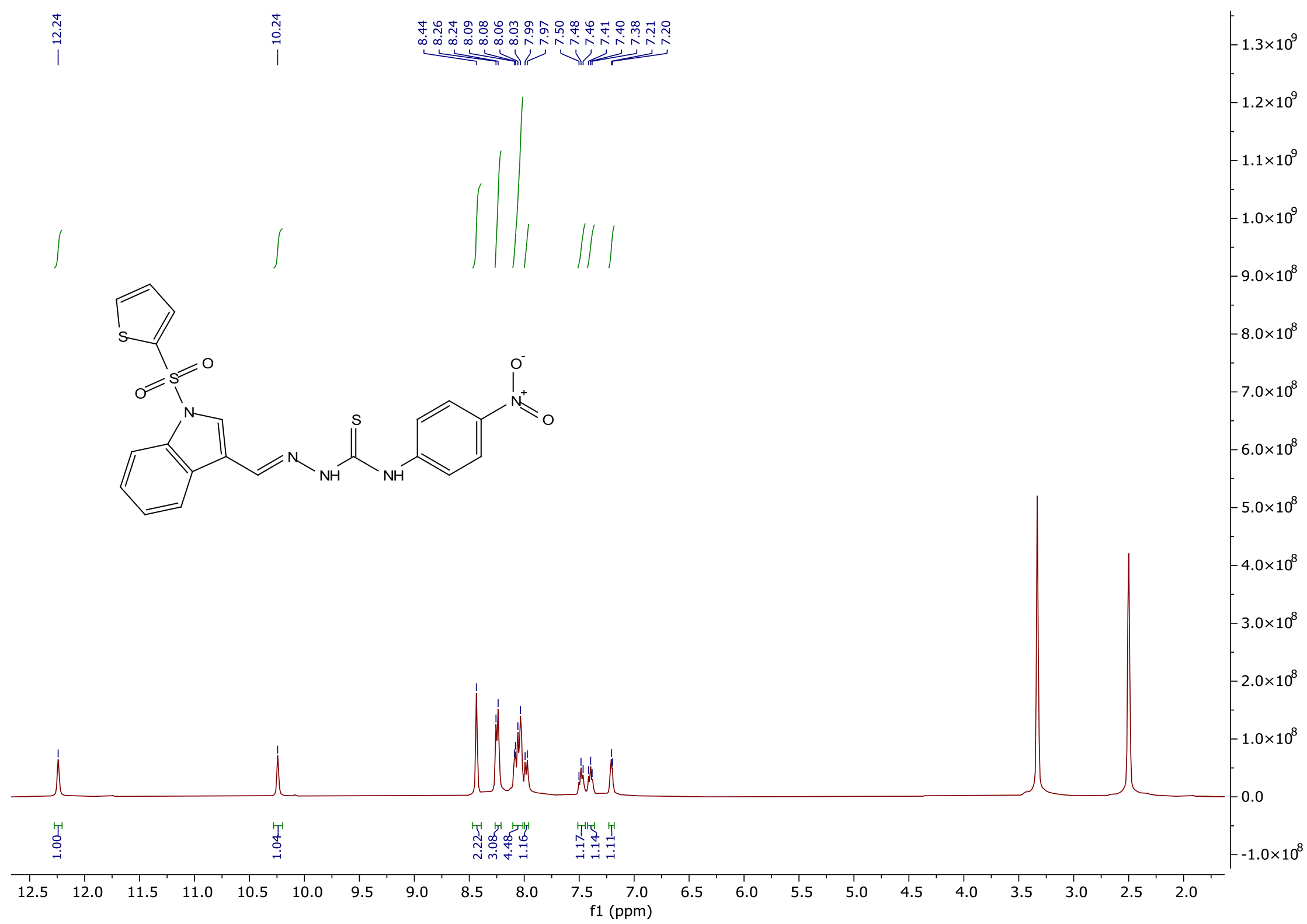

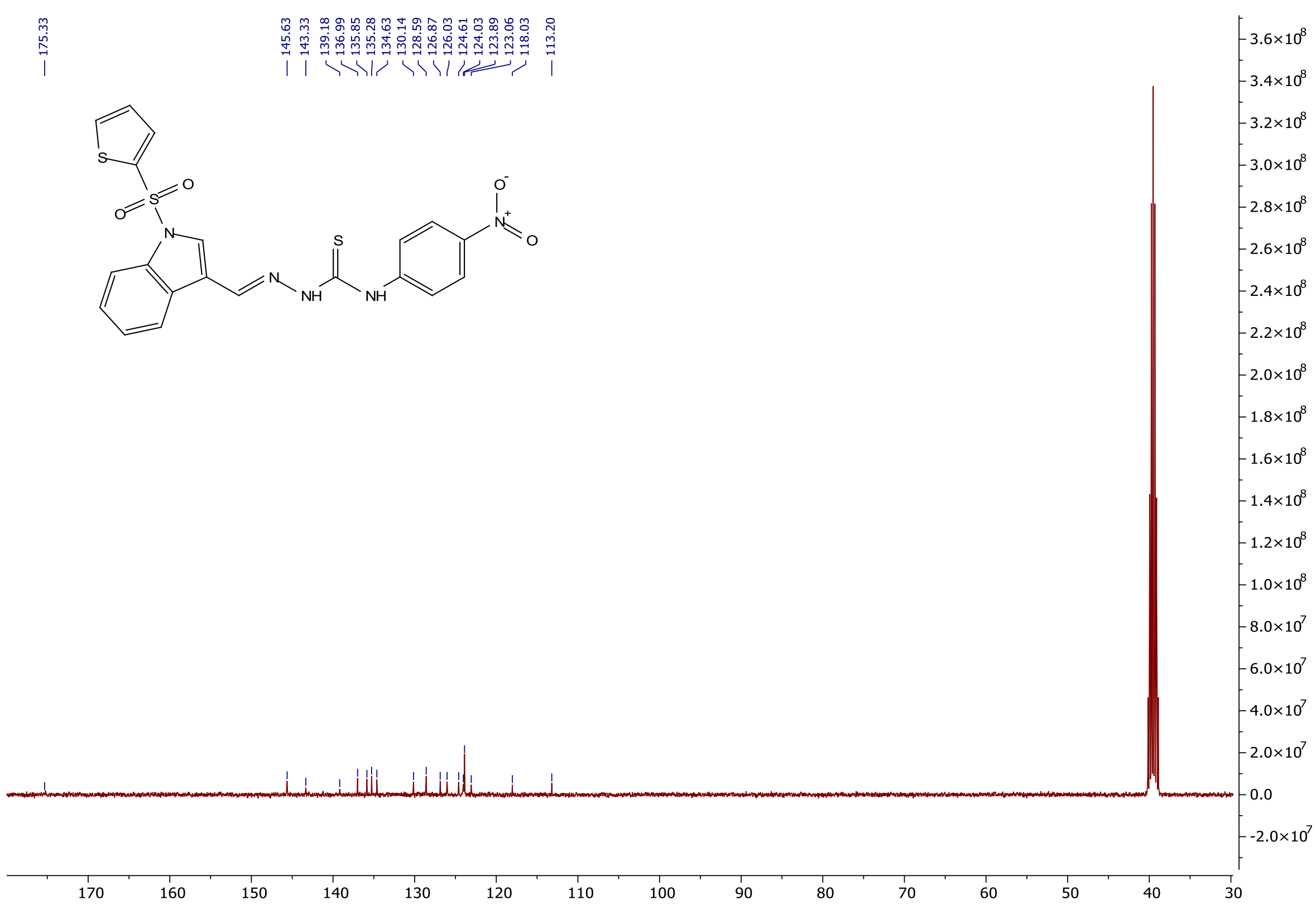

HRMS of 5e

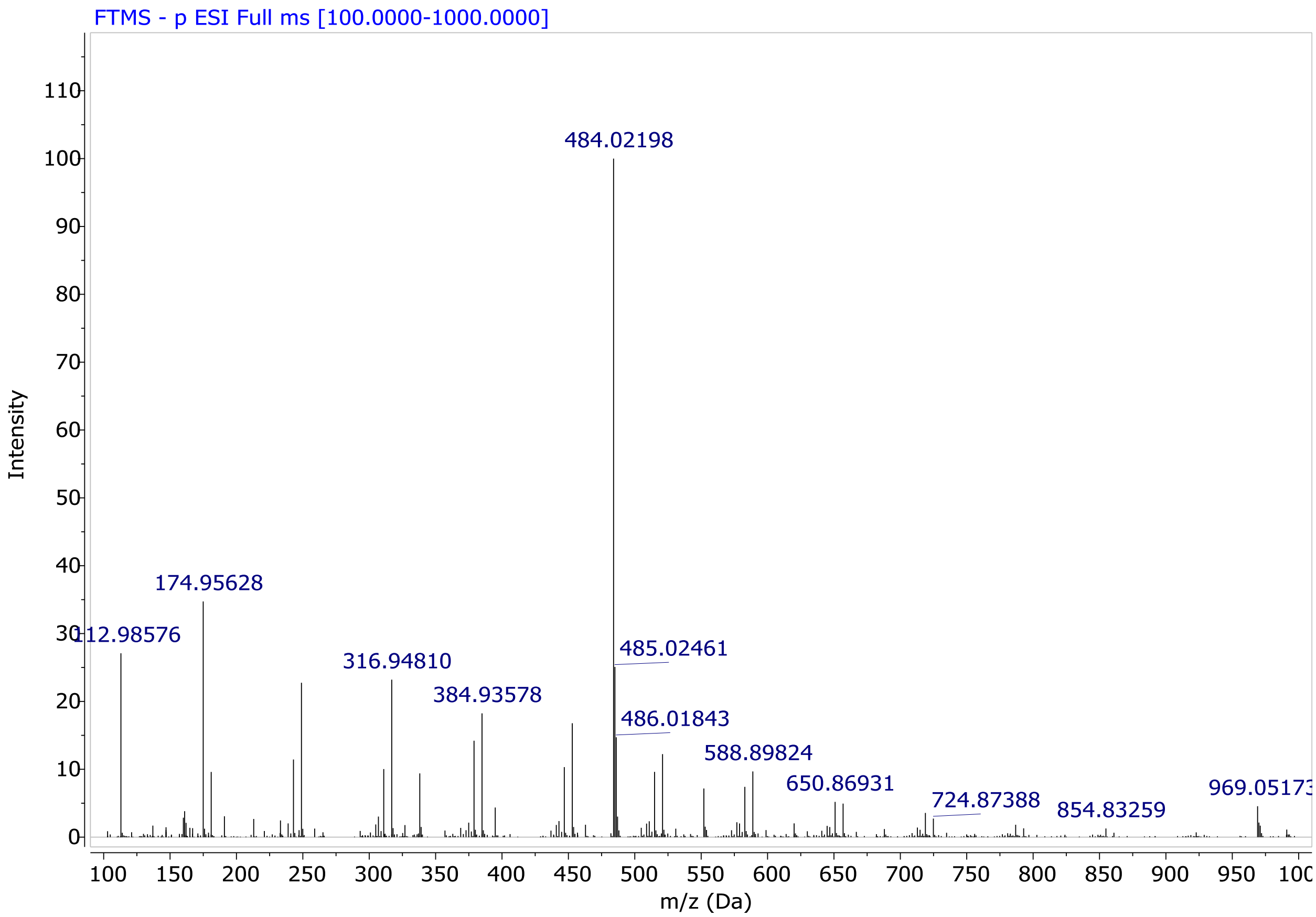

FTMS - p ESI Full ms [100.0000-1000.0000]
484.02198
174.95628
112.98576
316.94810
384.93578
485.02461
486.01843
588.89824
650.86931
724.87388
854.83259
969.05173
Intensity
m/z (Da)

^{1}H & ^{13}C NMR Spectra of 5f

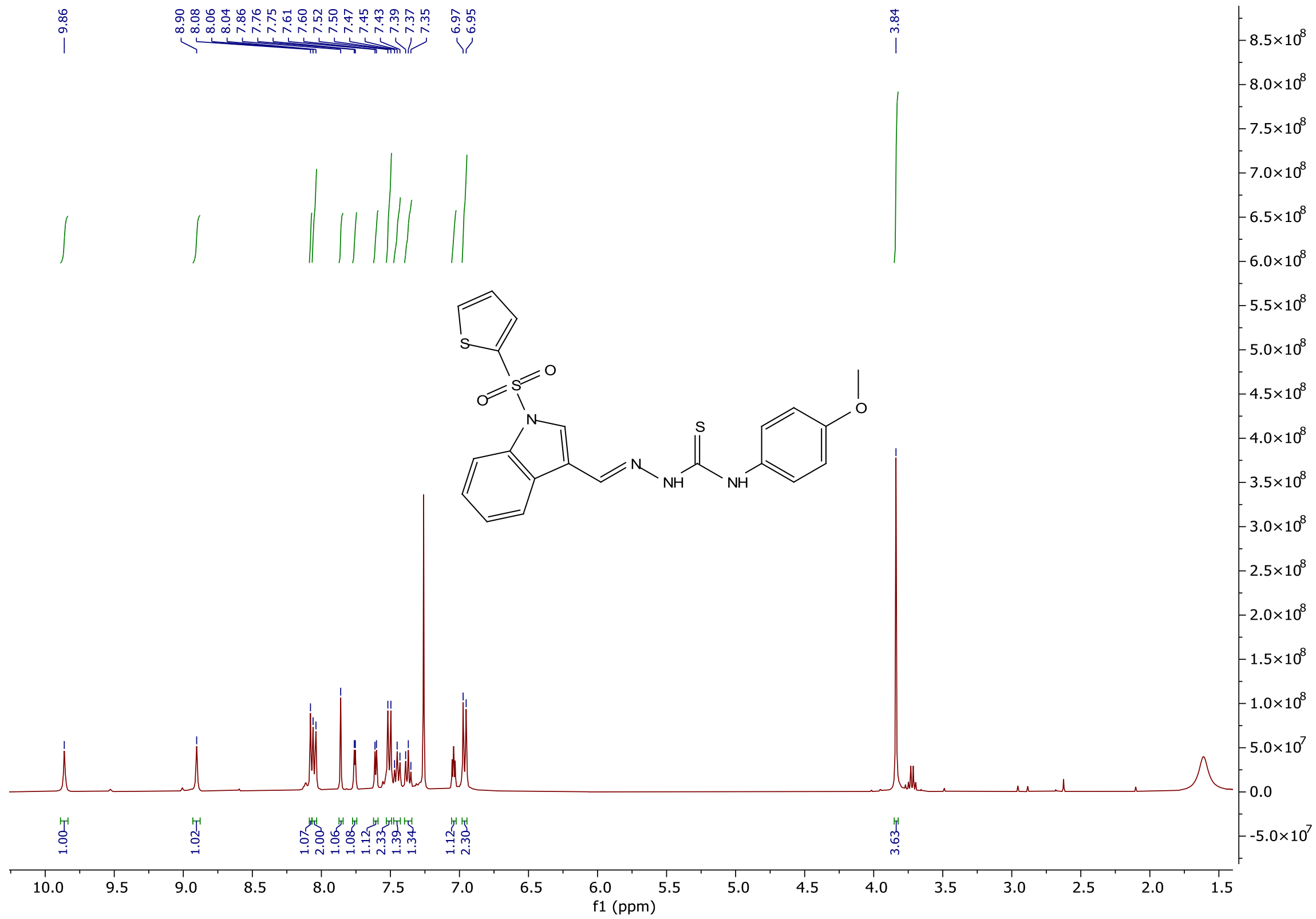

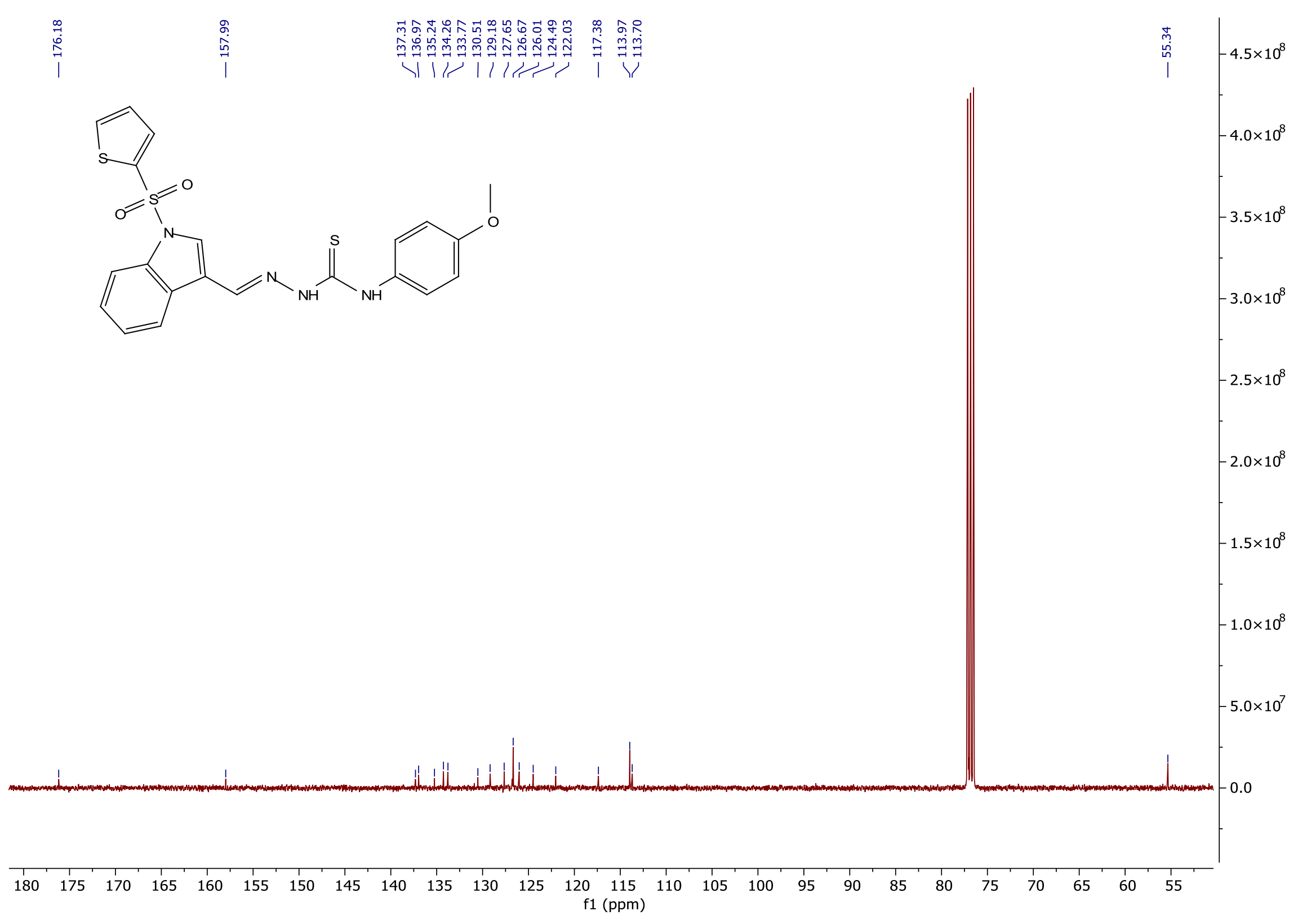

HRMS of 5f

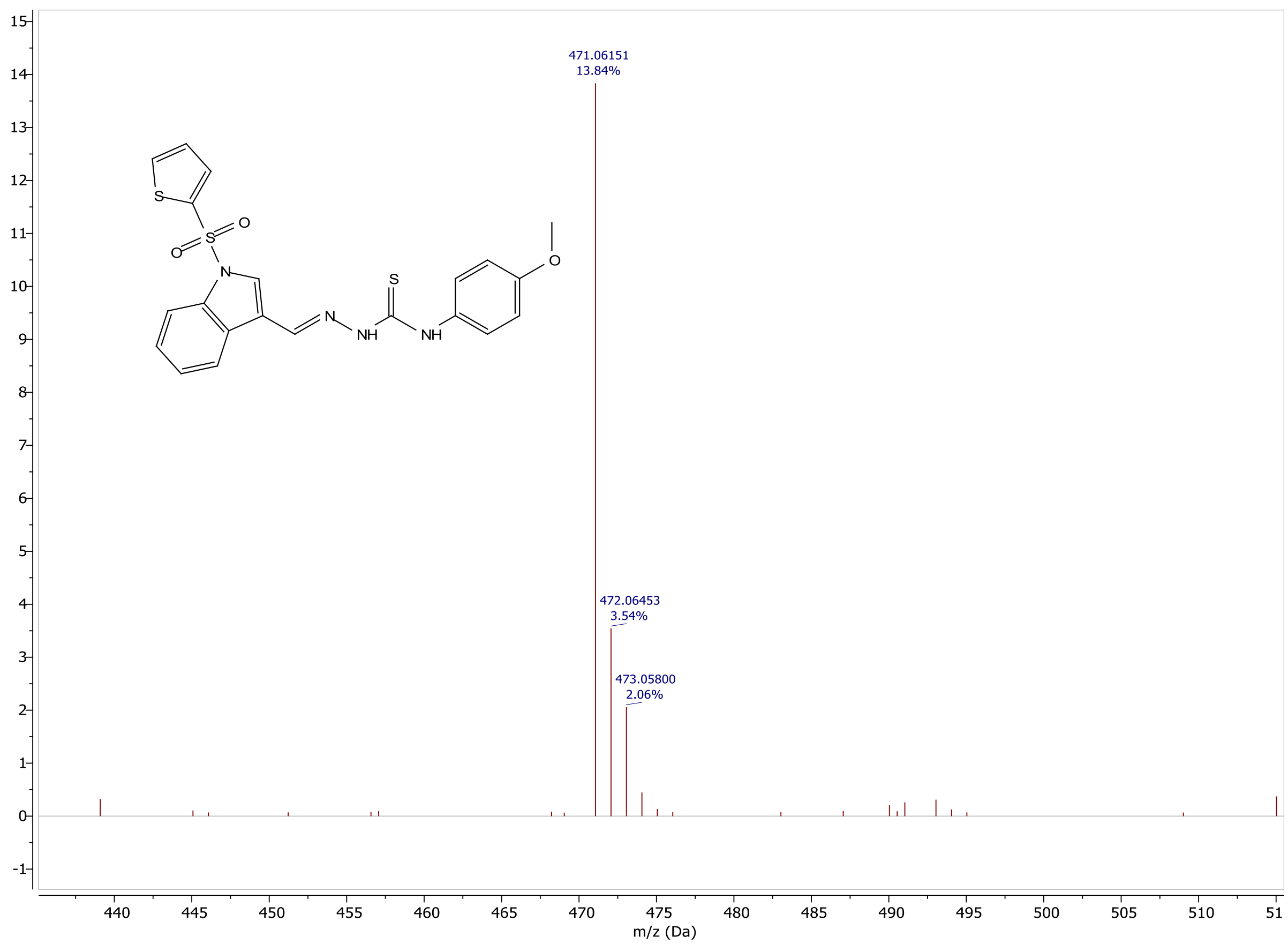

^{1}H & ^{13}C NMR Spectra of 5g

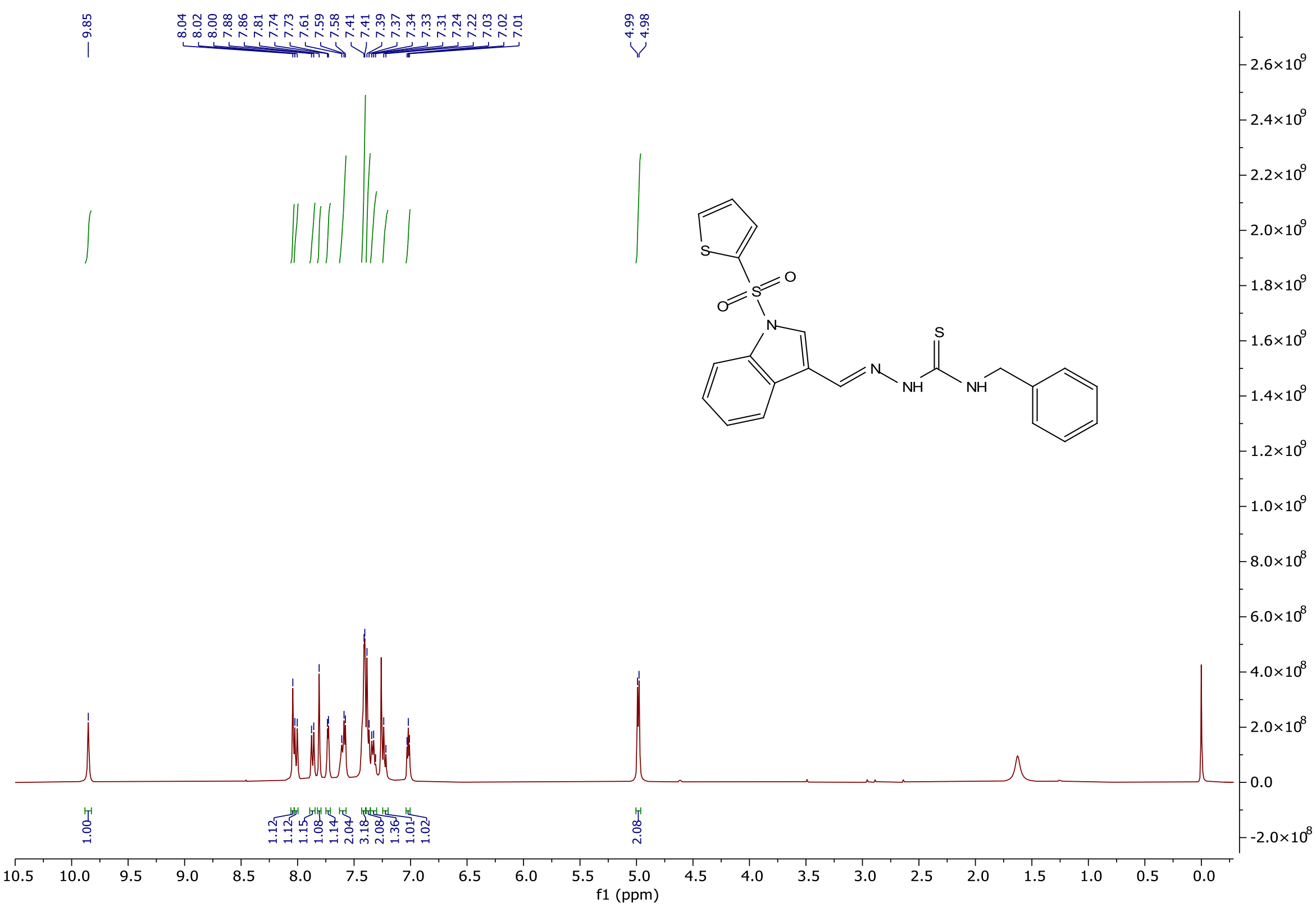

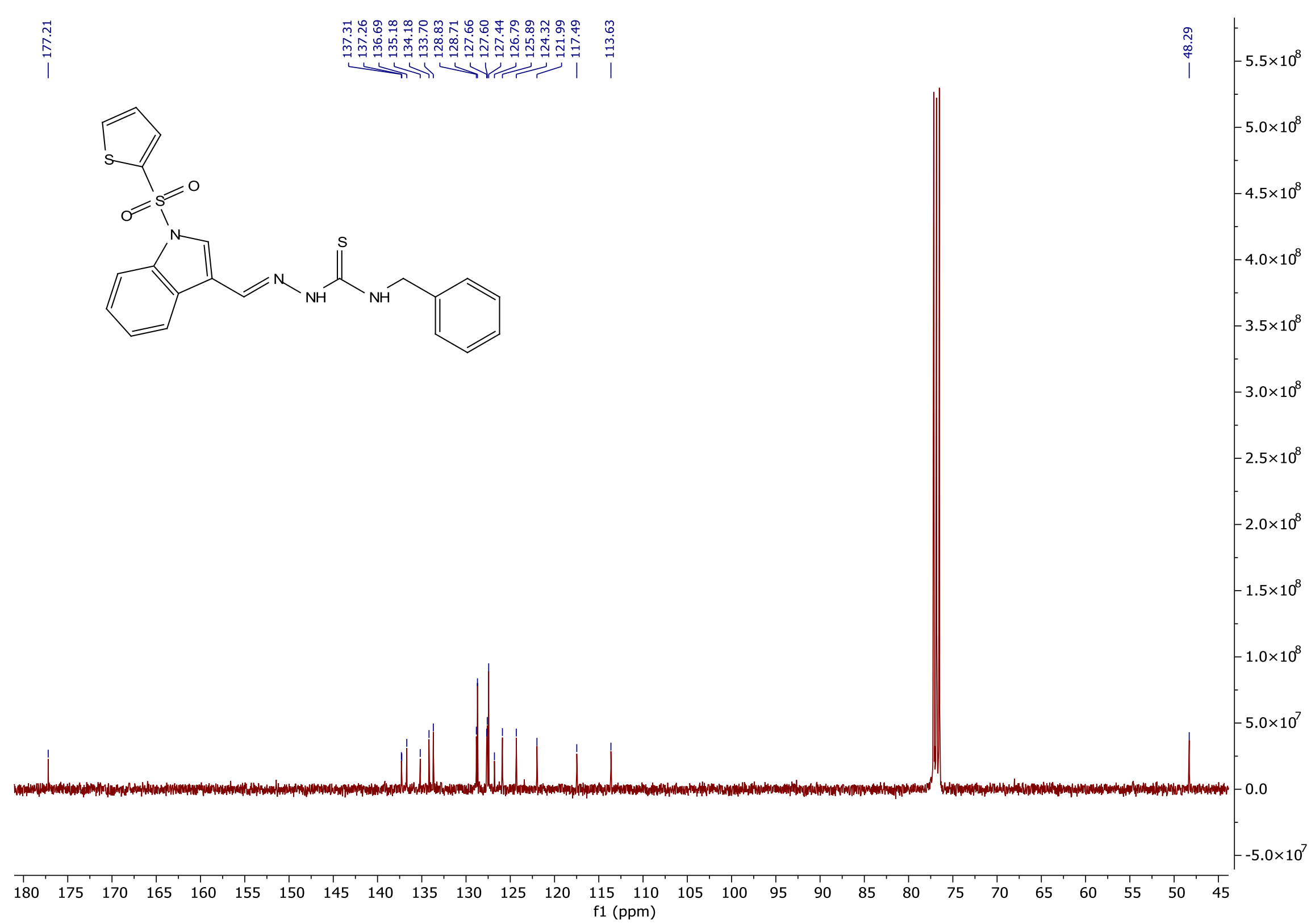

HRMS of 5g

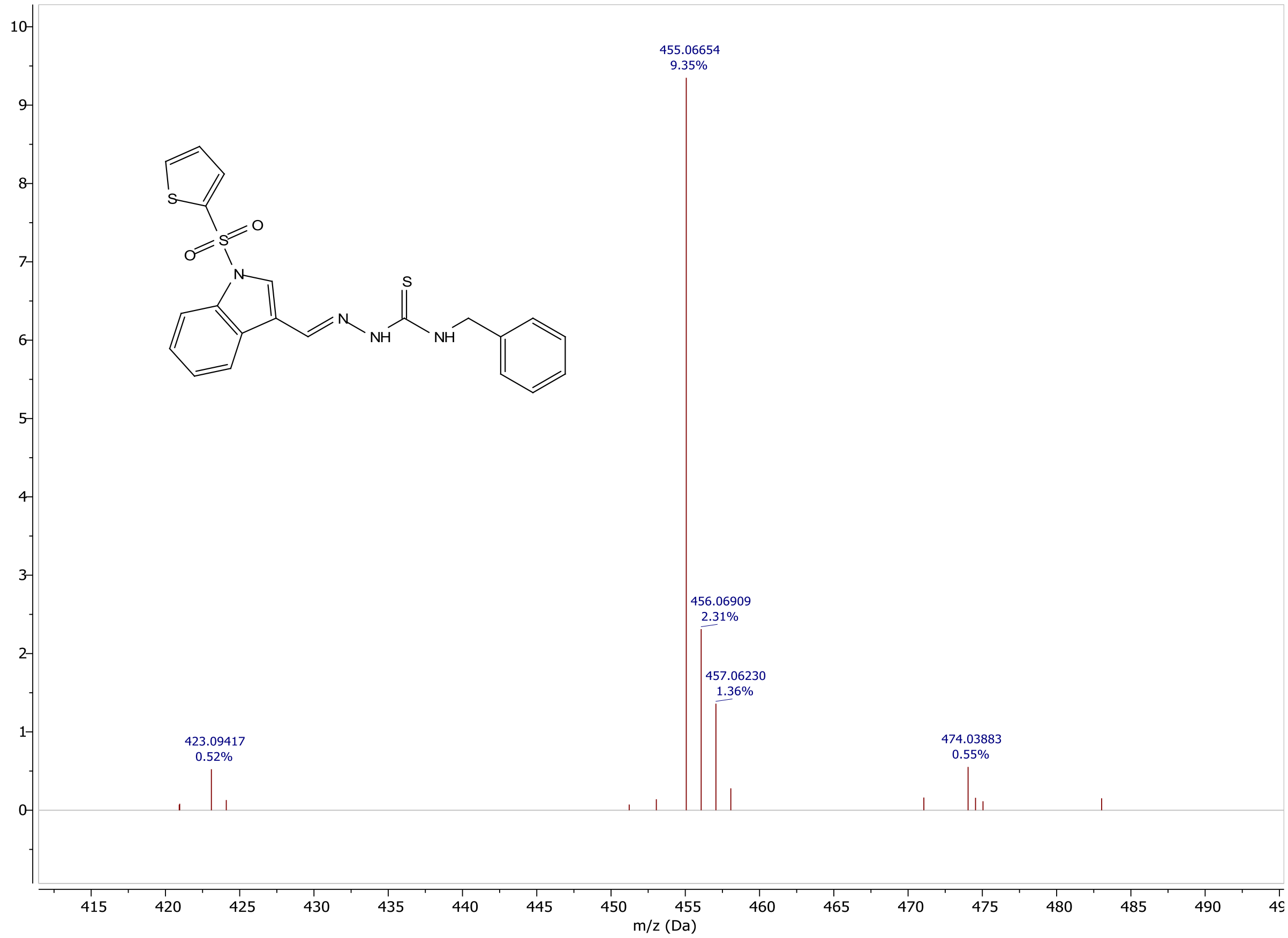

455.06654
9.35%
456.06909
2.31%
457.06230
1.36%
423.09417
0.52%
474.03883
0.55%
m/z (Da)

^1H & ^{13}C NMR Spectra of 5h

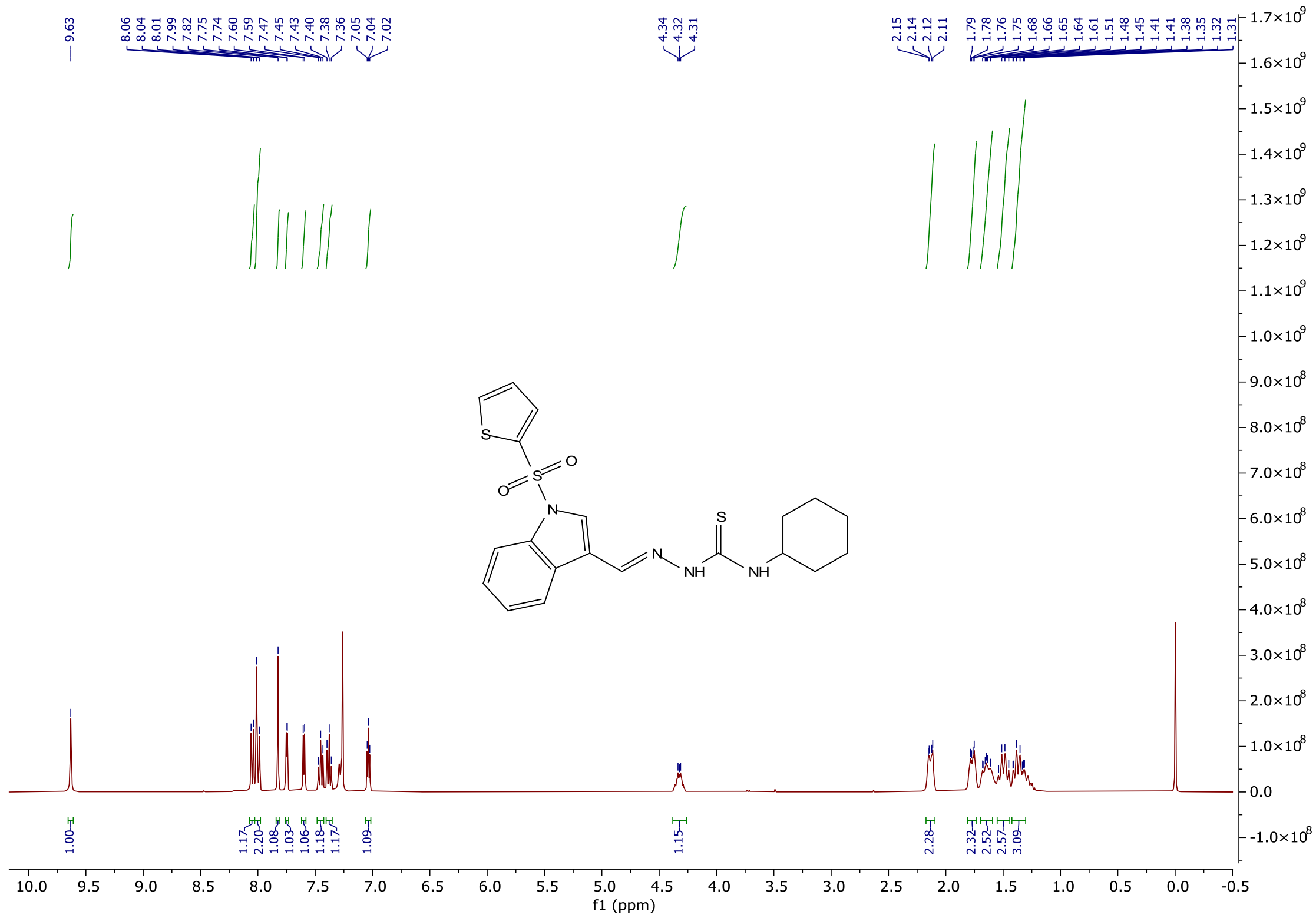

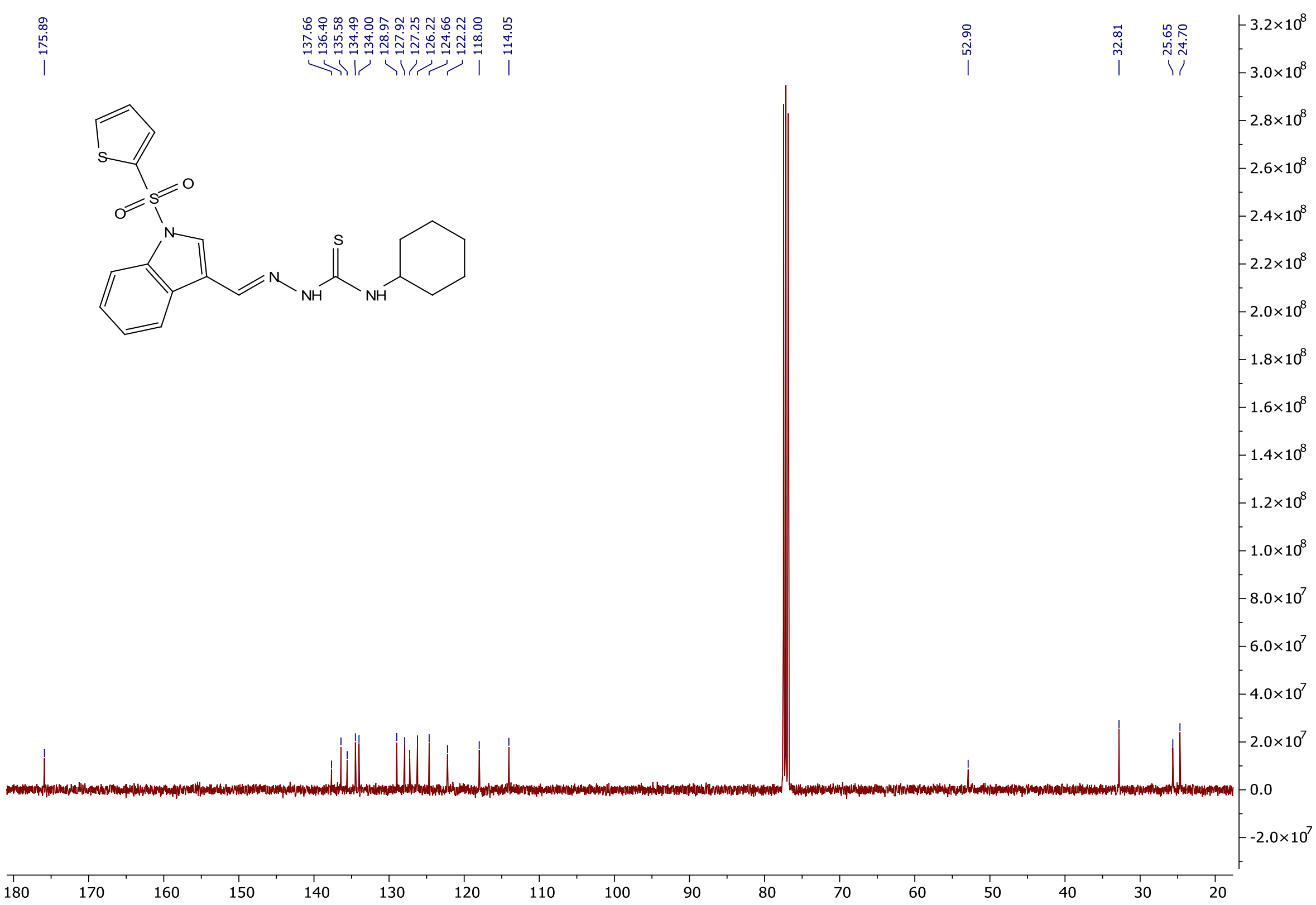

HRMS of 5h

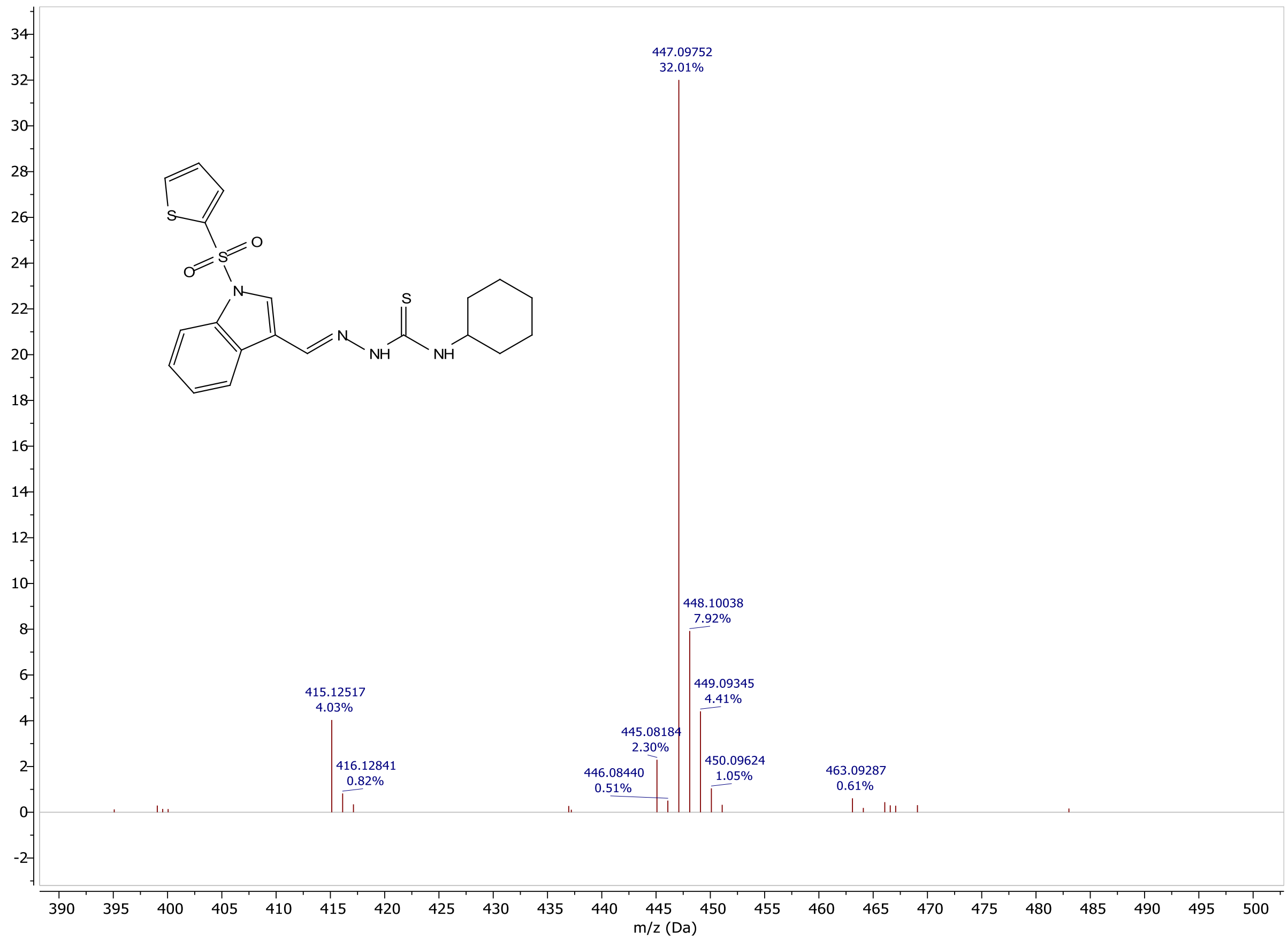

447.09752
32.01%
448.10038
7.92%
449.09345
4.41%
415.12517
4.03%
445.08184
2.30%
416.12841
0.82%
446.08440
0.51%
450.09624
1.05%
463.09287
0.61%
m/z (Da)

^{1}H & ^{13}C NMR Spectra of 5i

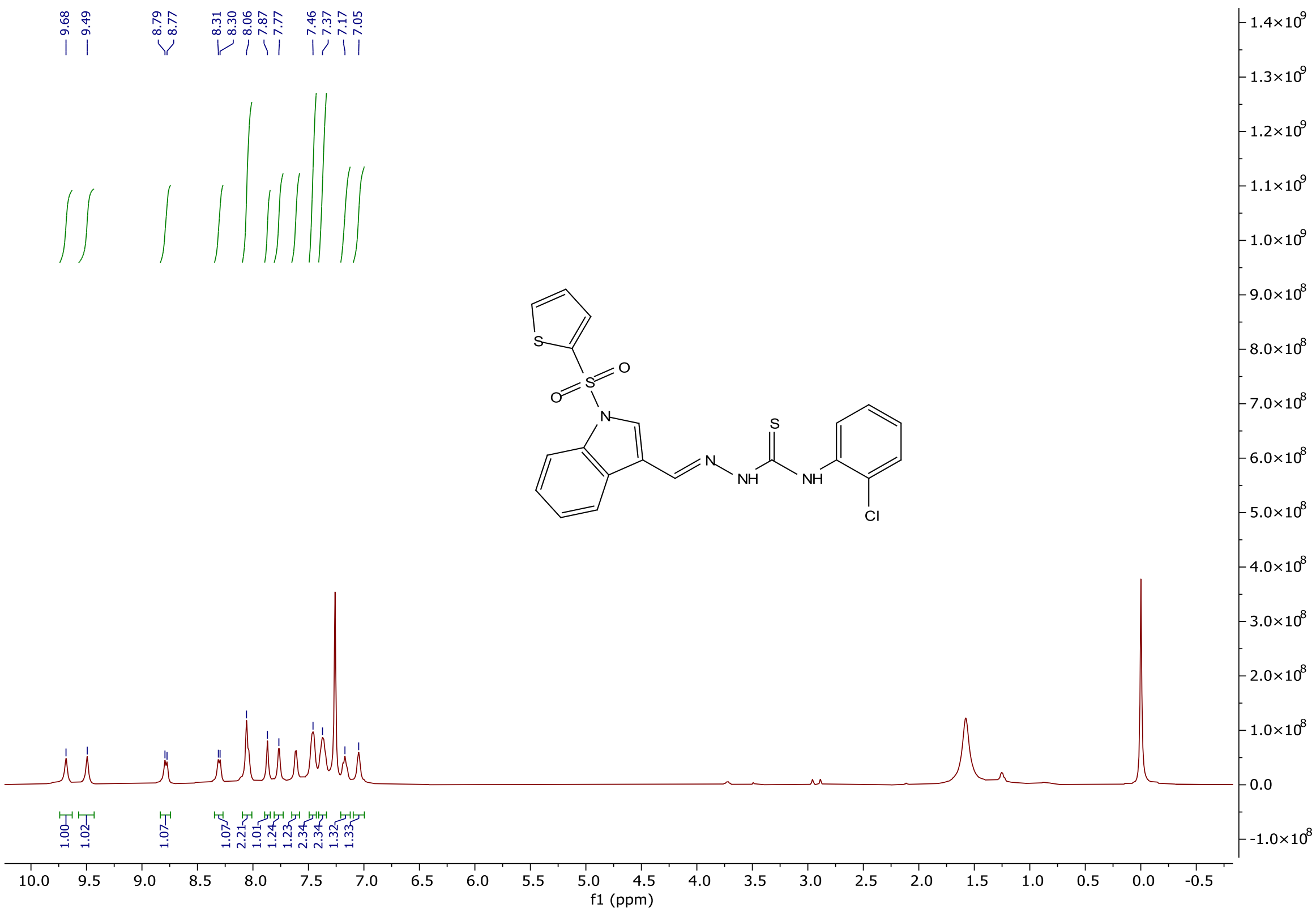

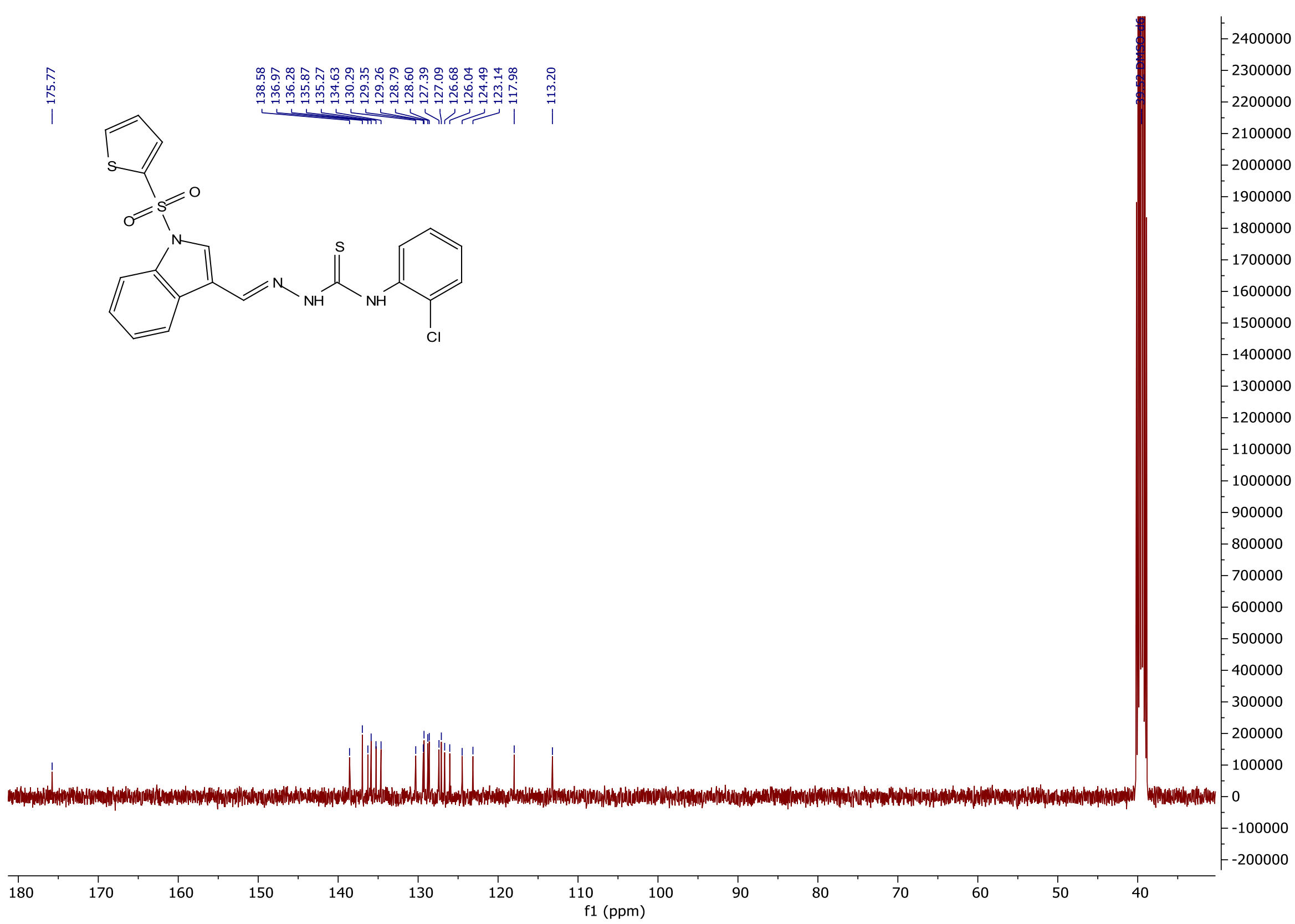

HRMS of 5i

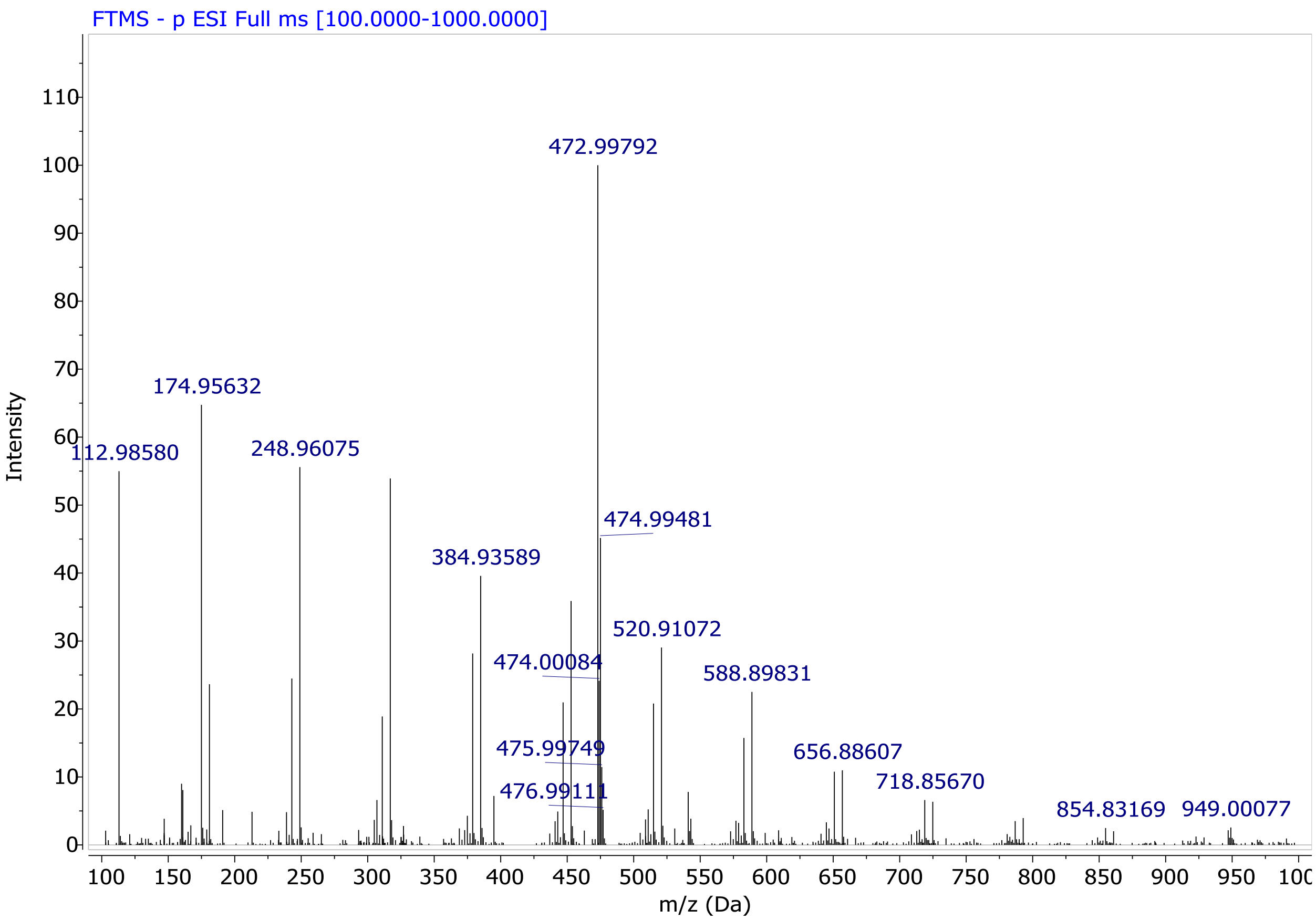

FTMS - p ESI Full ms [100.0000-1000.0000]
Intensity
m/z (Da)
472.99792
174.95632
112.98580
248.96075
474.99481
384.93589
520.91072
474.00084
588.89831
475.99749
656.88607
476.99111
718.85670
854.83169
949.00077

^{1}H & ^{13}C NMR Spectra of 5j

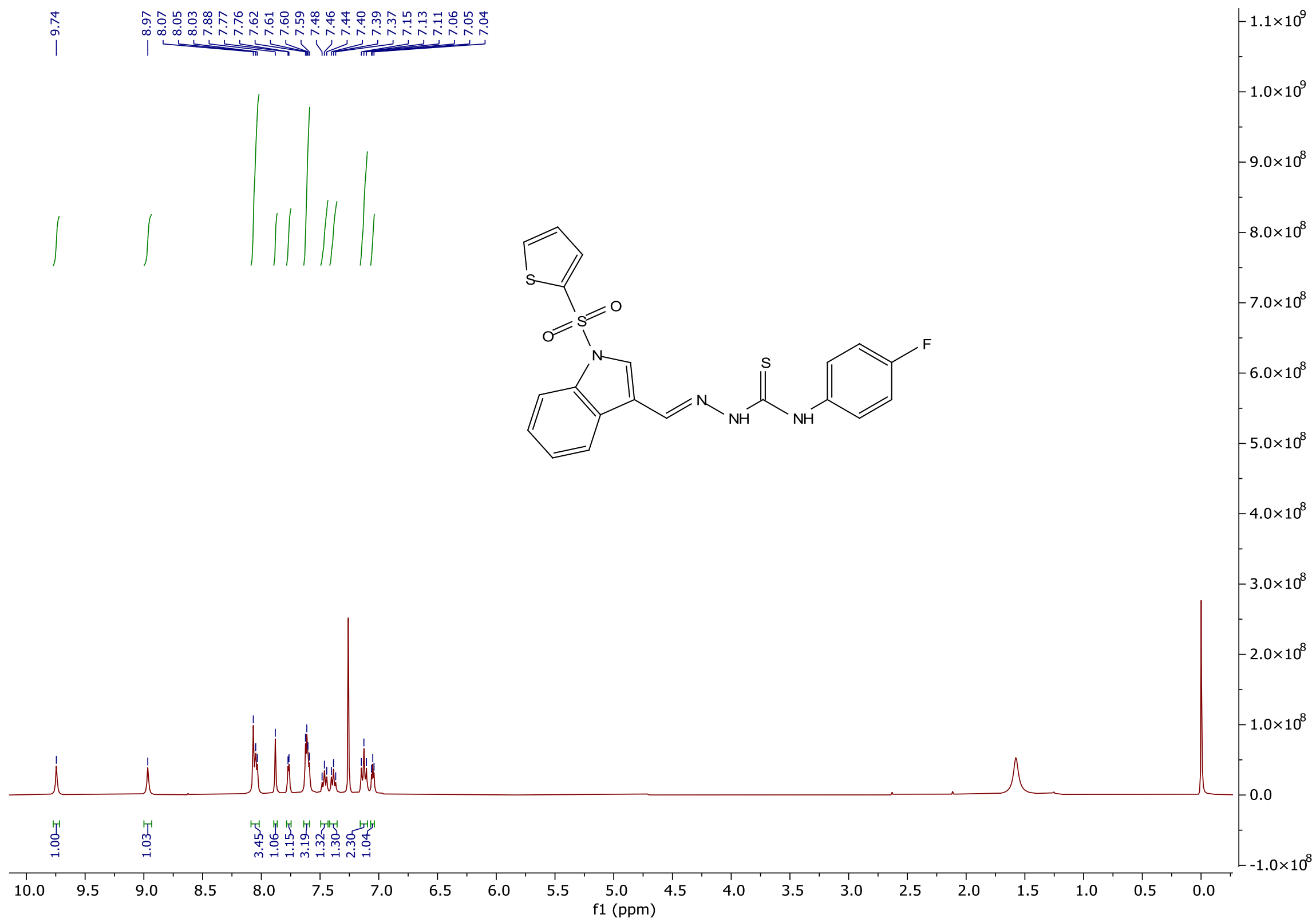

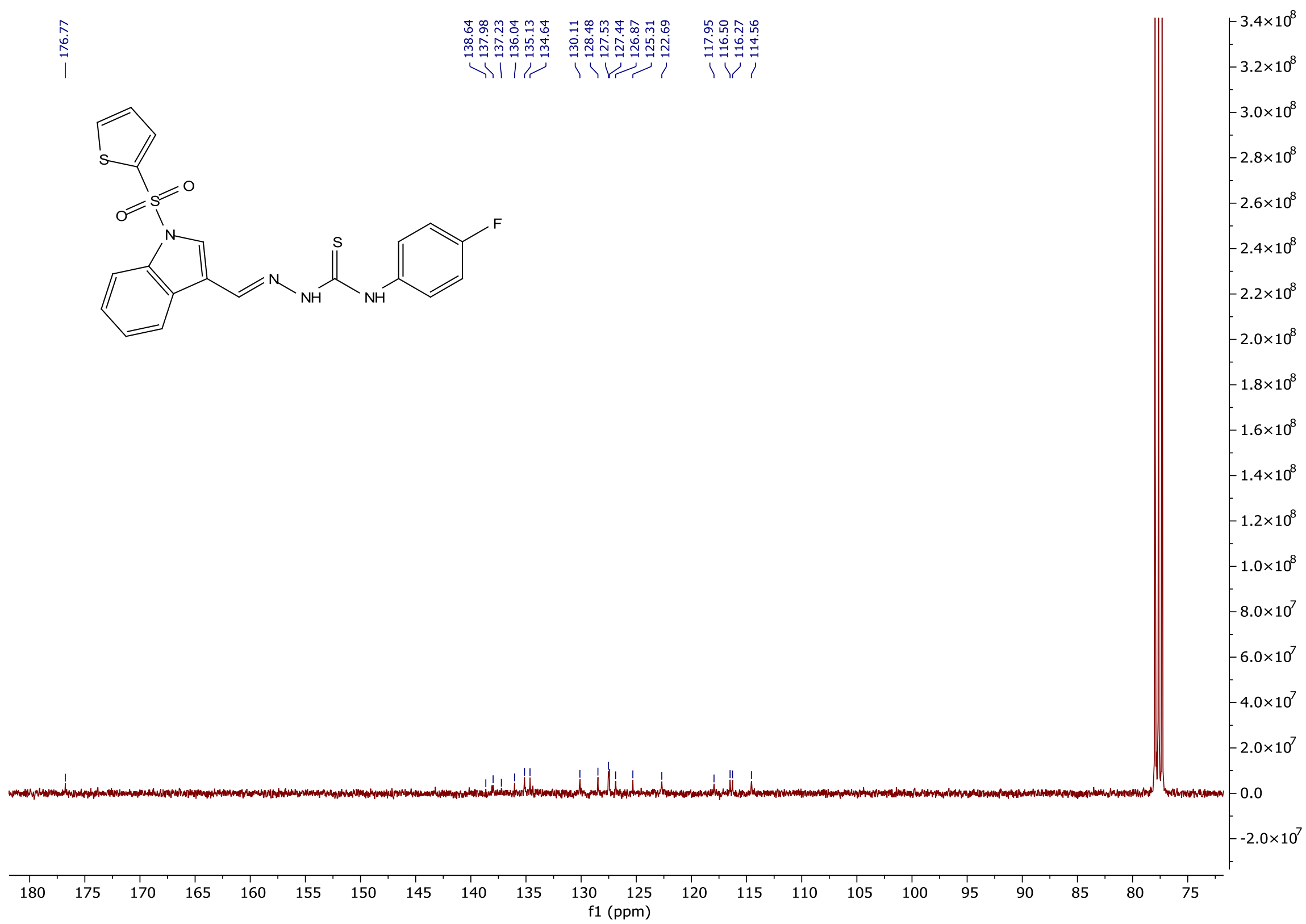

HRMS of 5j

19F NMR of 5j

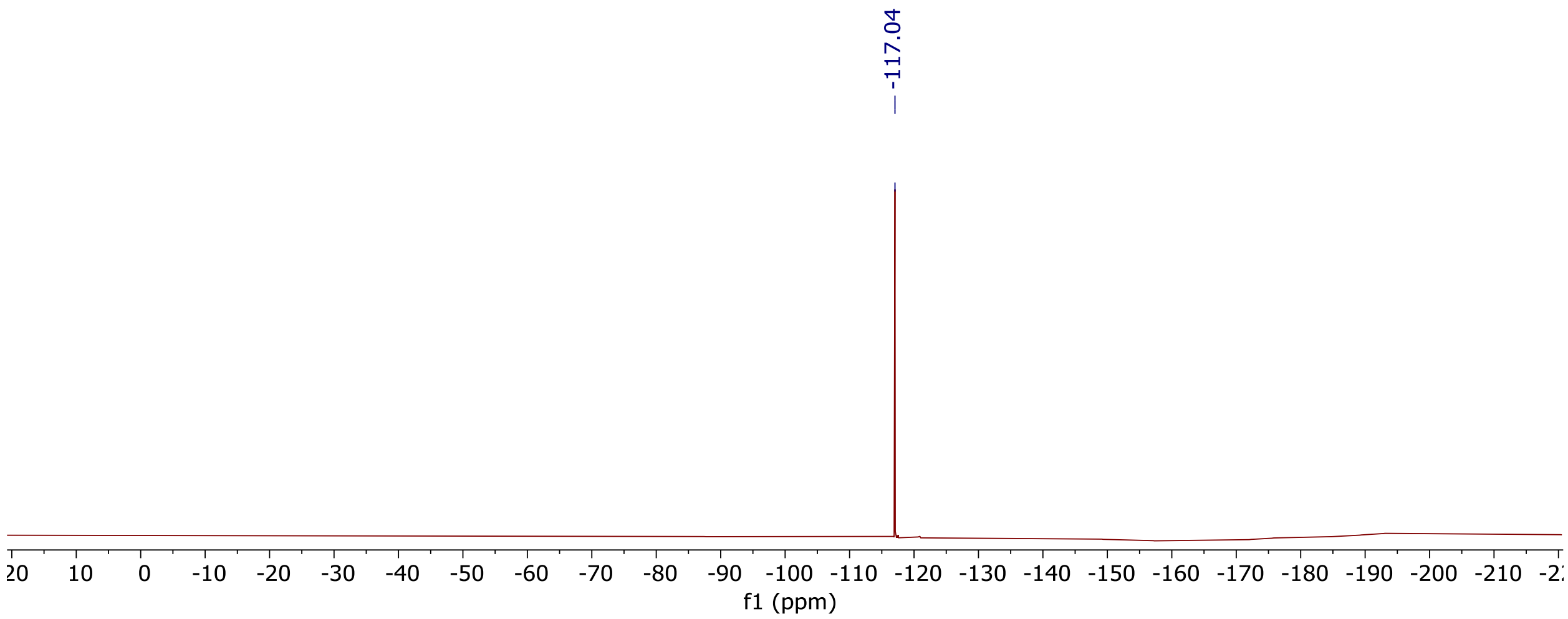

HRMS of 5j

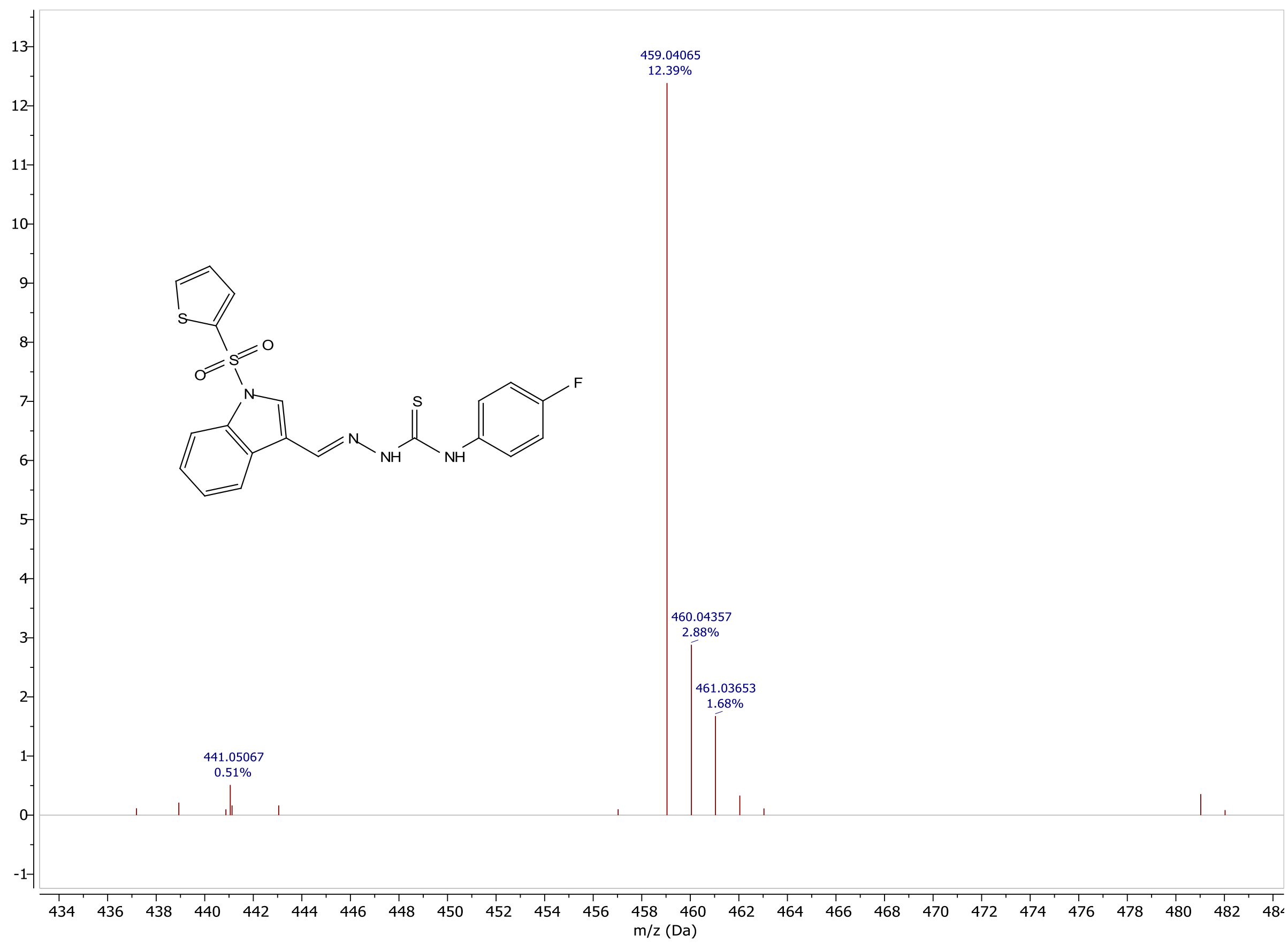

^{1}H & ^{13}C NMR Spectra of 5k

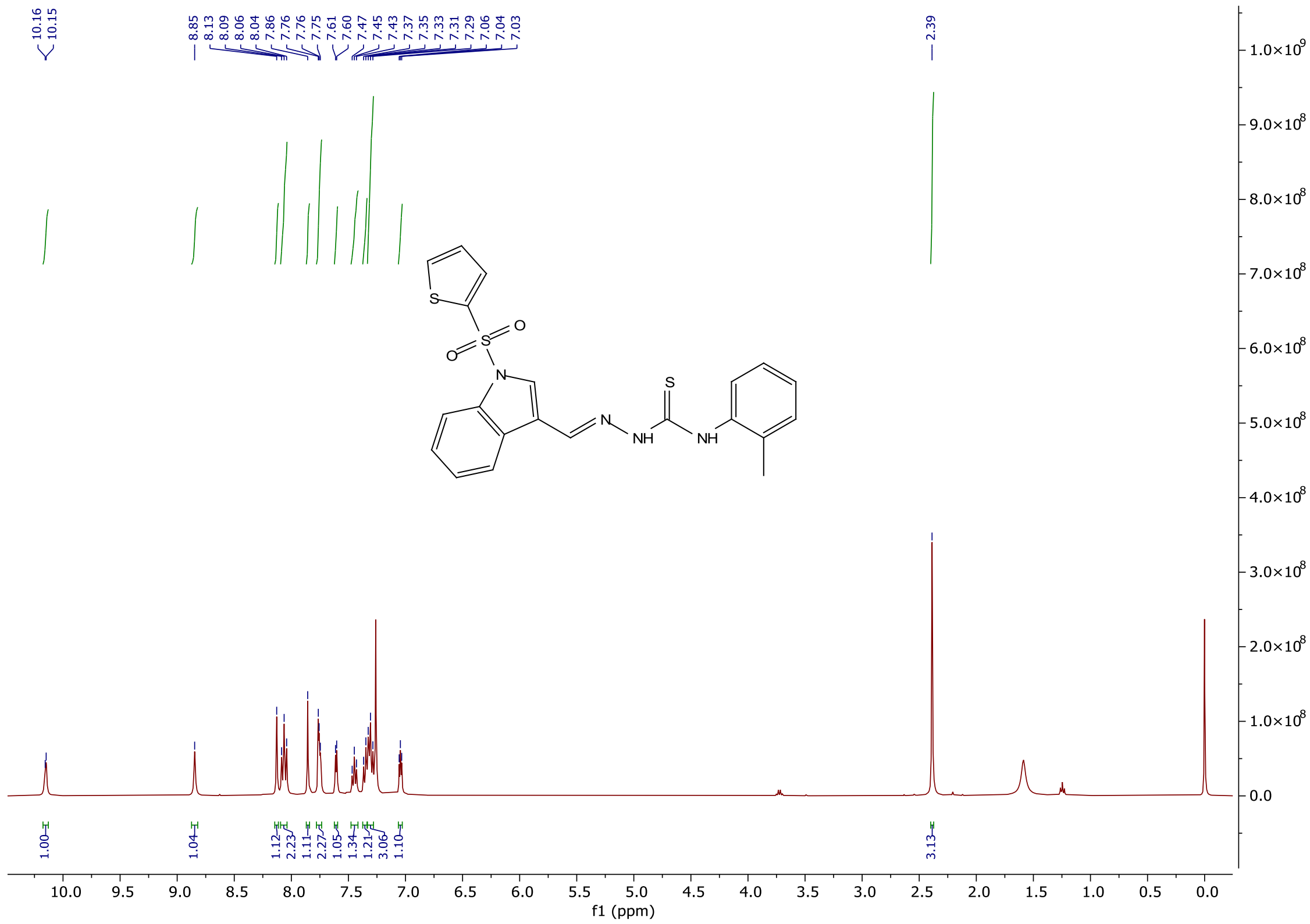

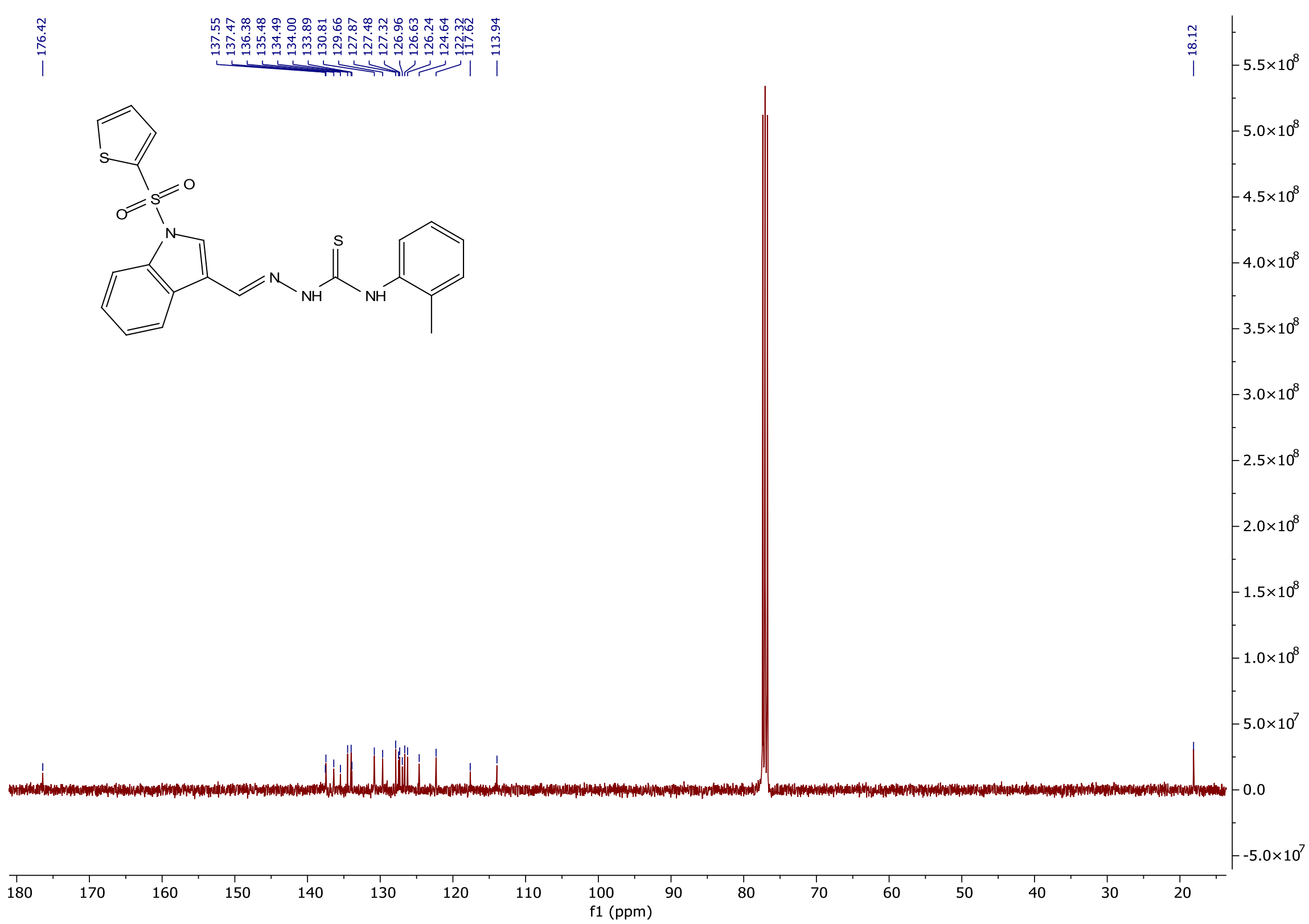

HRMS of 5k

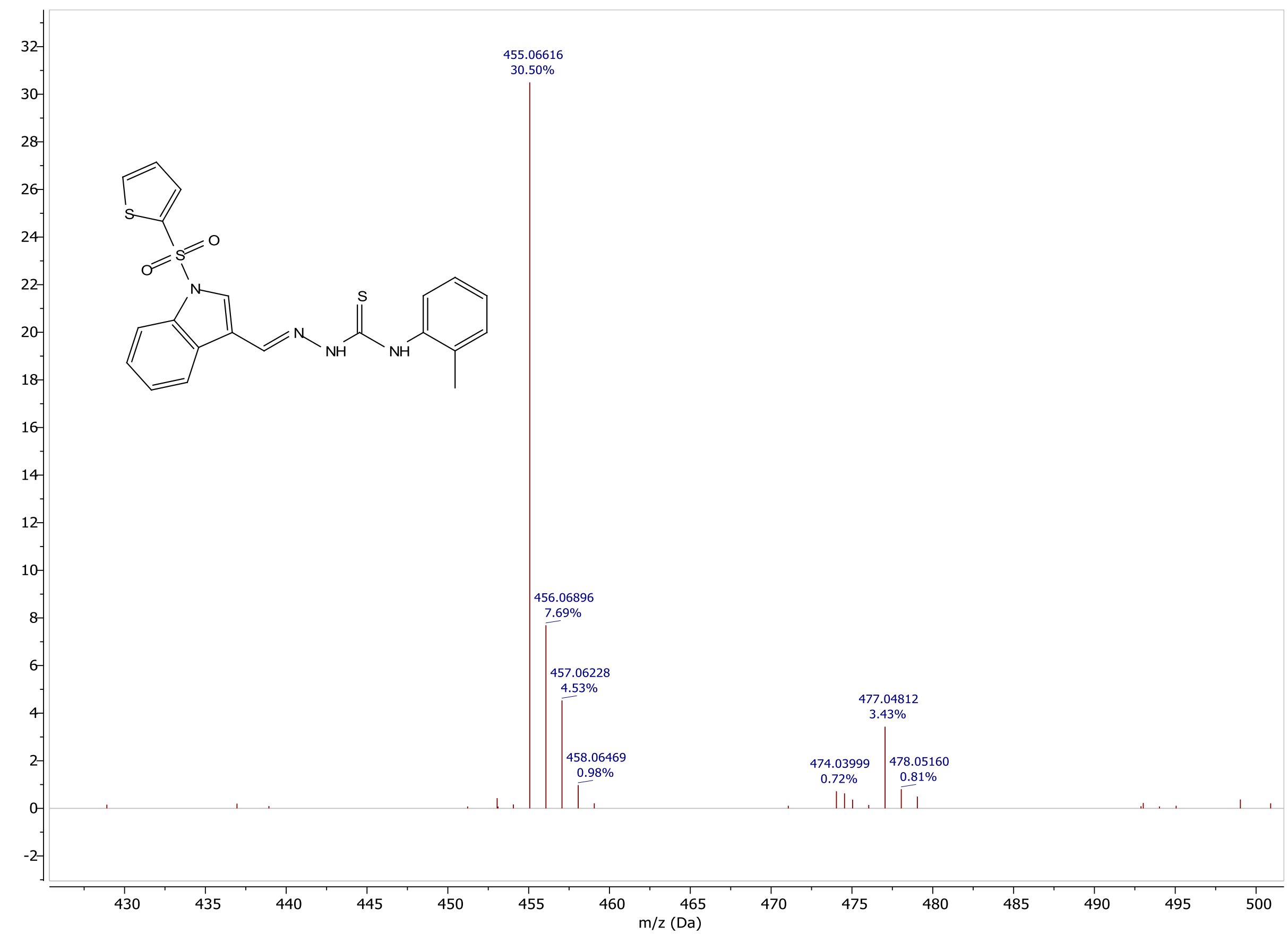

455.06616
30.50%
456.06896
7.69%
457.06228
4.53%
458.06469
0.98%
474.03999
0.72%
477.04812
3.43%
478.05160
0.81%
S
O
S
O
N
N
NH
S
NH
m/z (Da)

^{1}H & ^{13}C NMR Spectra of 5l

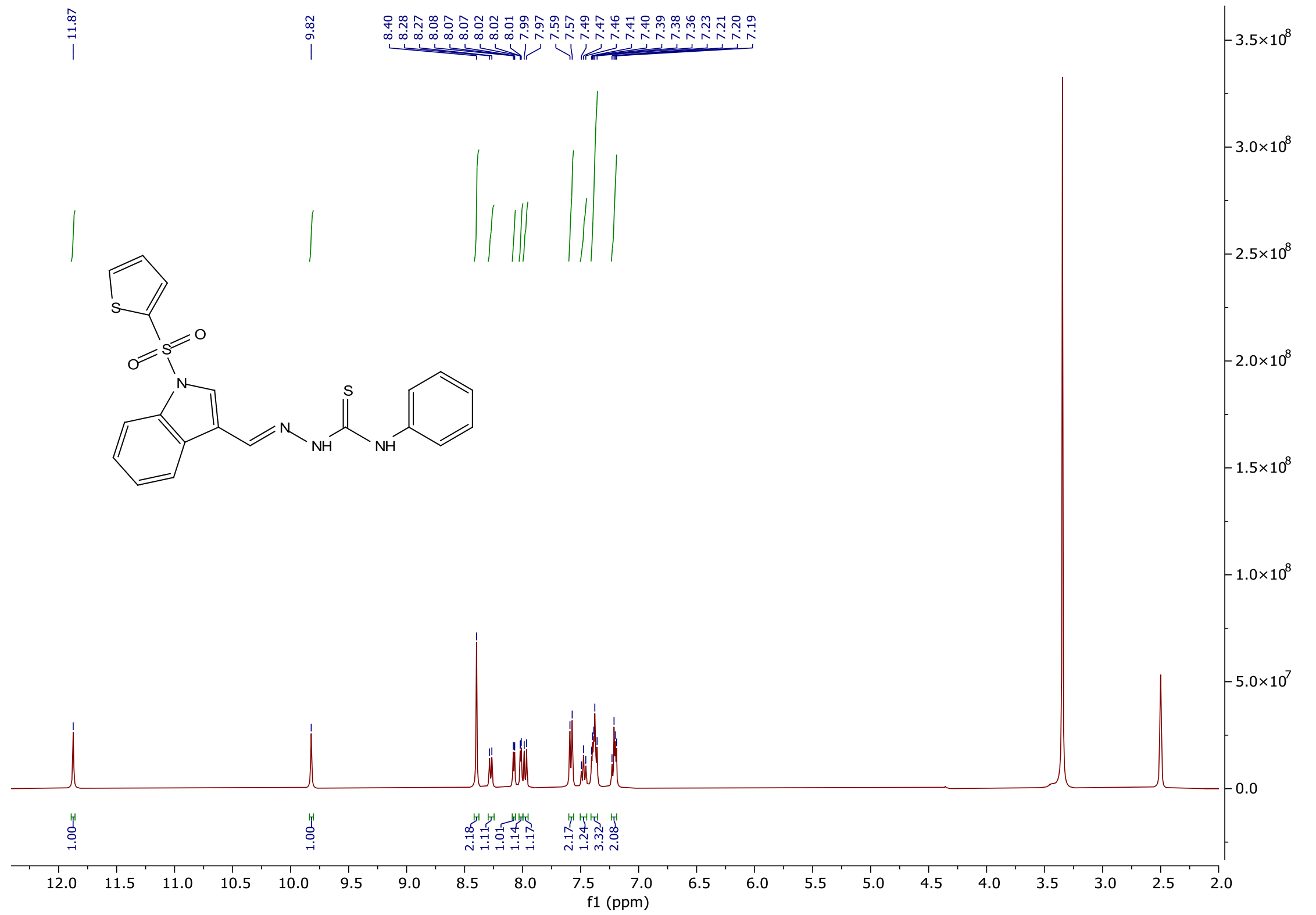

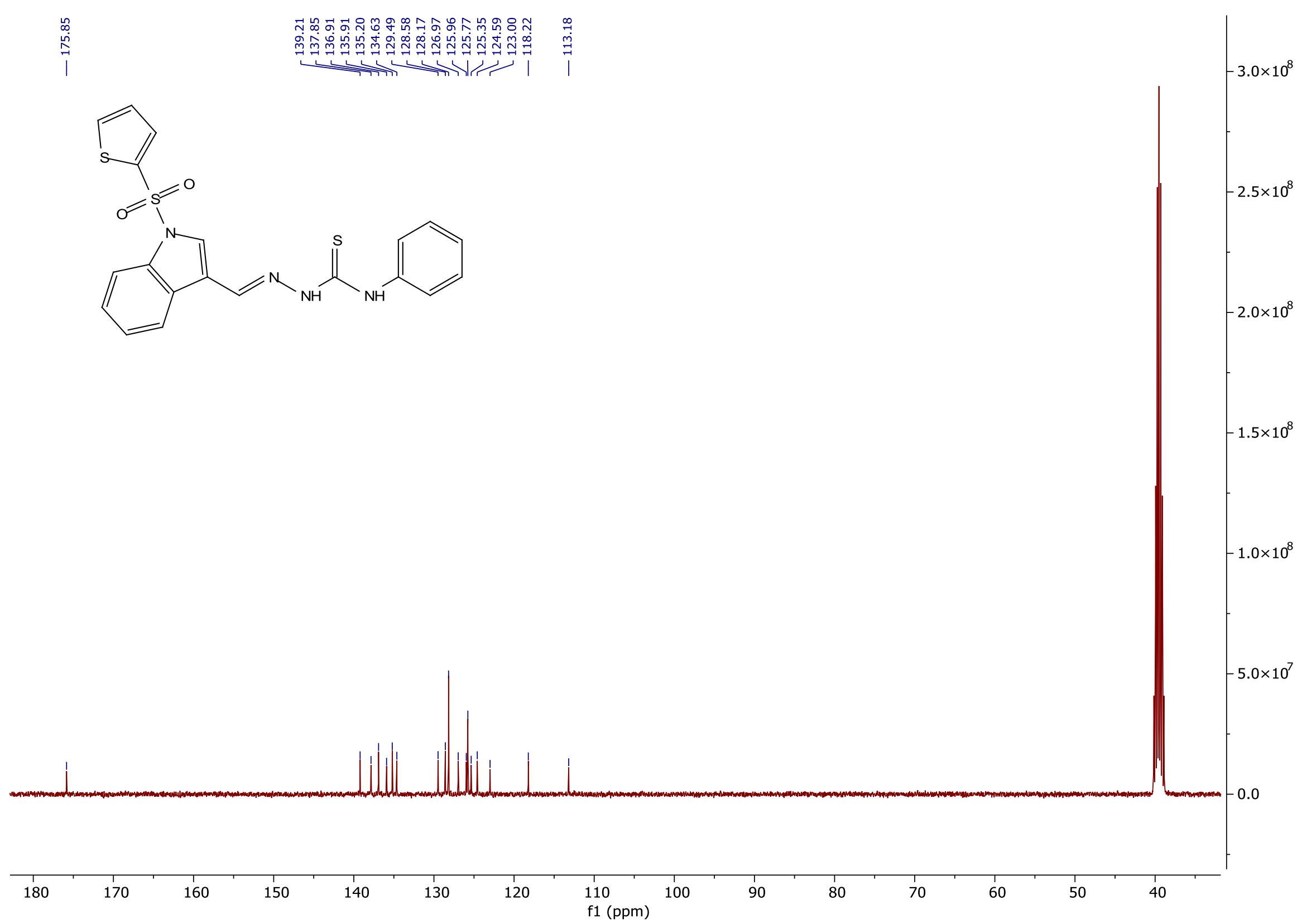

HRMS of 5l

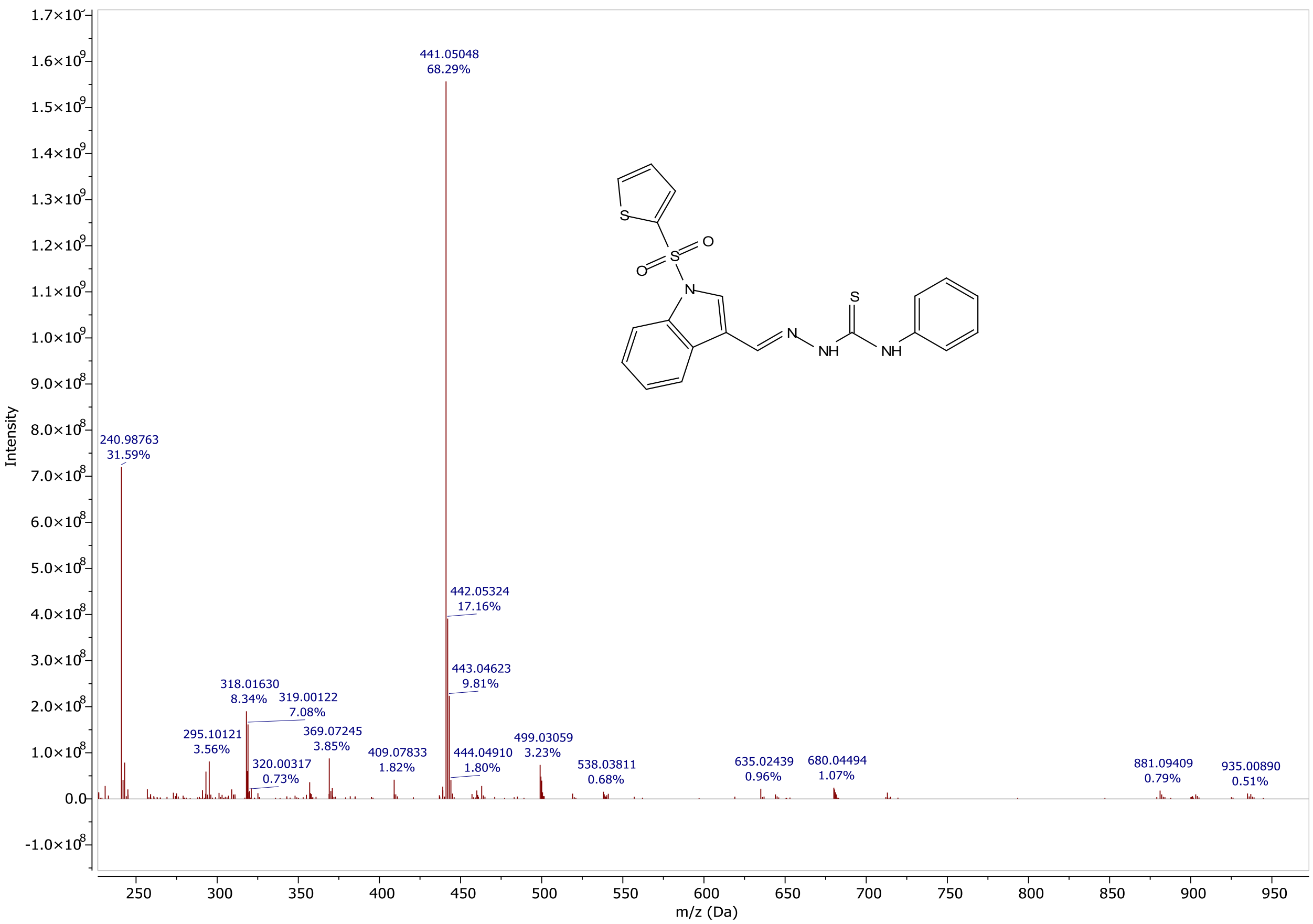

Intensity
m/z (Da)
441.05048
68.29%
240.98763
31.59%
442.05324
17.16%
443.04623
9.81%
318.01630
8.34%
319.00122
7.08%
295.10121
3.56%
369.07245
3.85%
320.00317
0.73%
409.07833
1.82%
444.04910
1.80%
499.03059
3.23%
538.03811
0.68%
635.02439
0.96%
680.04494
1.07%
881.09409
0.79%
935.00890
0.51%

^{1}H & ^{13}C NMR Spectra of 5m

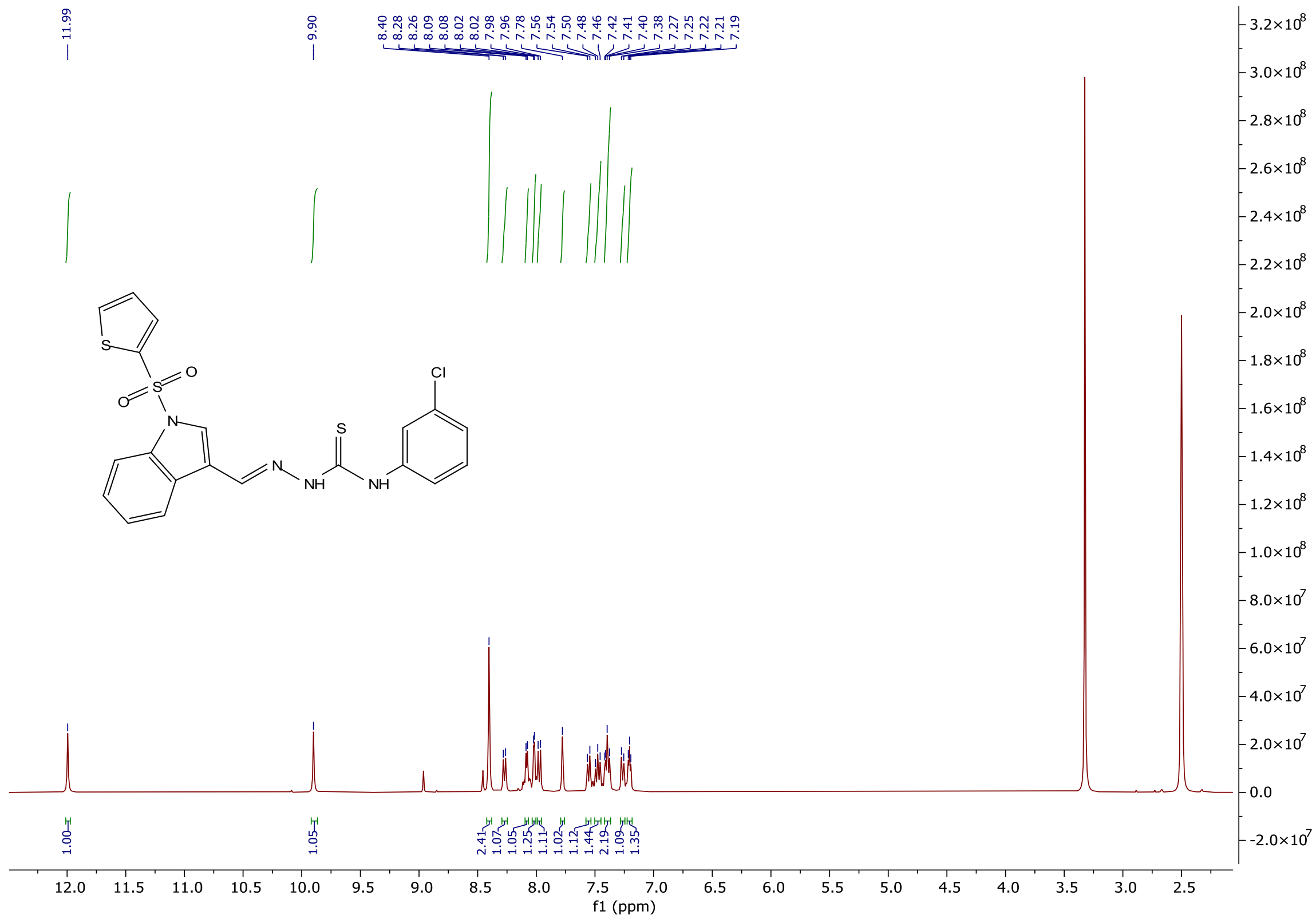

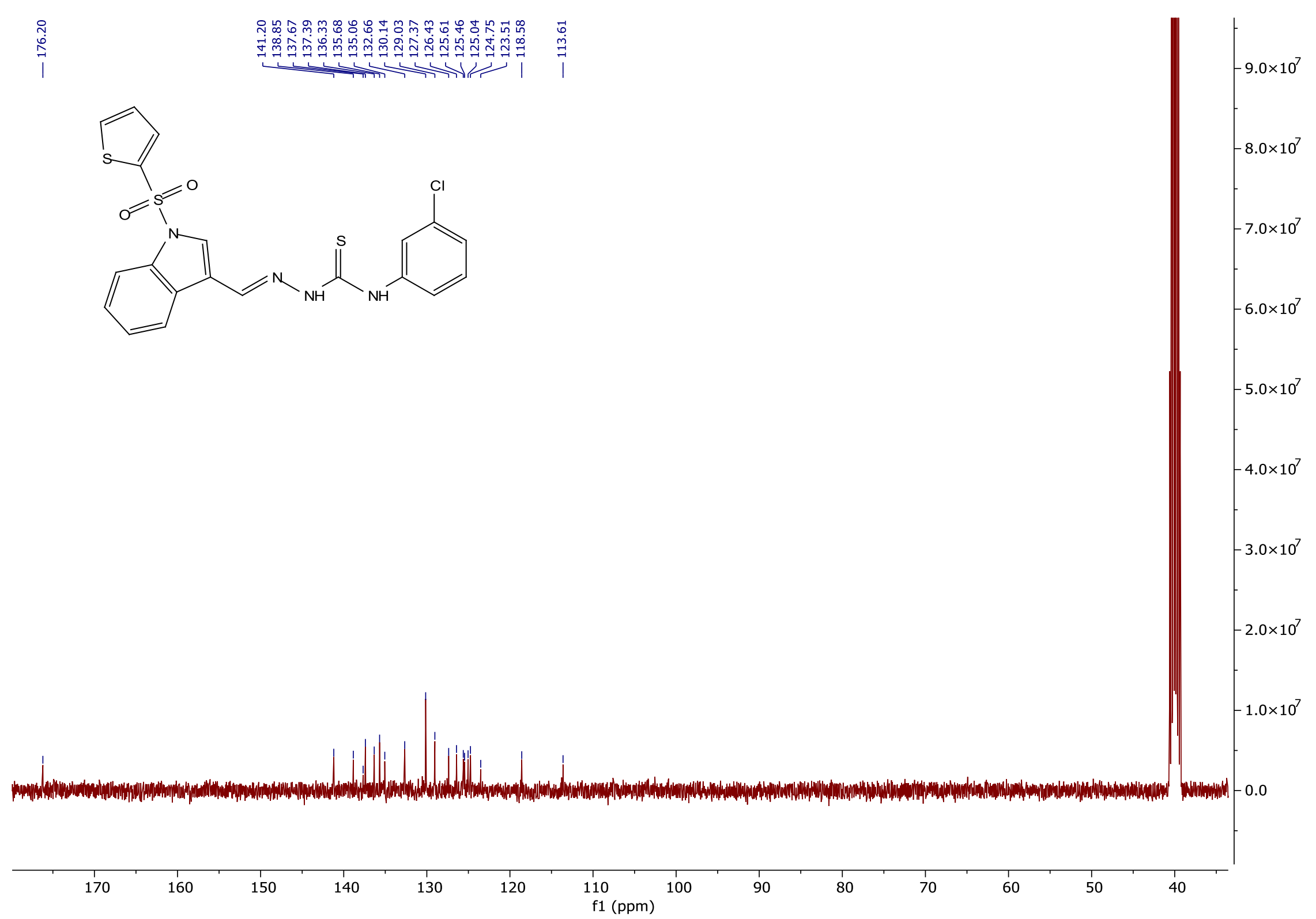

HRMS of 5m

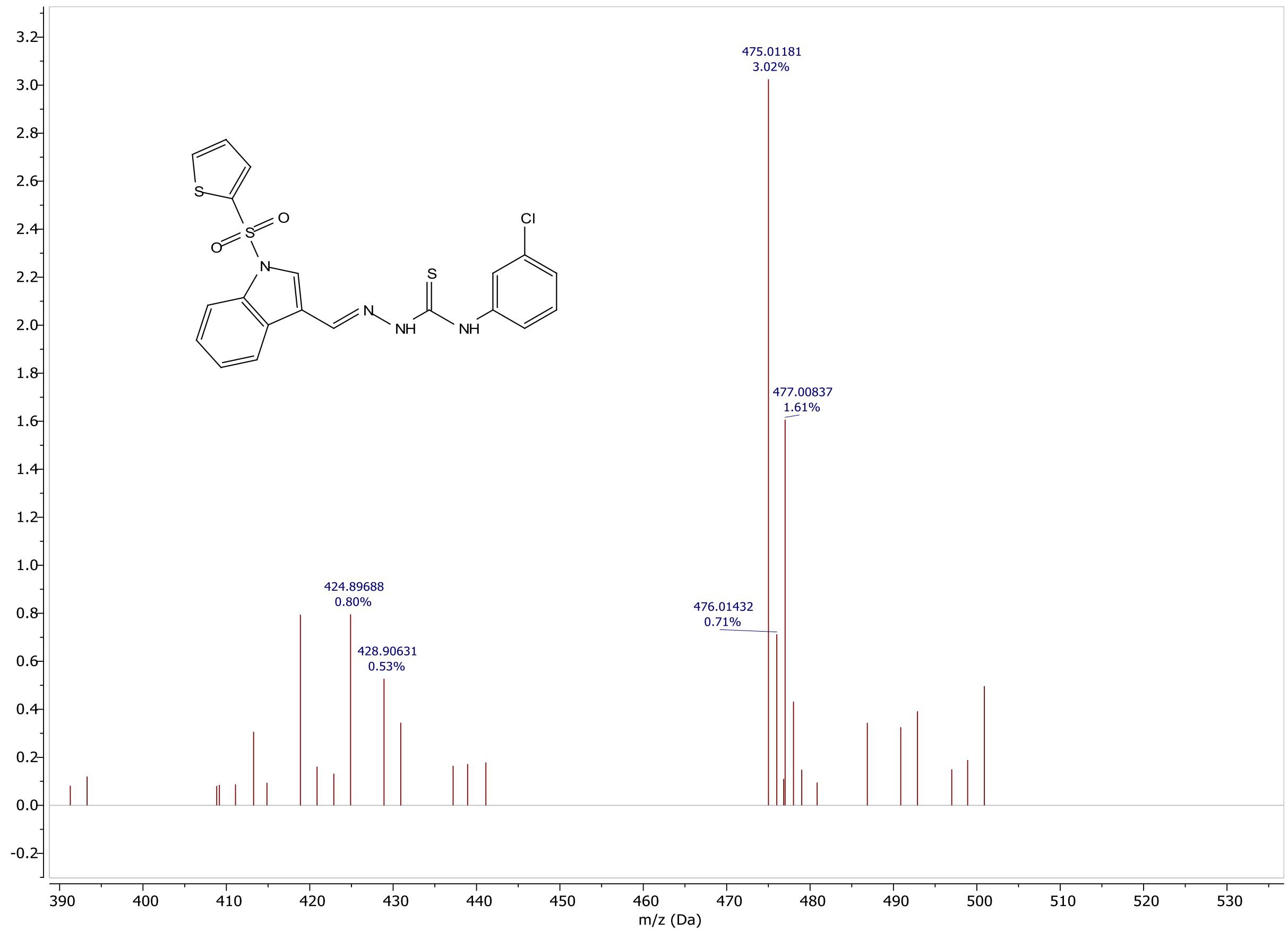

475.01181
3.02%
477.00837
1.61%
424.89688
0.80%
476.01432
0.71%
428.90631
0.53%
Cl
S
O
S
O
N
N
NH
NH
m/z (Da)

^{1}H & ^{13}C NMR Spectra of 5n

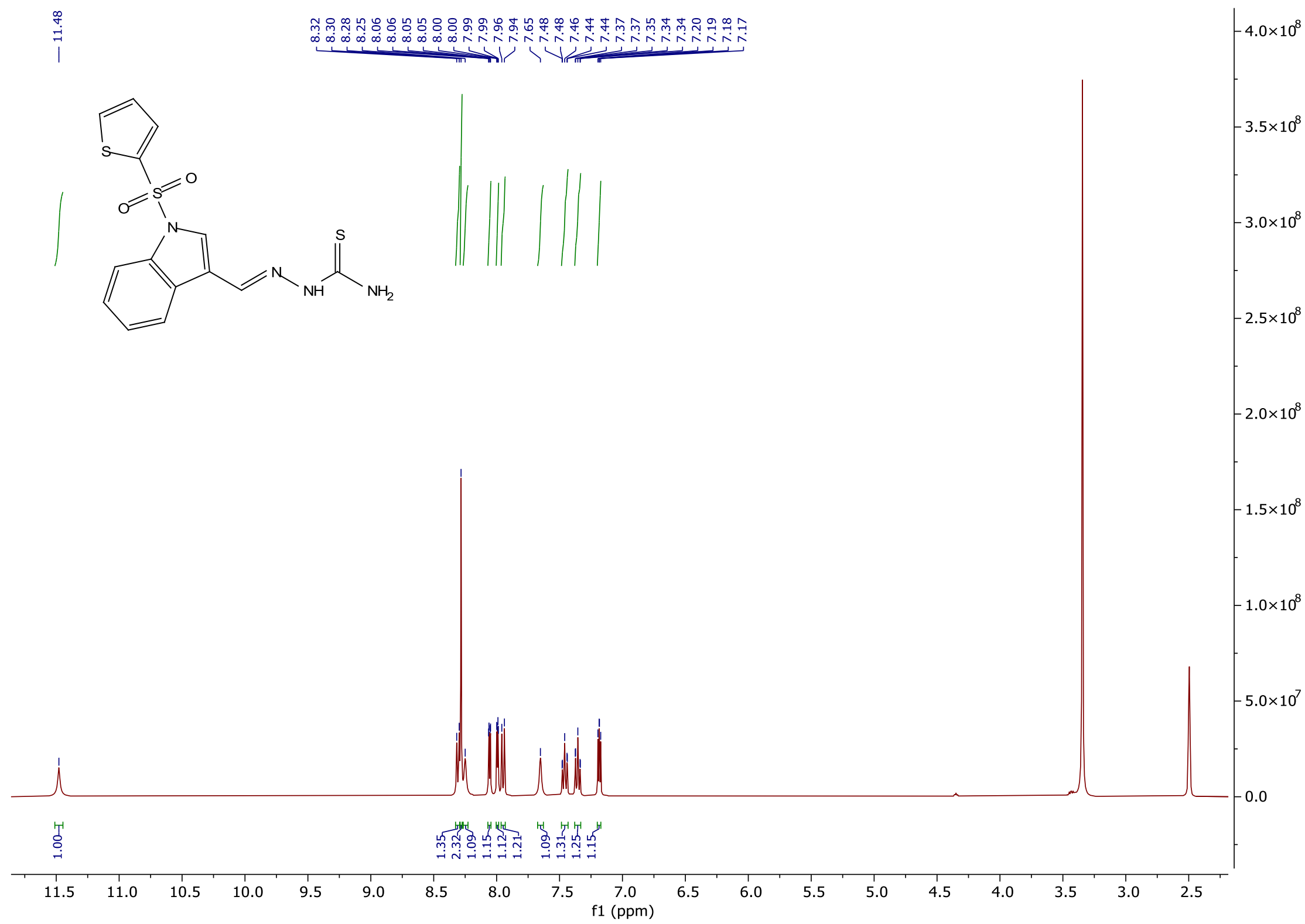

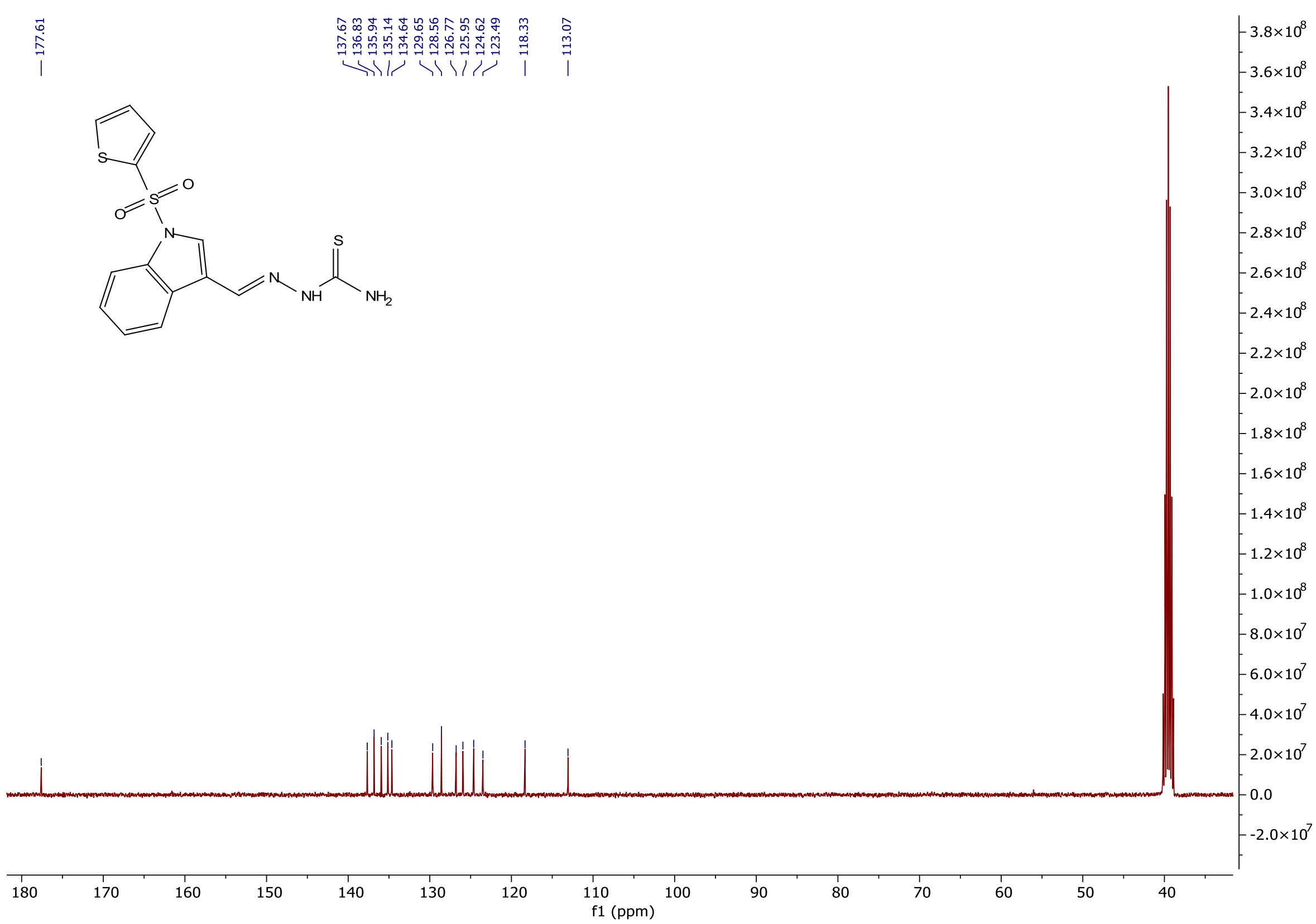

HRMS of 5n

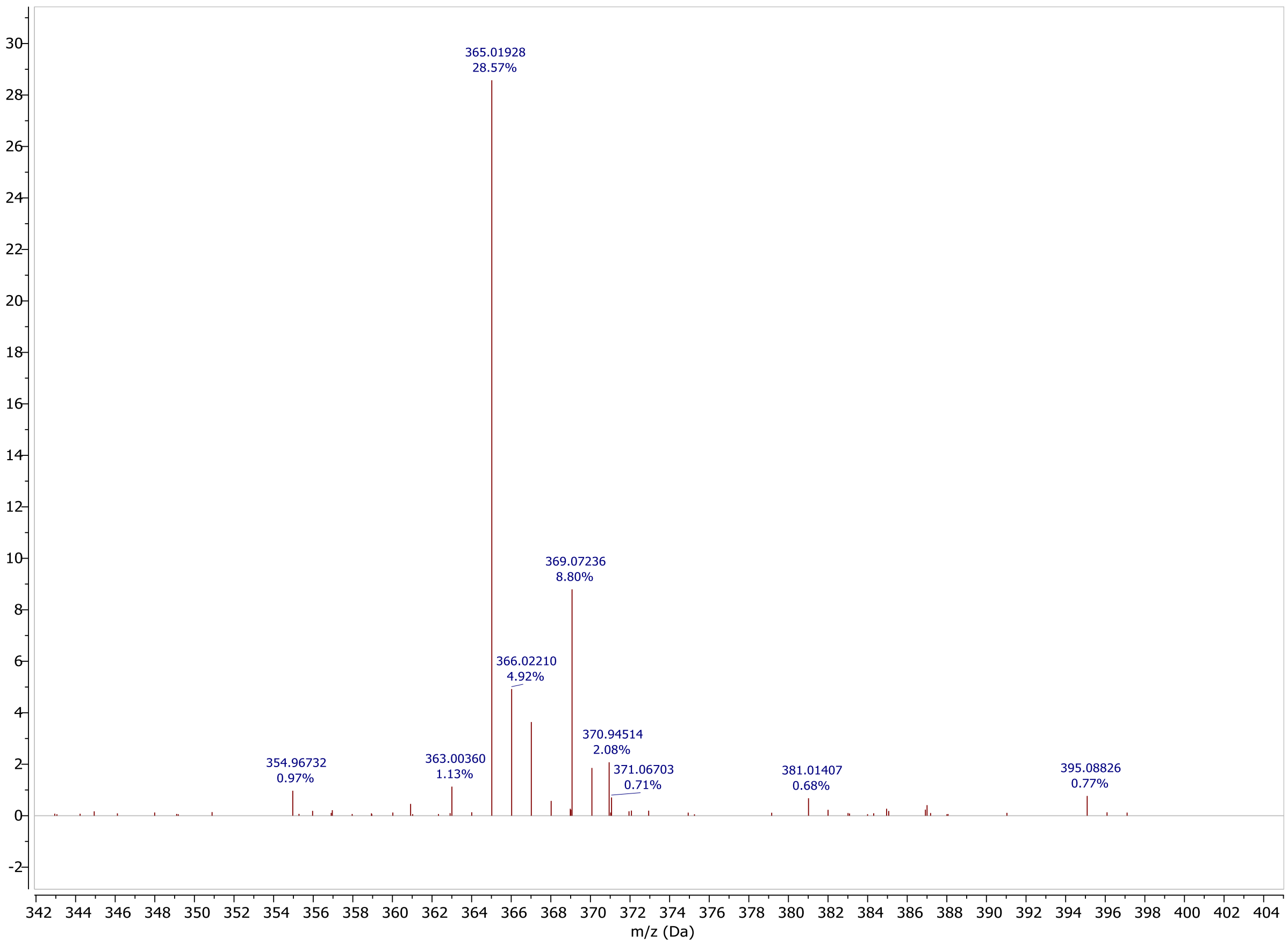

365.01928
28.57%
369.07236
8.80%
366.02210
4.92%
370.94514
2.08%
354.96732
0.97%
363.00360
1.13%
371.06703
0.71%
381.01407
0.68%
395.08826
0.77%
m/z (Da)

^{1}H & ^{13}C NMR Spectra of 5o

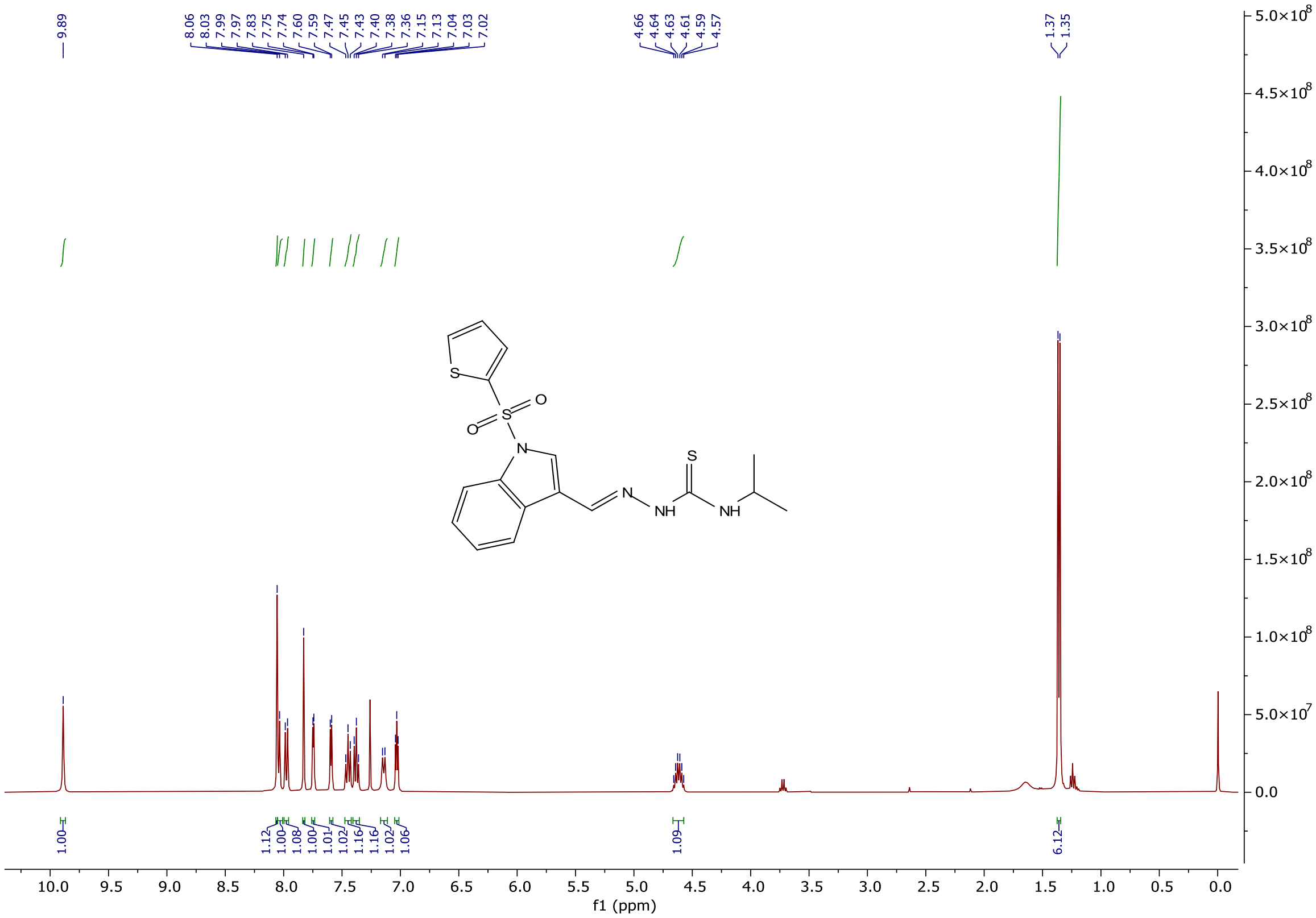

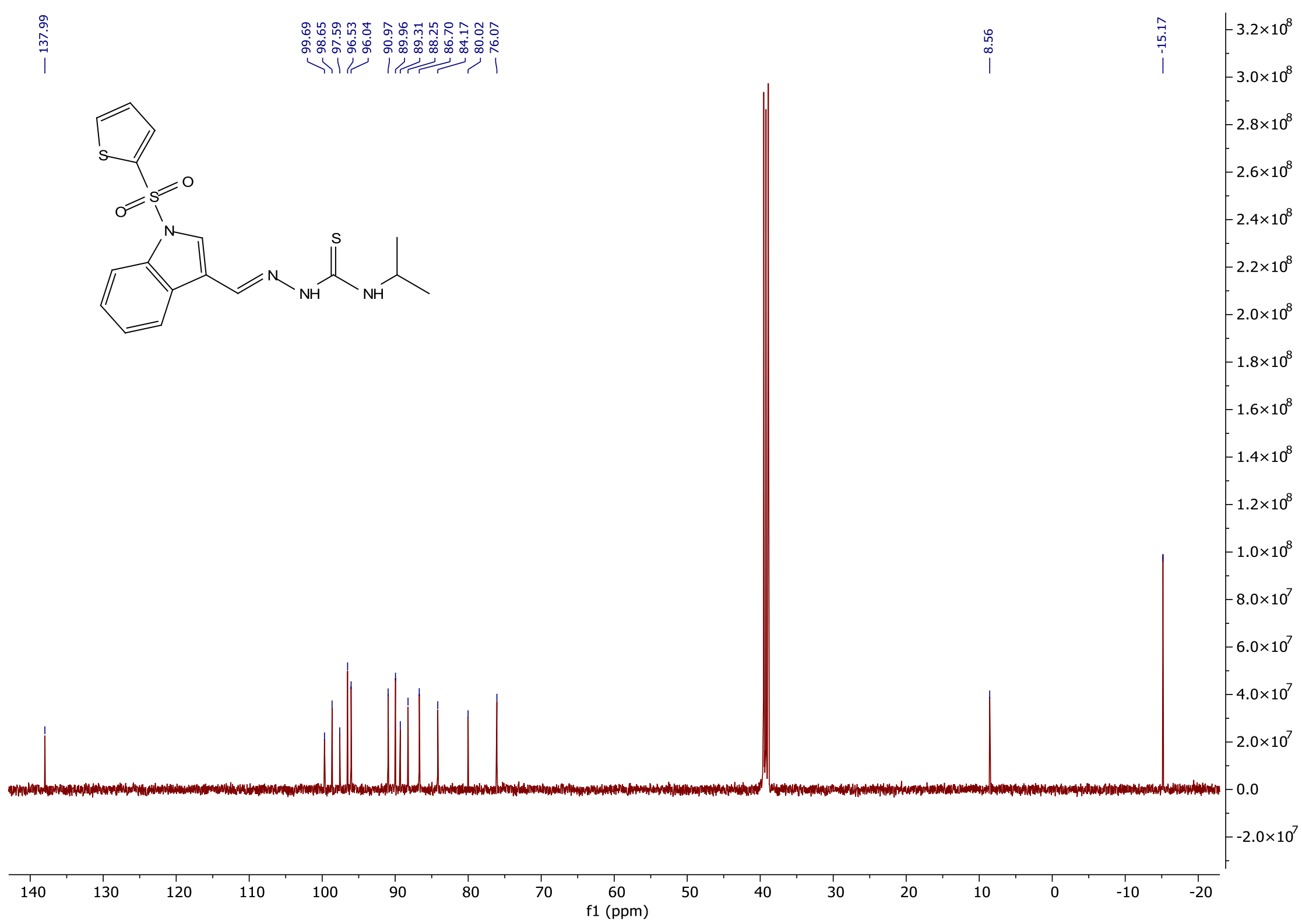

HRMS of 5o

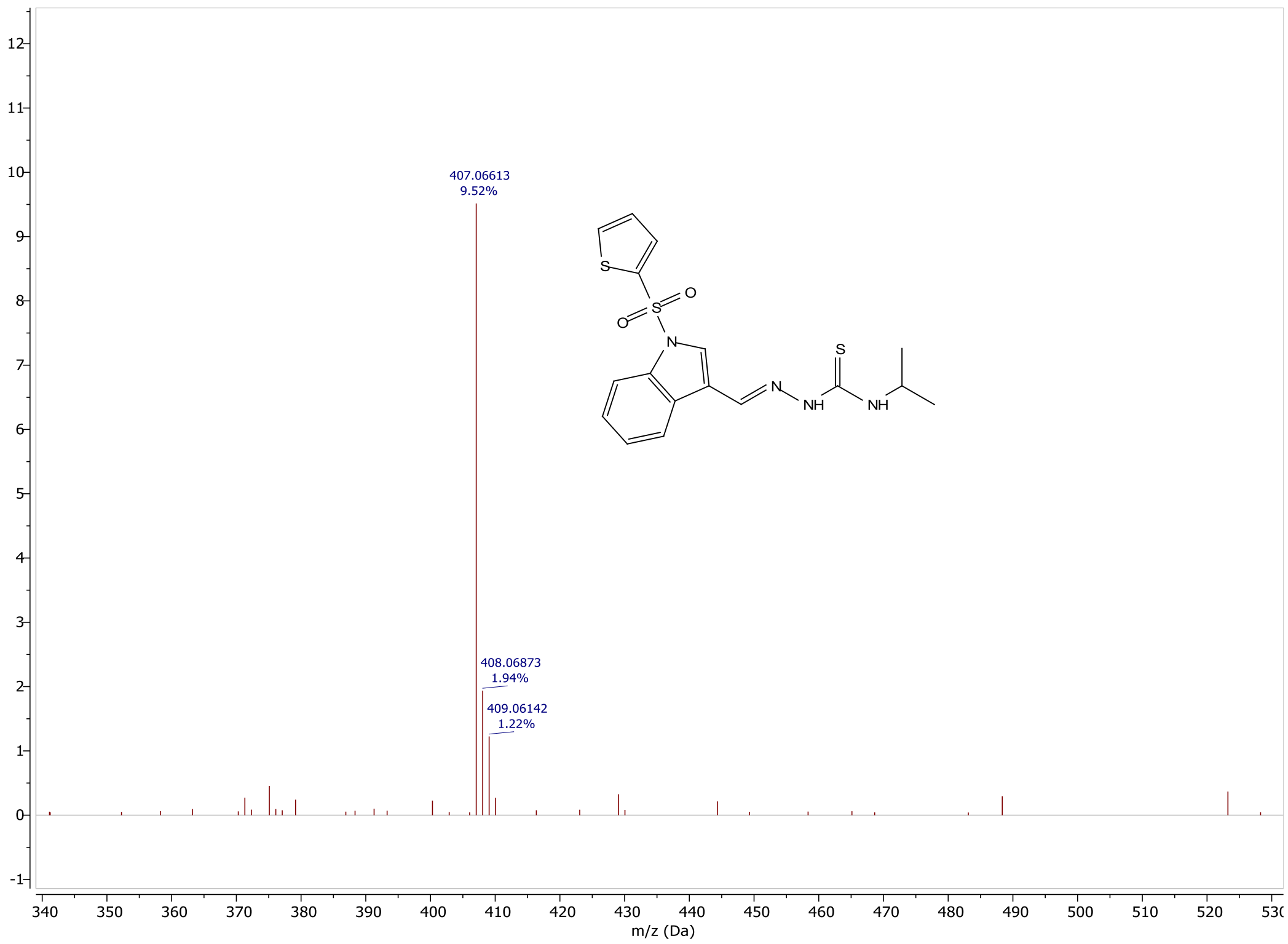

^{1}H & ^{13}C NMR Spectra of 5p

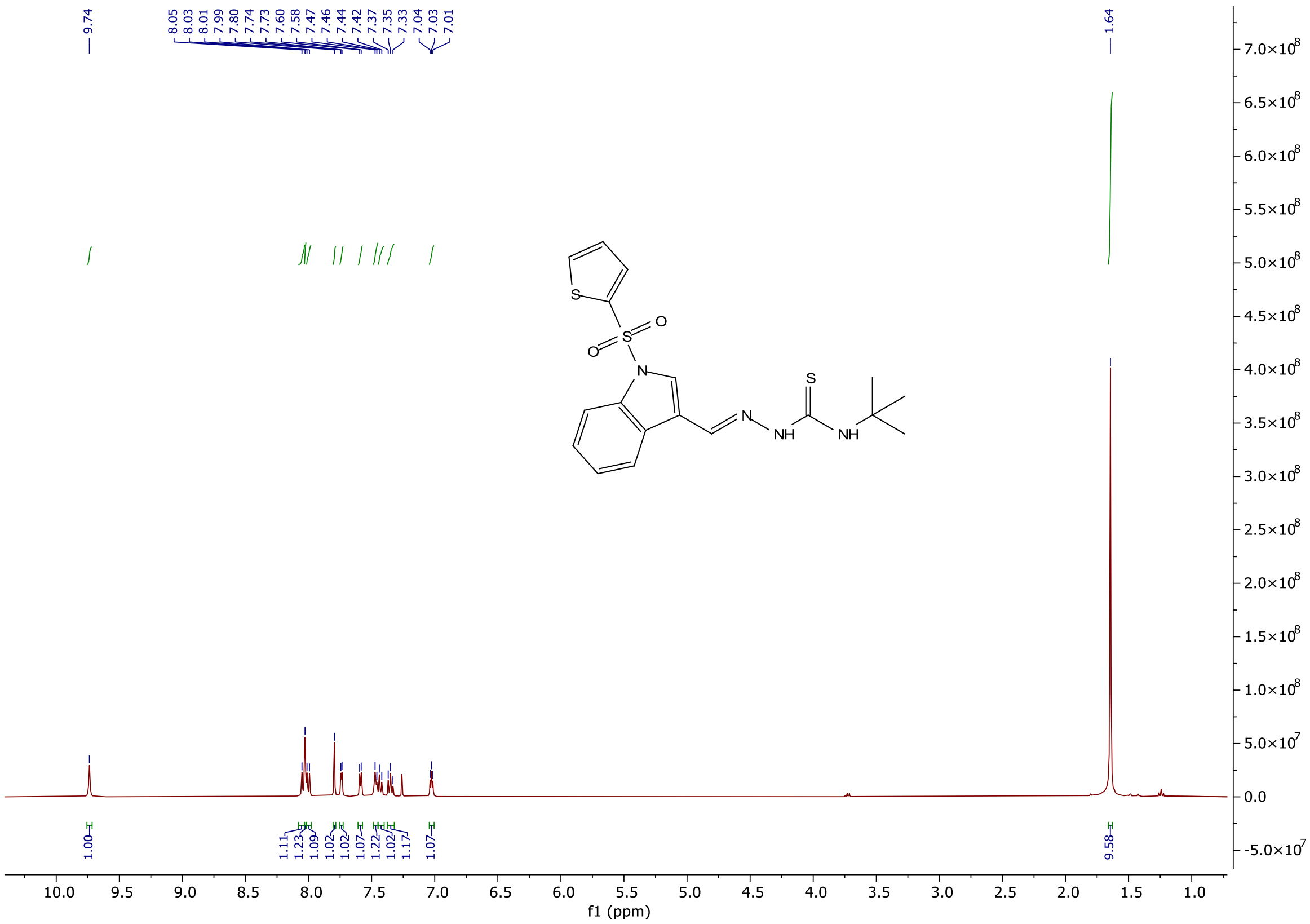

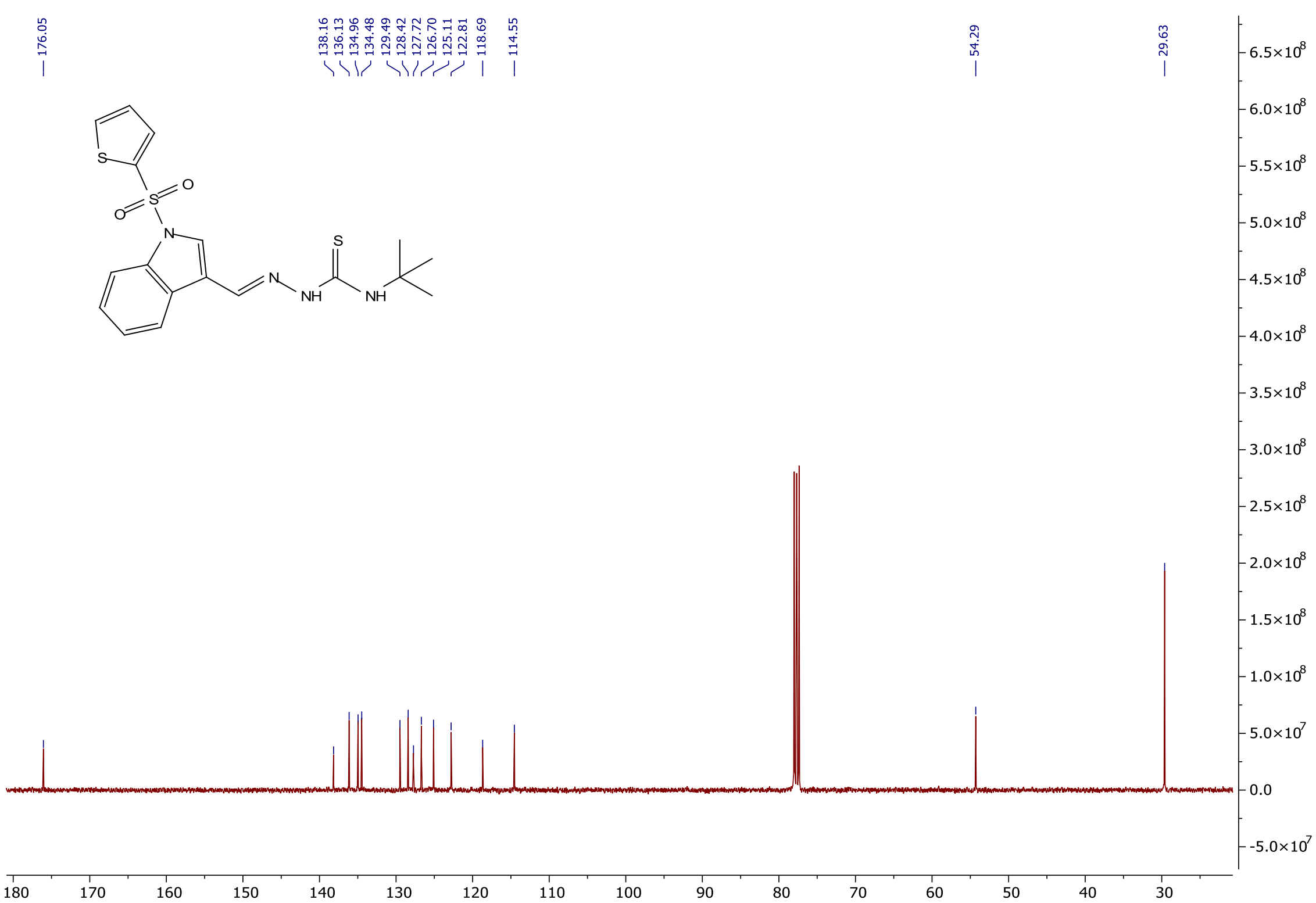

HRMS of 5p

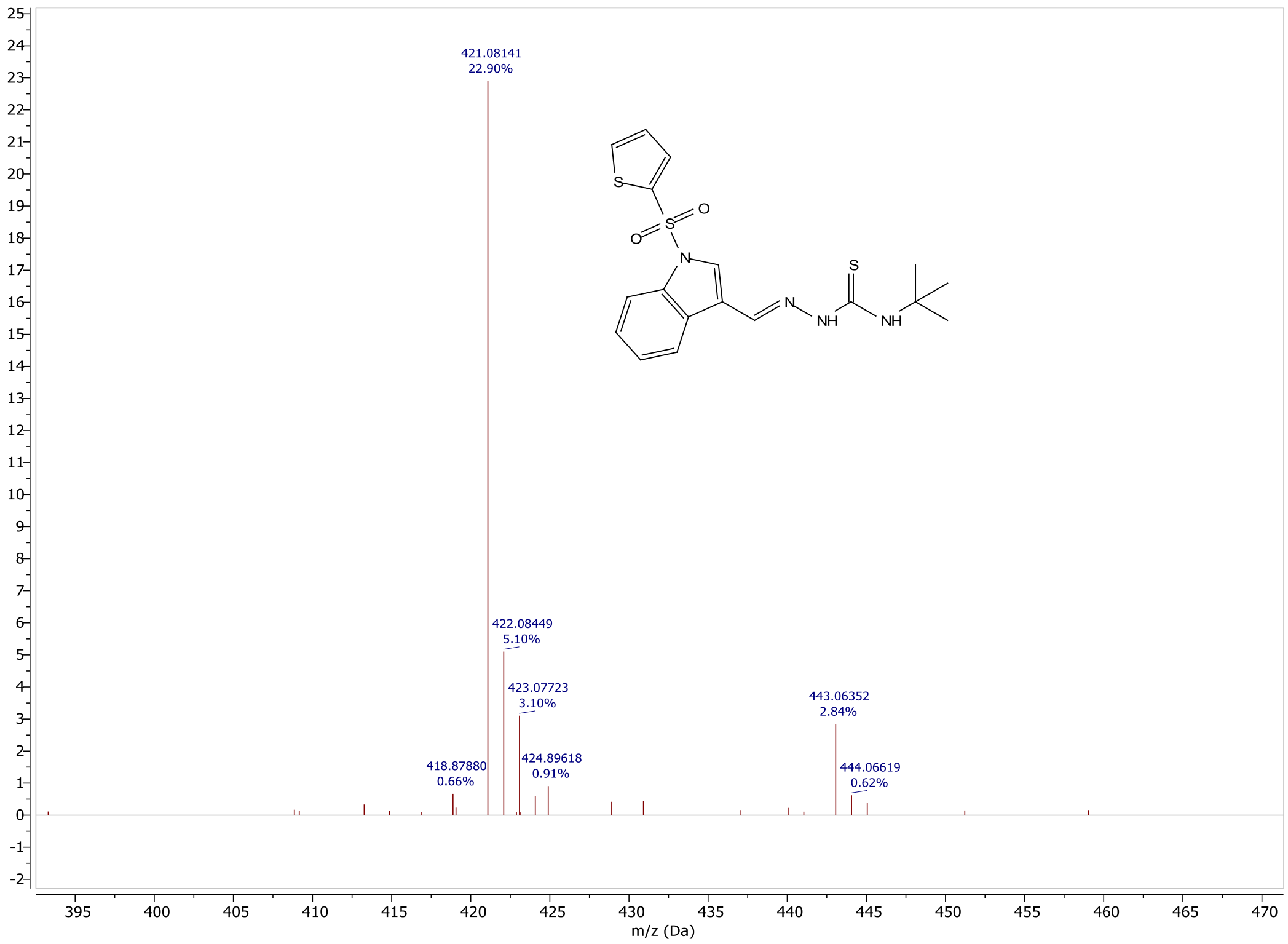

421.08141
22.90%
422.08449
5.10%
423.07723
3.10%
424.89618
0.91%
418.87880
0.66%
443.06352
2.84%
444.06619
0.62%
m/z (Da)

^{1}H & ^{13}C NMR Spectra of 5q

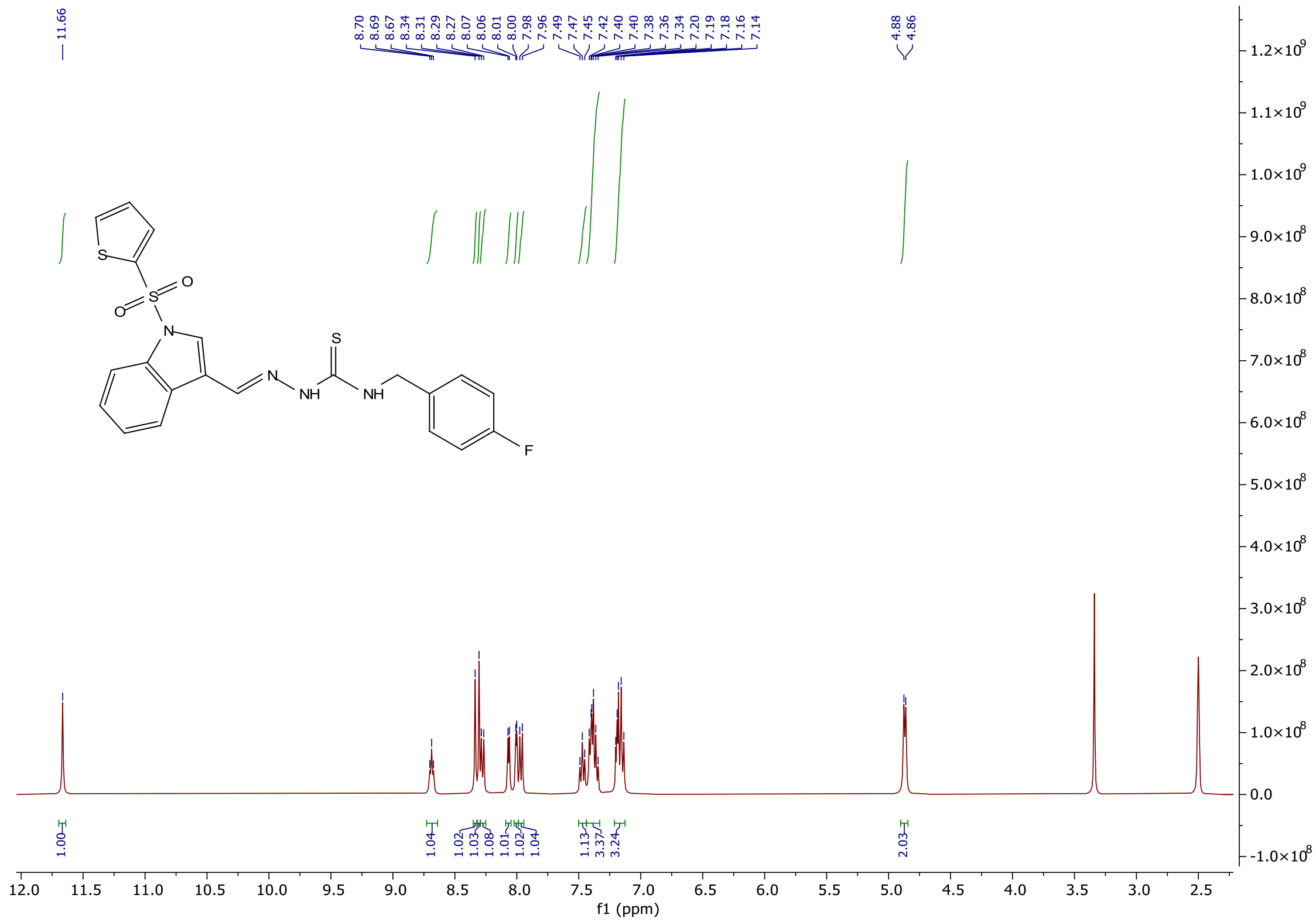

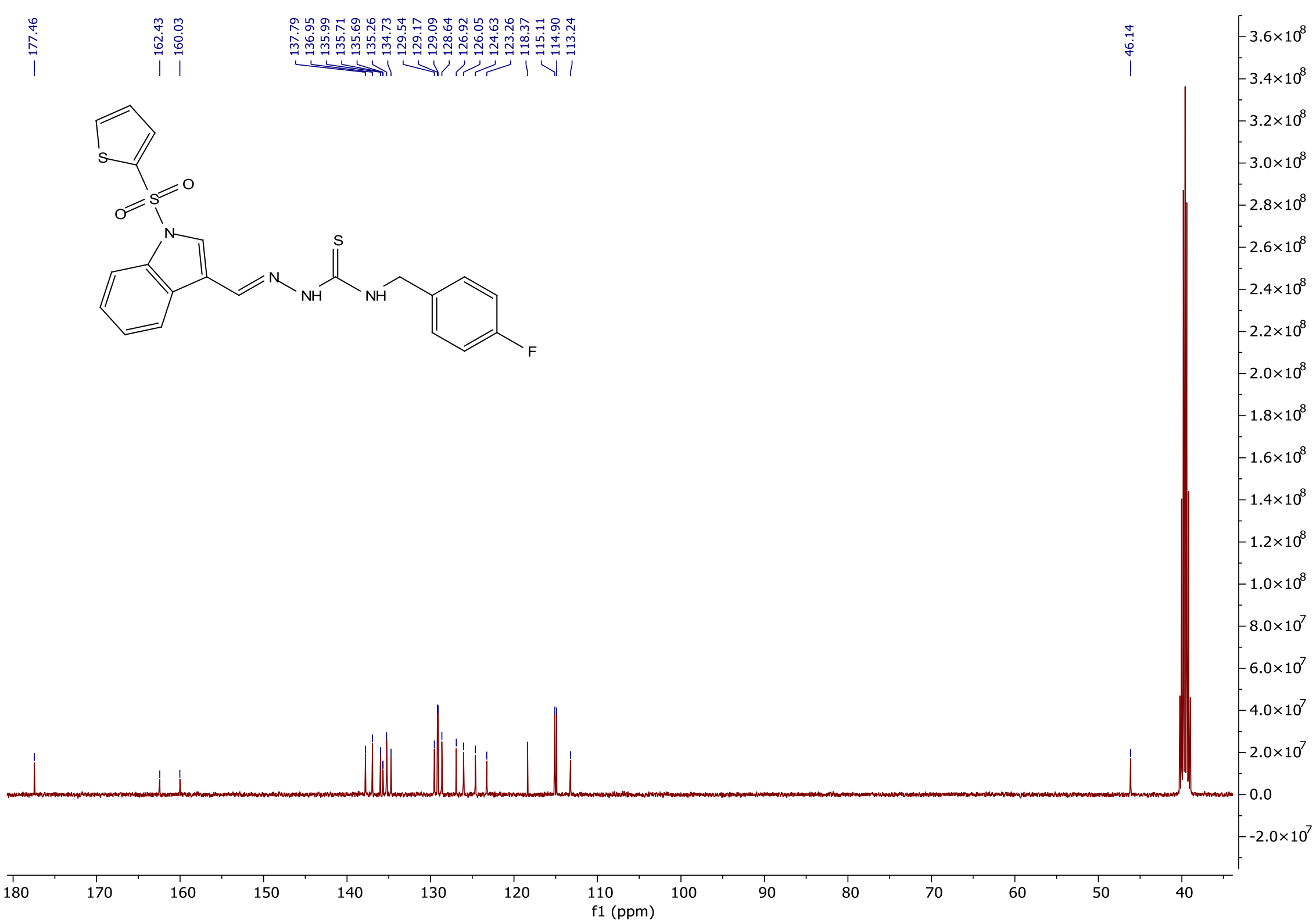

^{19}F NMR of 5q

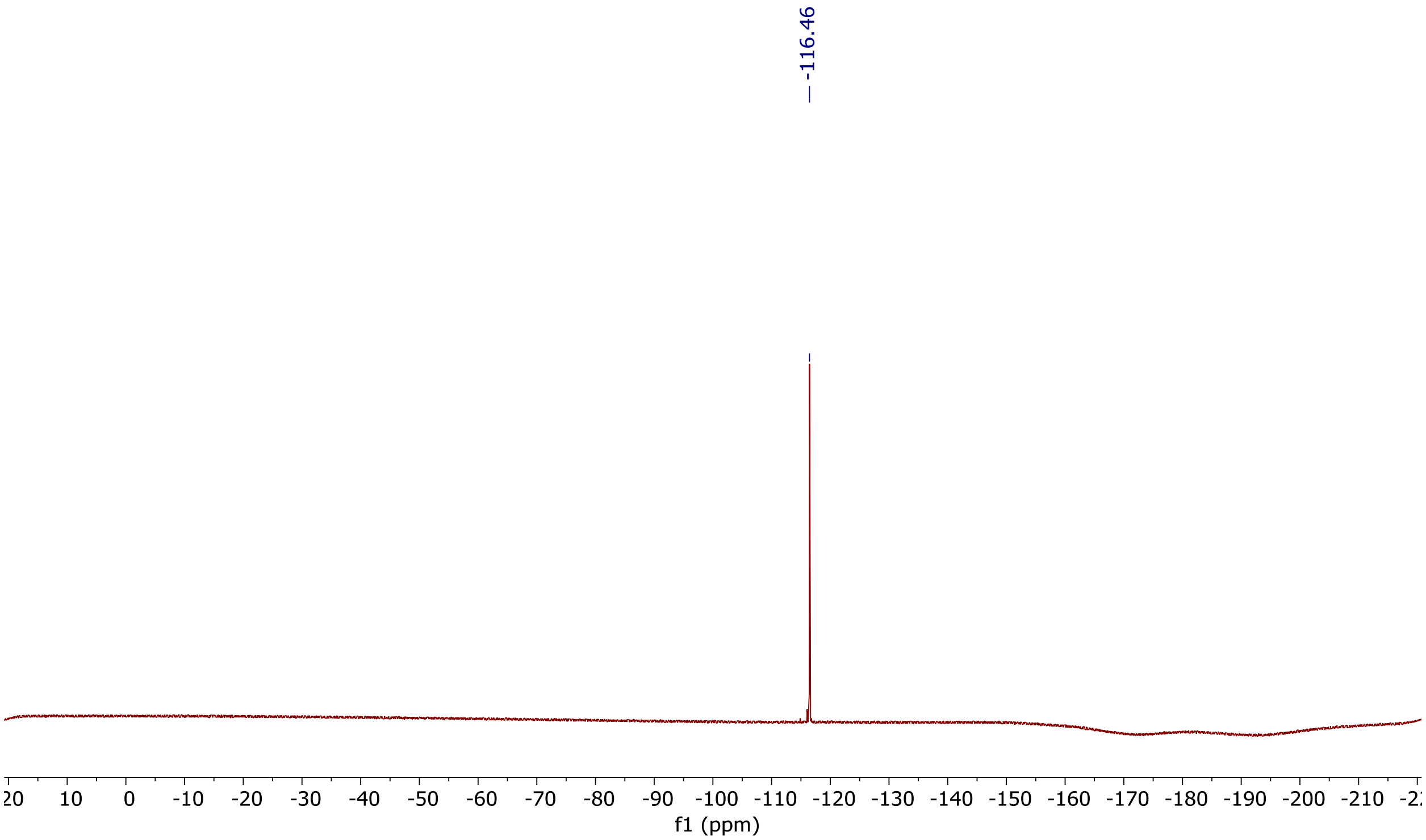

HRMS of 5q

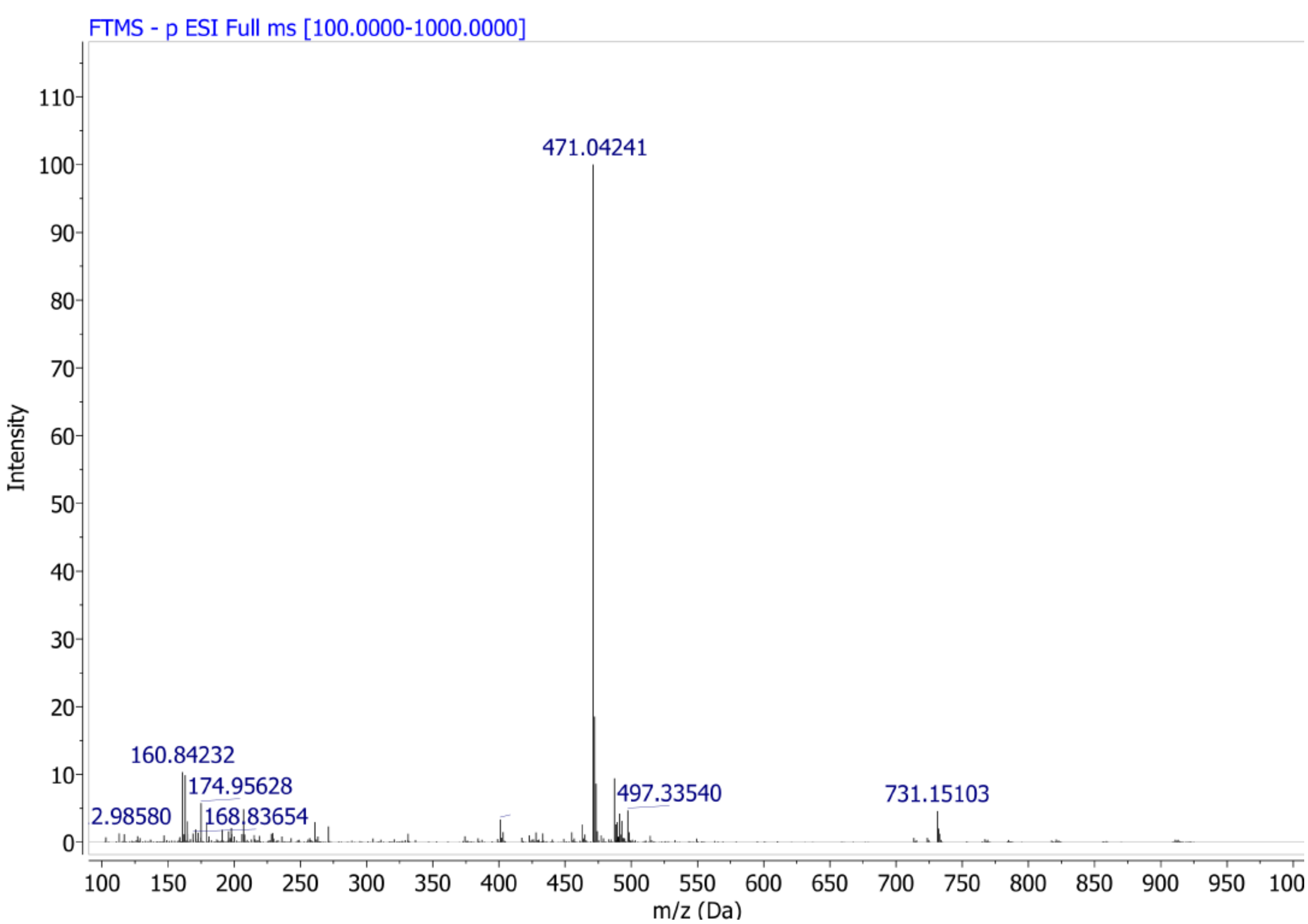

^{1}H, ^{19}F and ^{13}C NMR Spectra of 5r

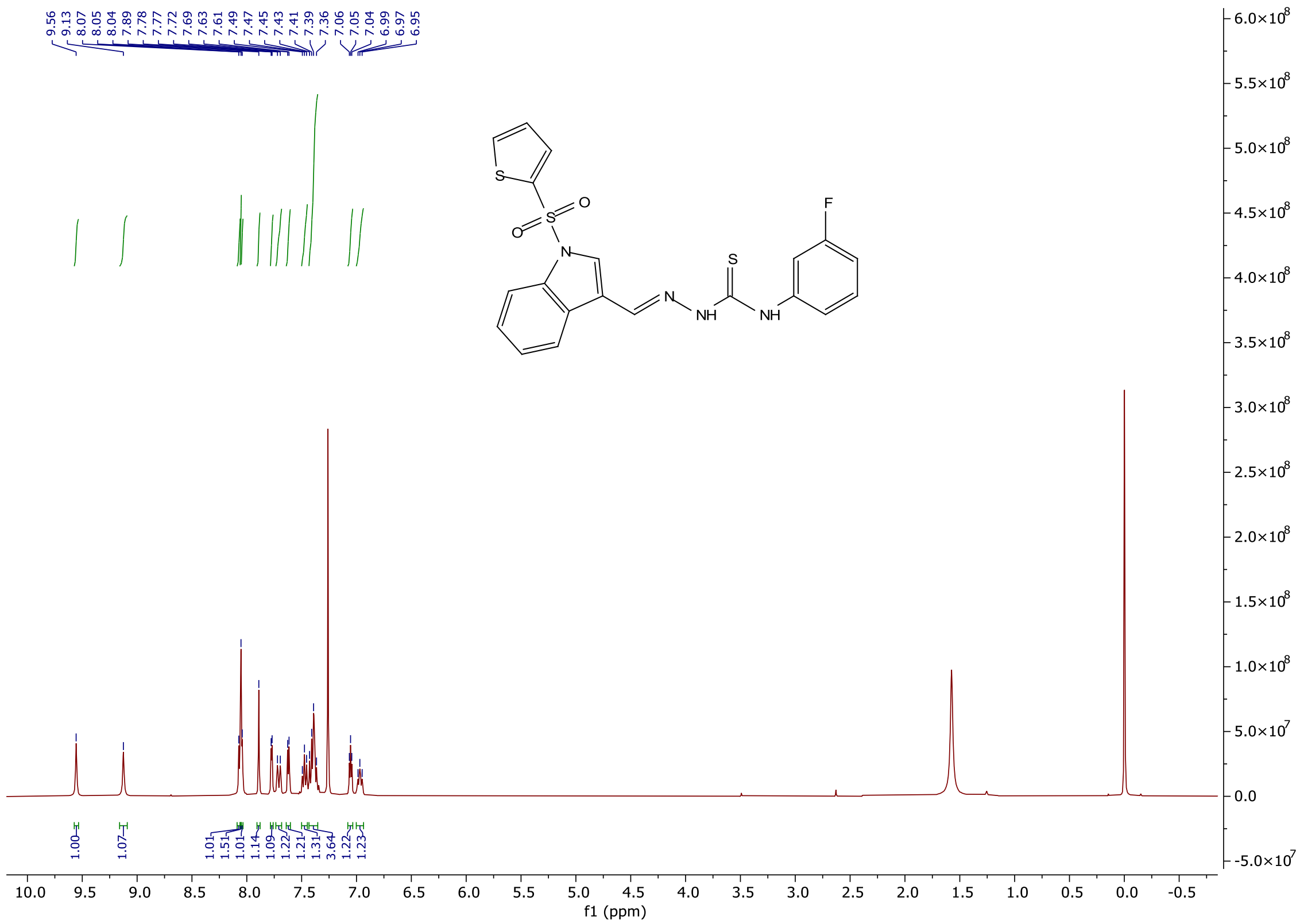

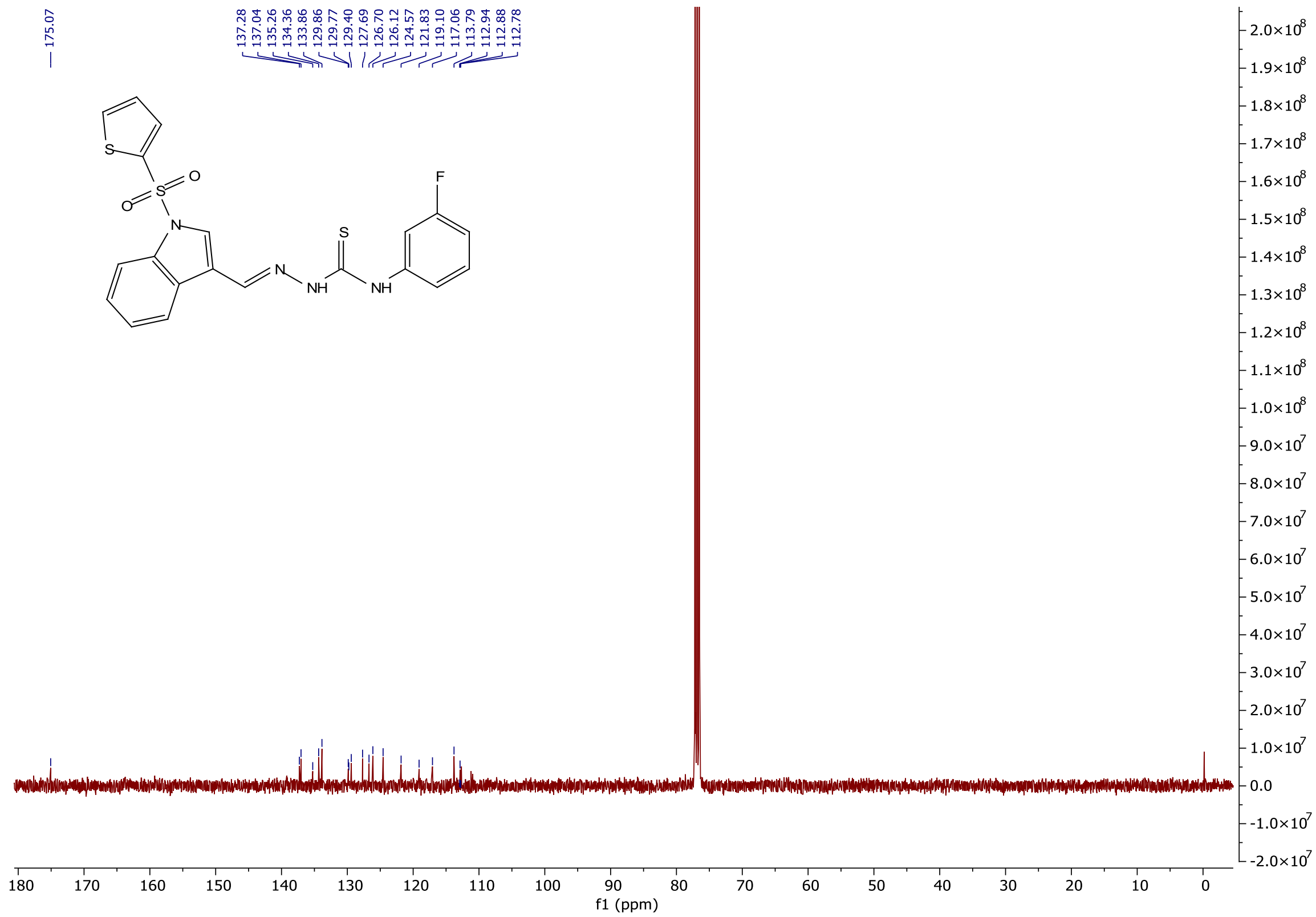

^{19}F NMR of 5r

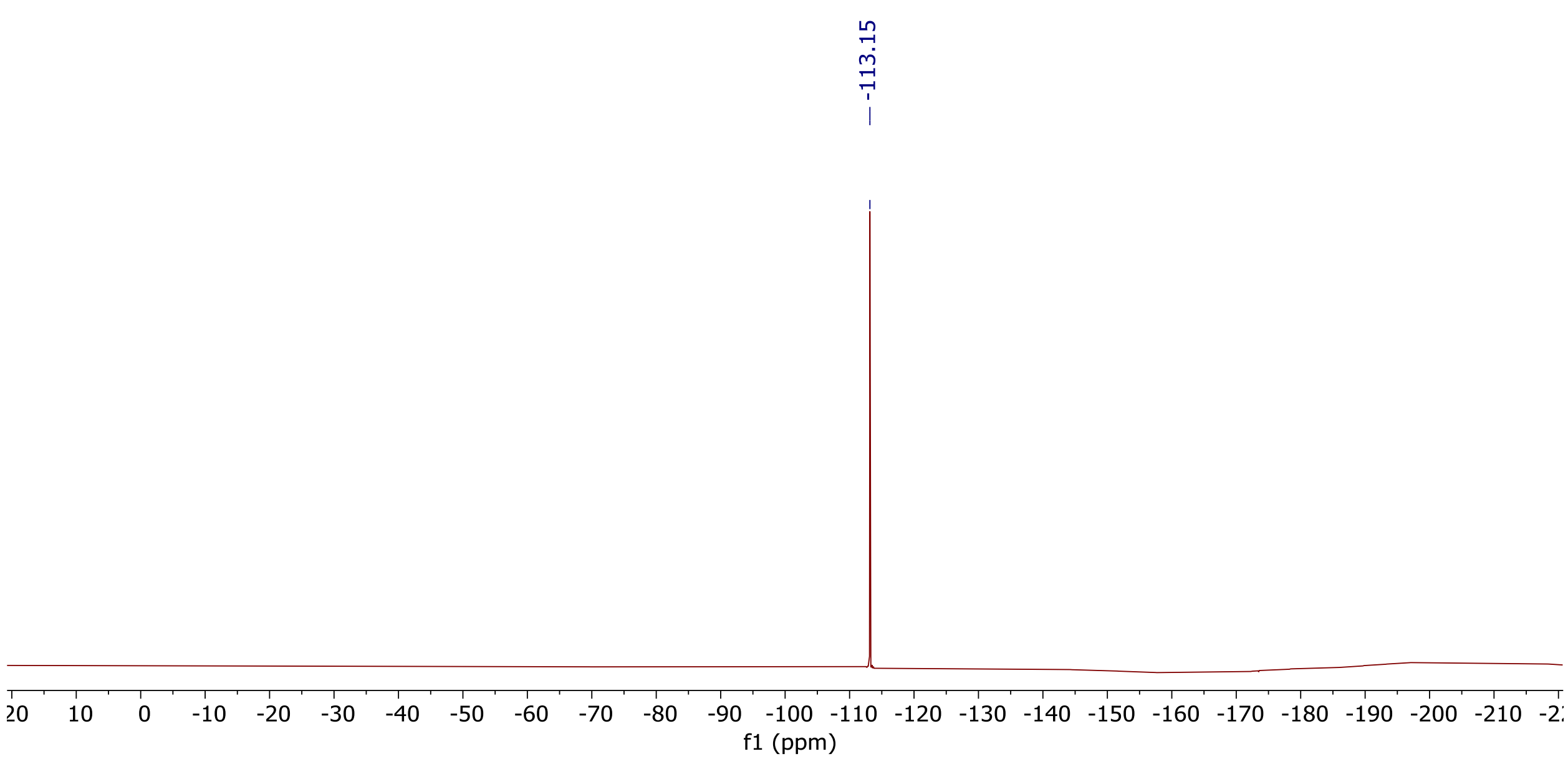

HRMS of 5r

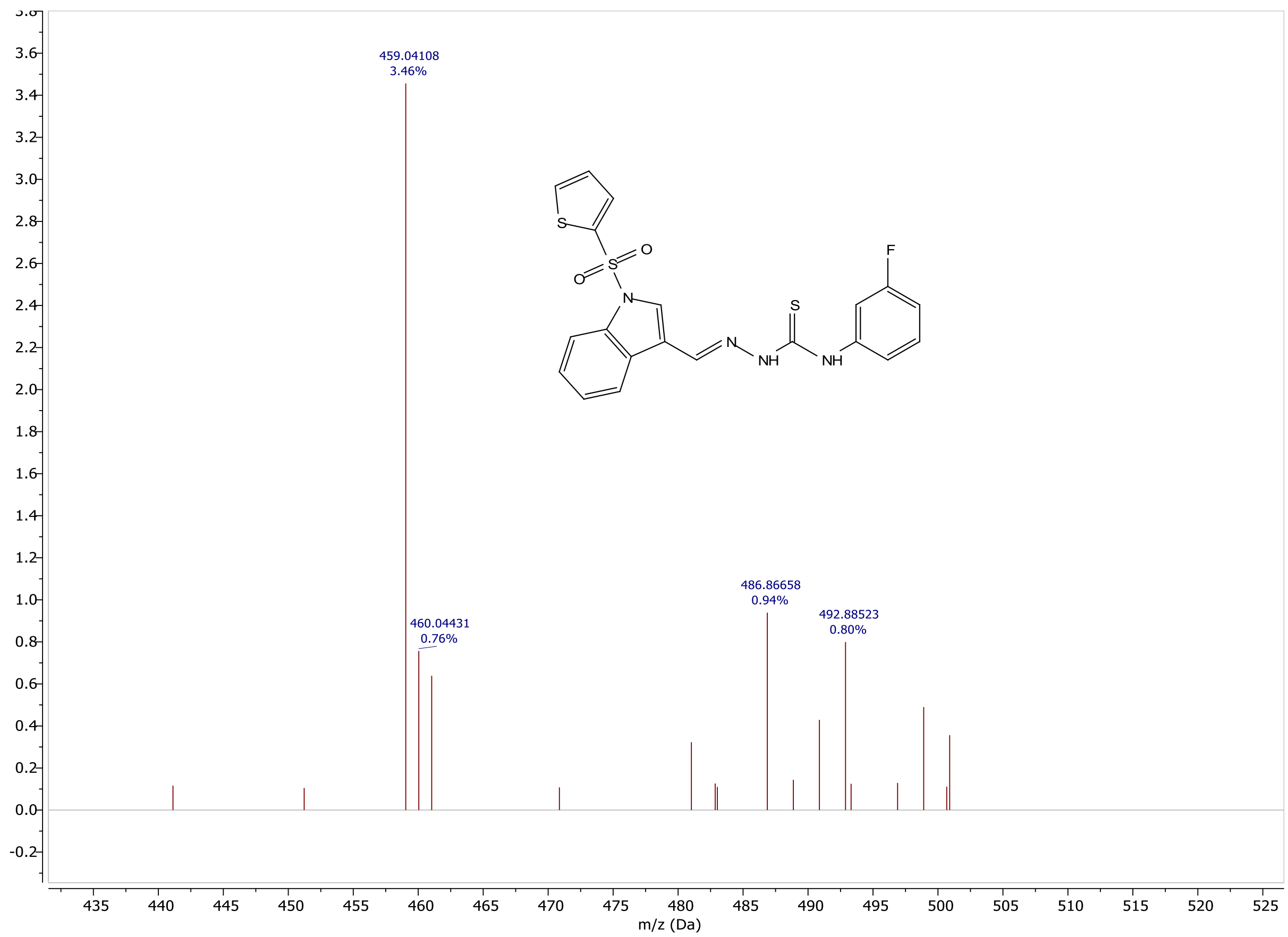

^{1}H & ^{13}C NMR Spectra of 5s

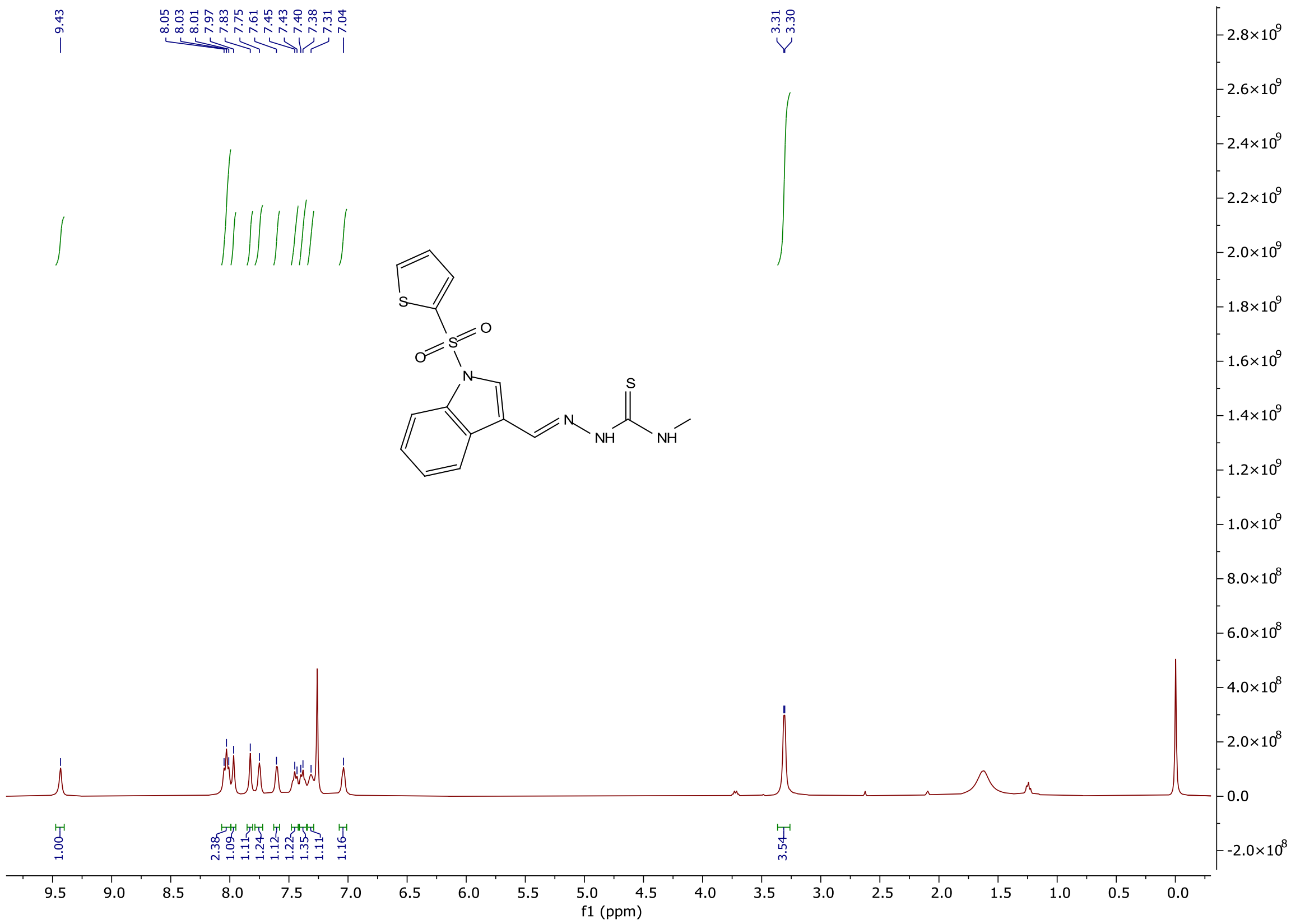
9.43
8.05
8.03
8.01
7.97
7.83
7.75
7.61
7.45
7.43
7.40
7.38
7.31
7.04
3.31
3.30
1.00
2.38
1.09
1.11
1.24
1.12
1.22
1.35
1.11
1.16
3.54
f1 (ppm)

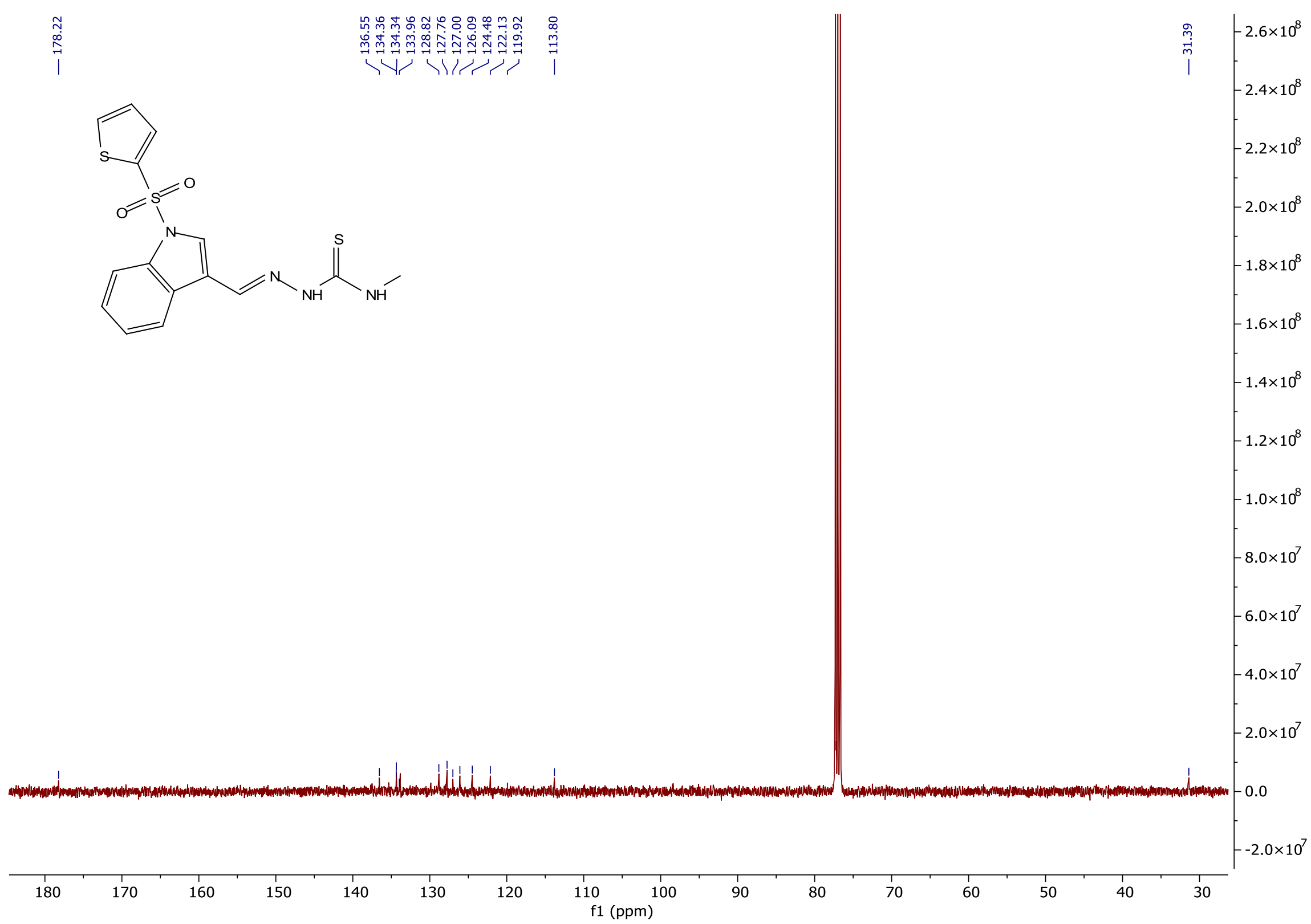
178.22
136.55
134.36
134.34
133.96
128.82
127.76
127.00
126.09
124.48
122.13
119.92
113.80
31.39
f1 (ppm)

HRMS of 5s

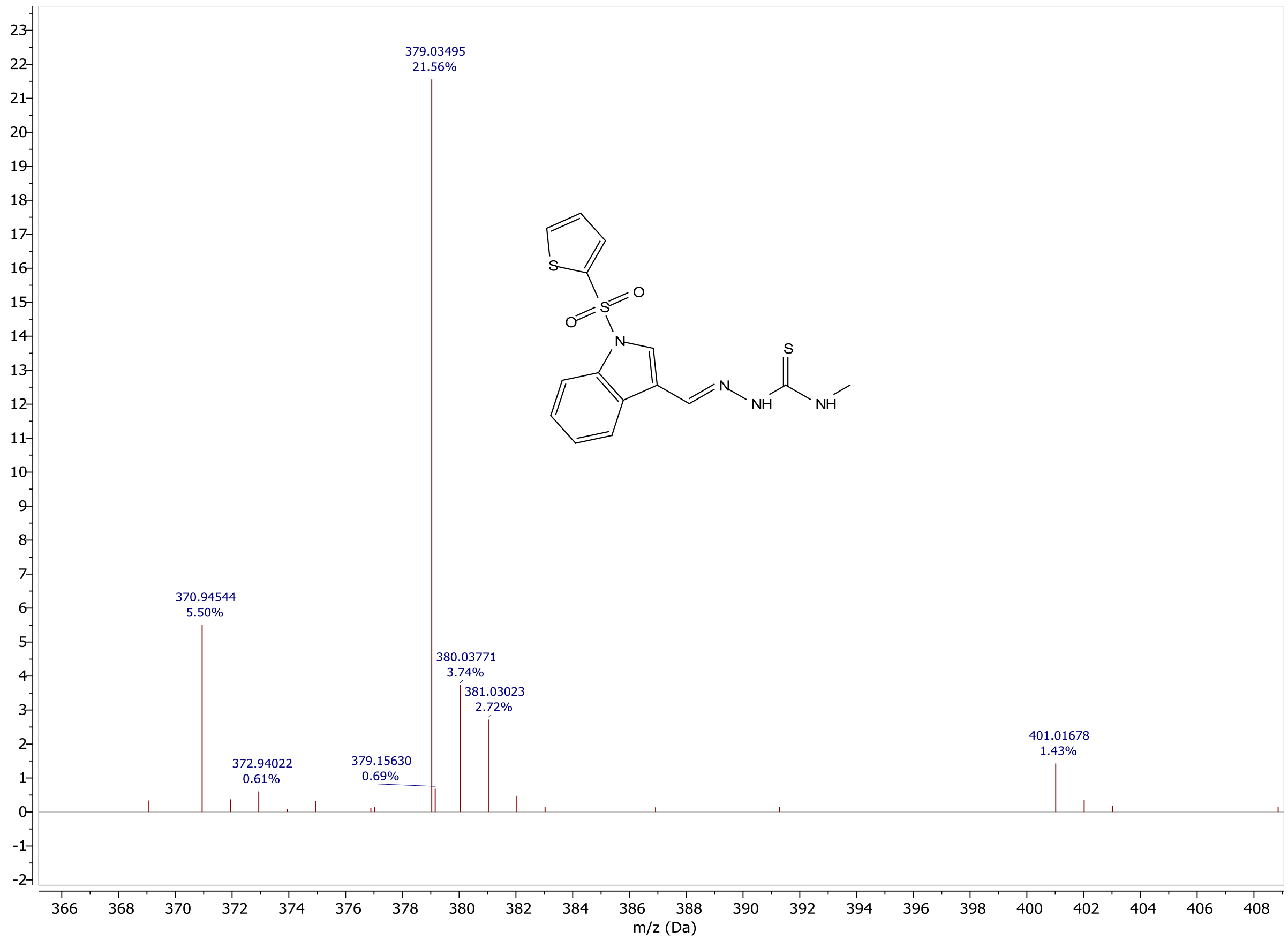
379.03495
21.56%
370.94544
5.50%
380.03771
3.74%
381.03023
2.72%
372.94022
0.61%
379.15630
0.69%
401.01678
1.43%
m/z (Da)

^{1}H & ^{13}C NMR Spectra of 5t

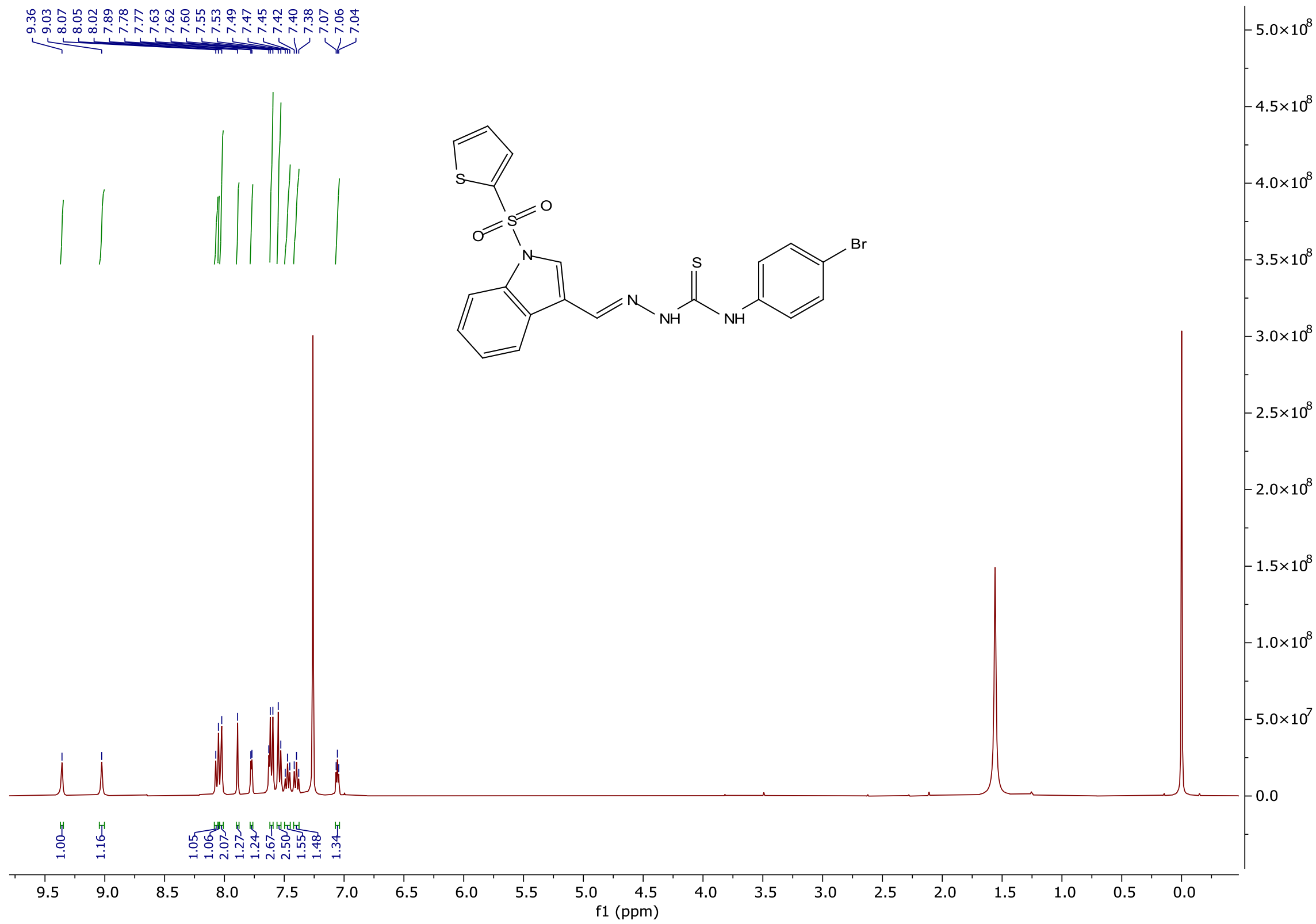

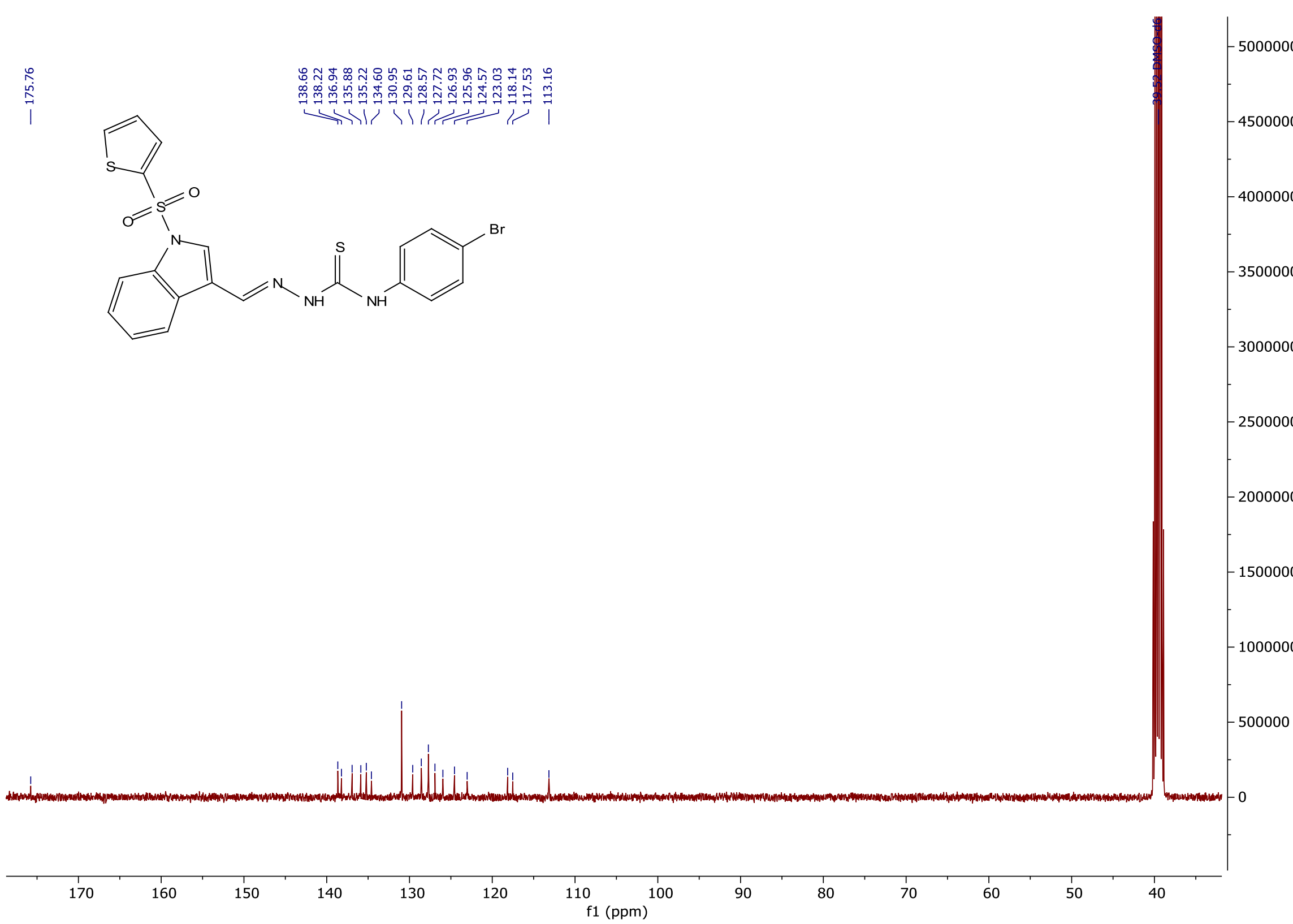

HRMS of 5t

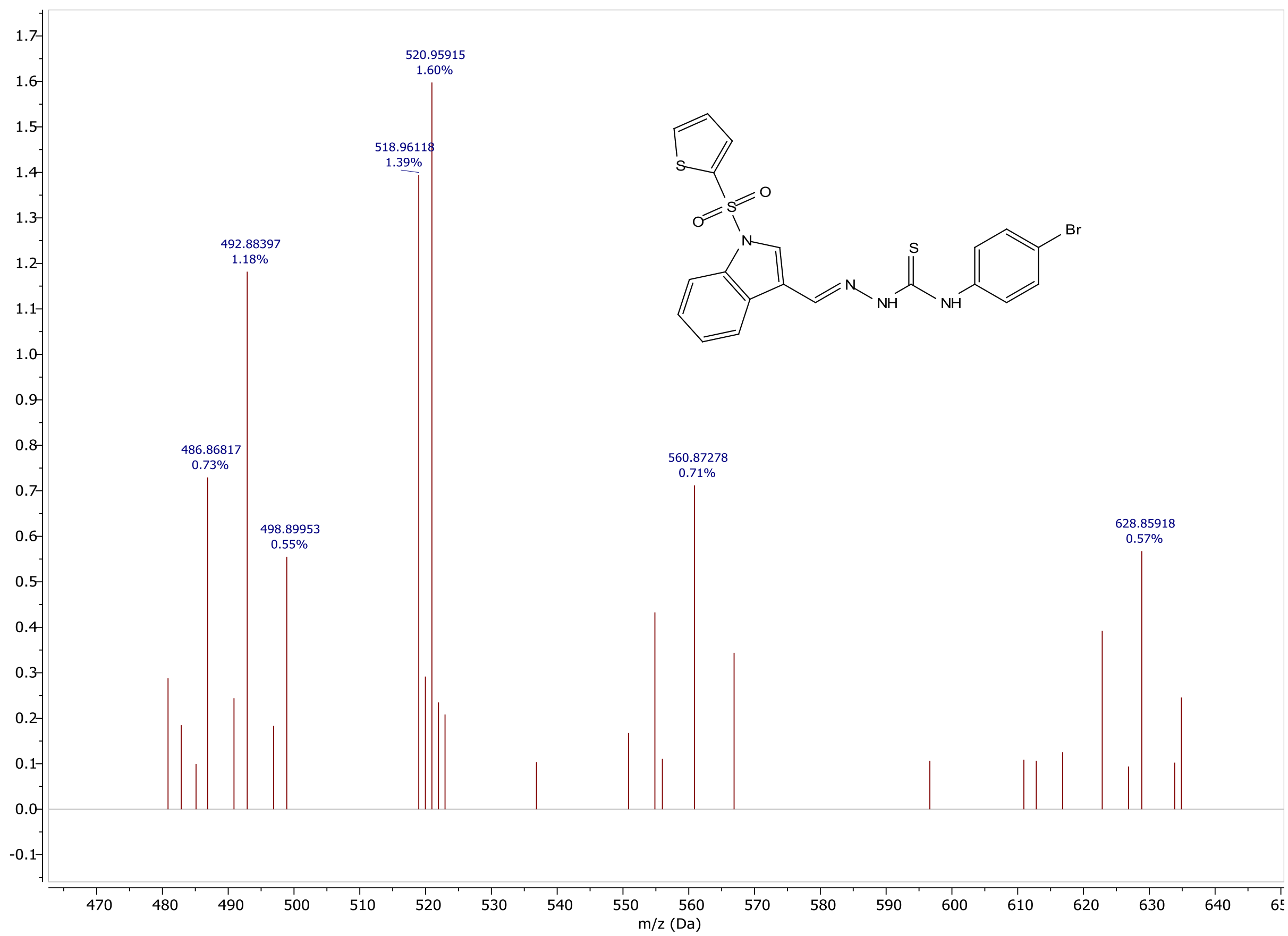

520.95915
1.60%
518.96118
1.39%
492.88397
1.18%
486.86817
0.73%
498.89953
0.55%
560.87278
0.71%
628.85918
0.57%
m/z (Da)

IC_{50} Graphics of 5a

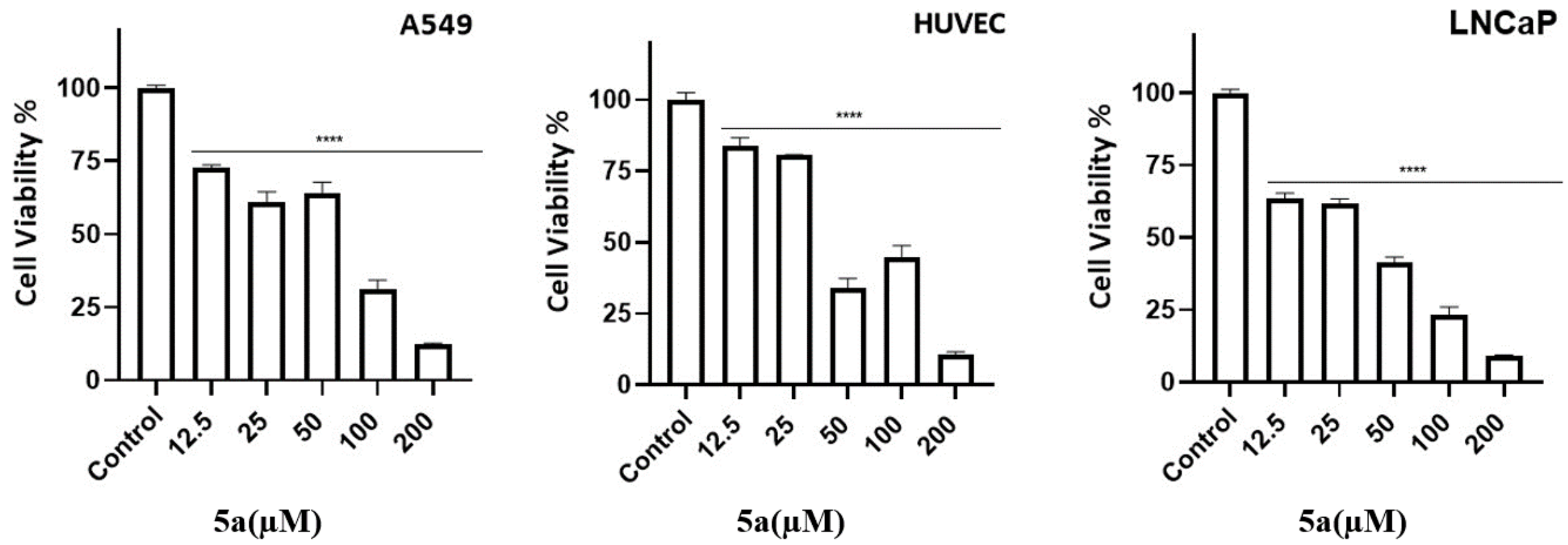

IC_{50} Graphics of 5b

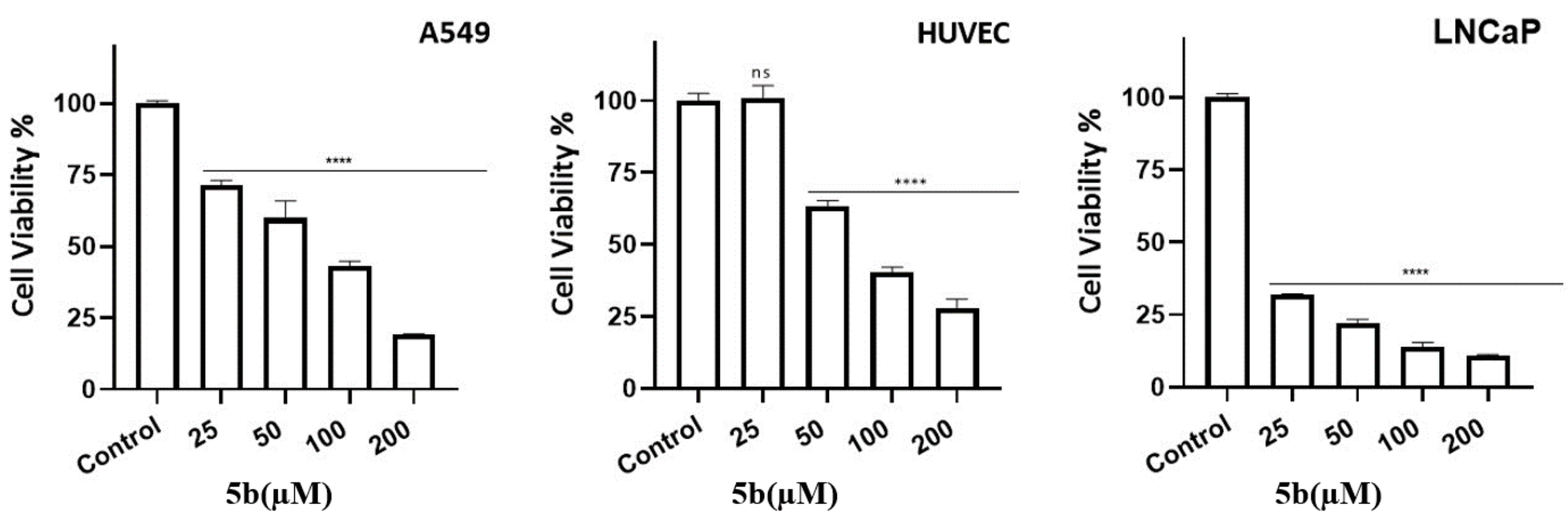

IC_{50} Graphics of 5c

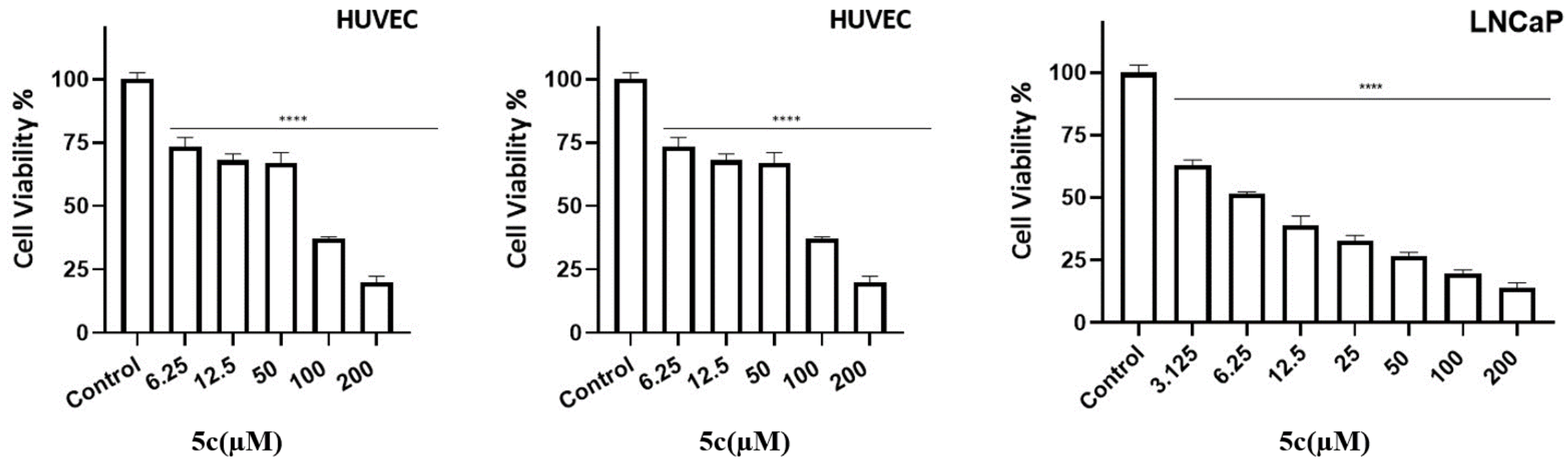

IC_{50} Graphics of 5d

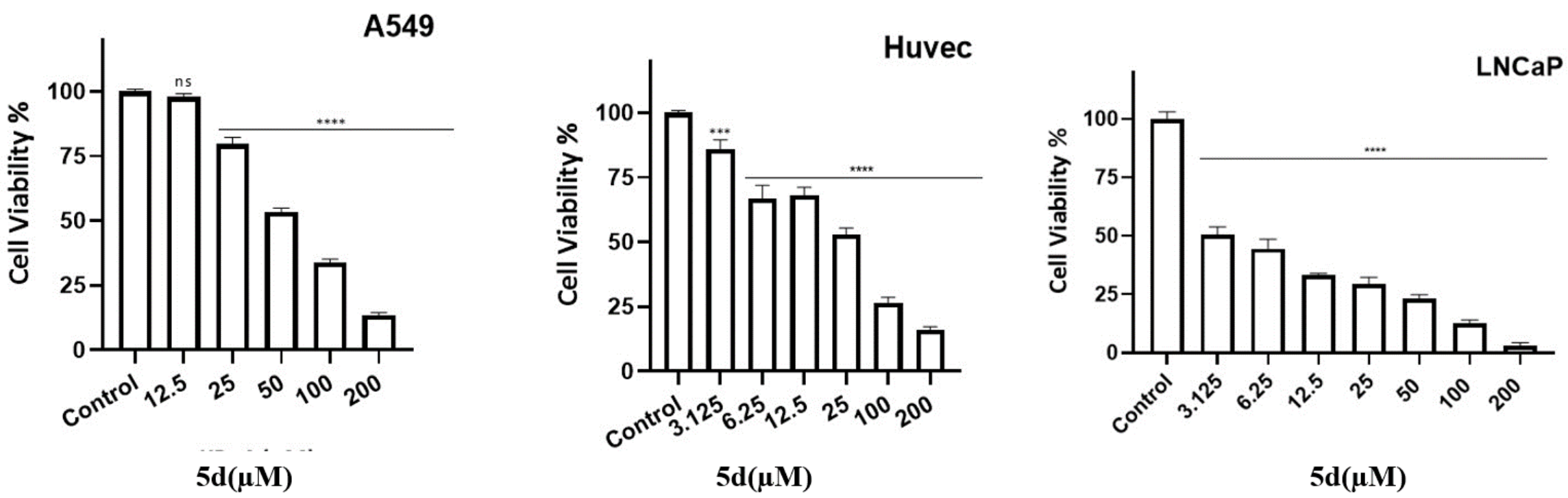

IC_{50} Graphics of 5e

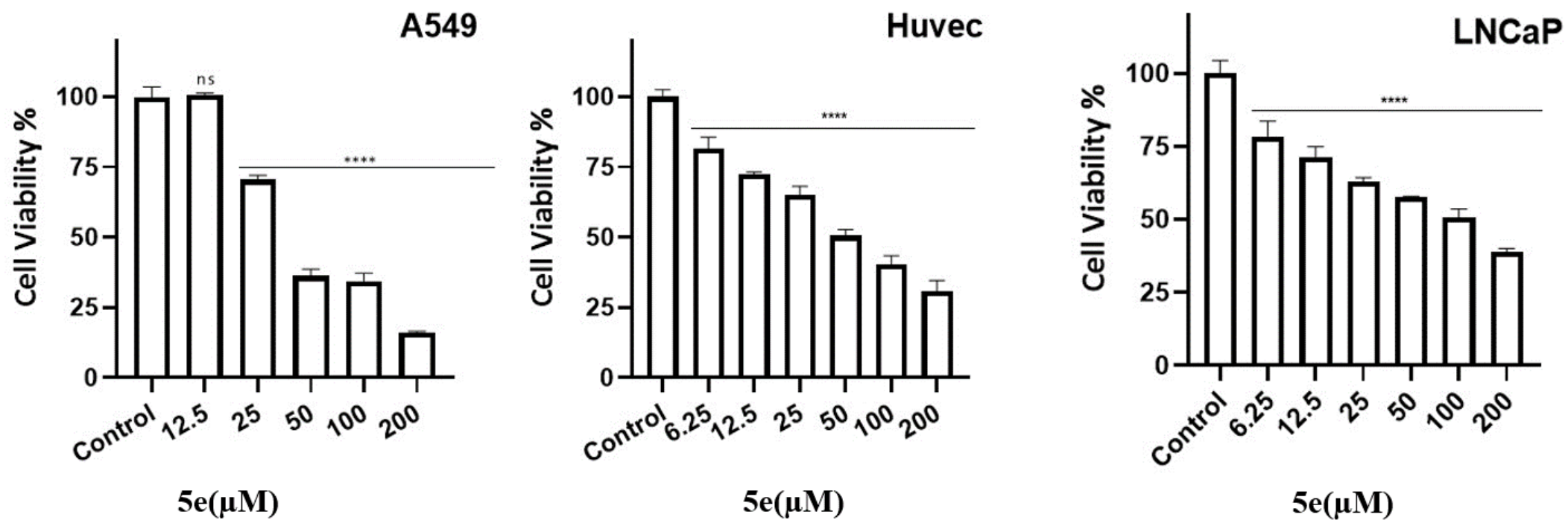

IC_{50} Graphics of 5f

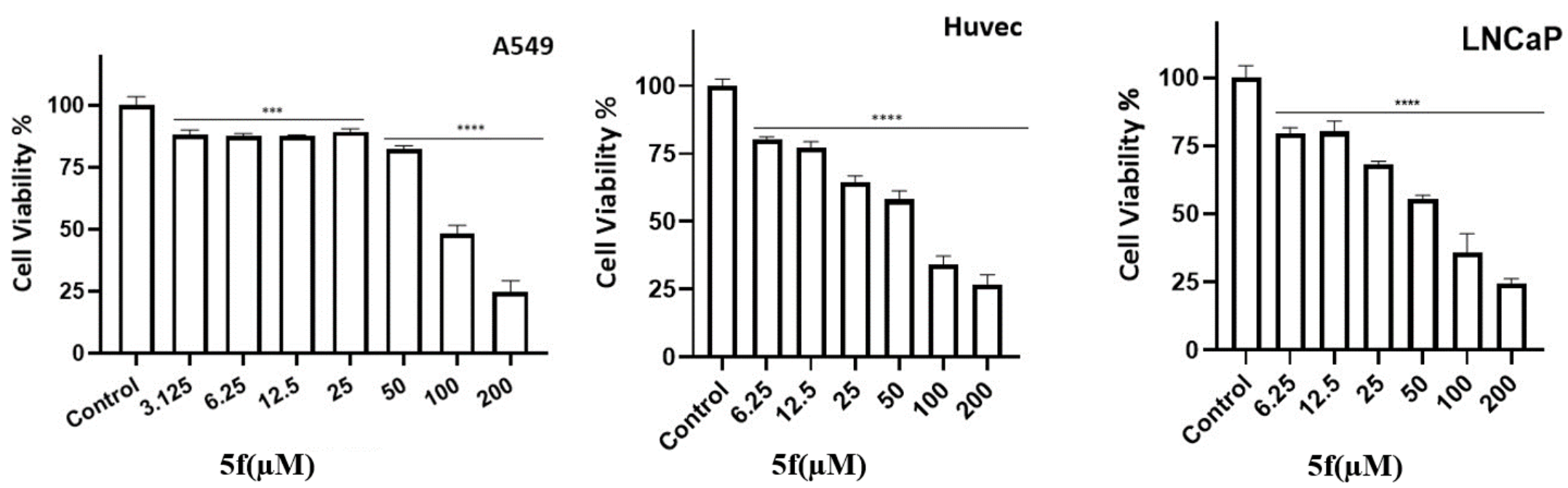

IC$_{50}$ Graphics of 5g

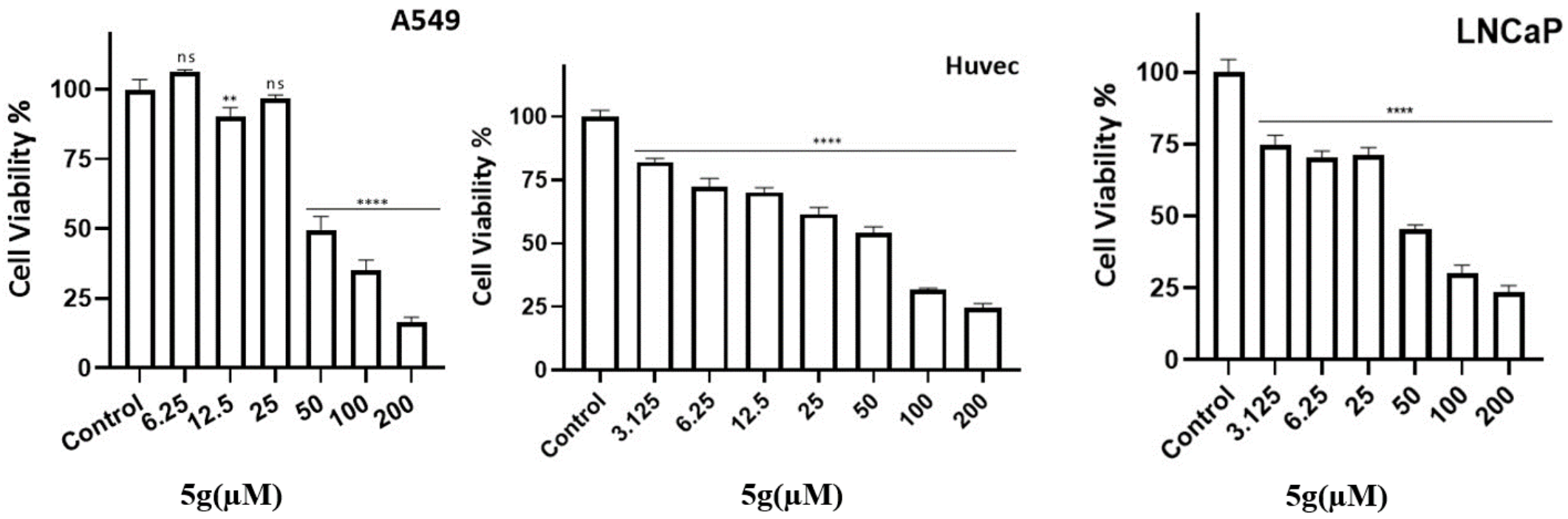

IC$_{50}$ Graphics of 5h

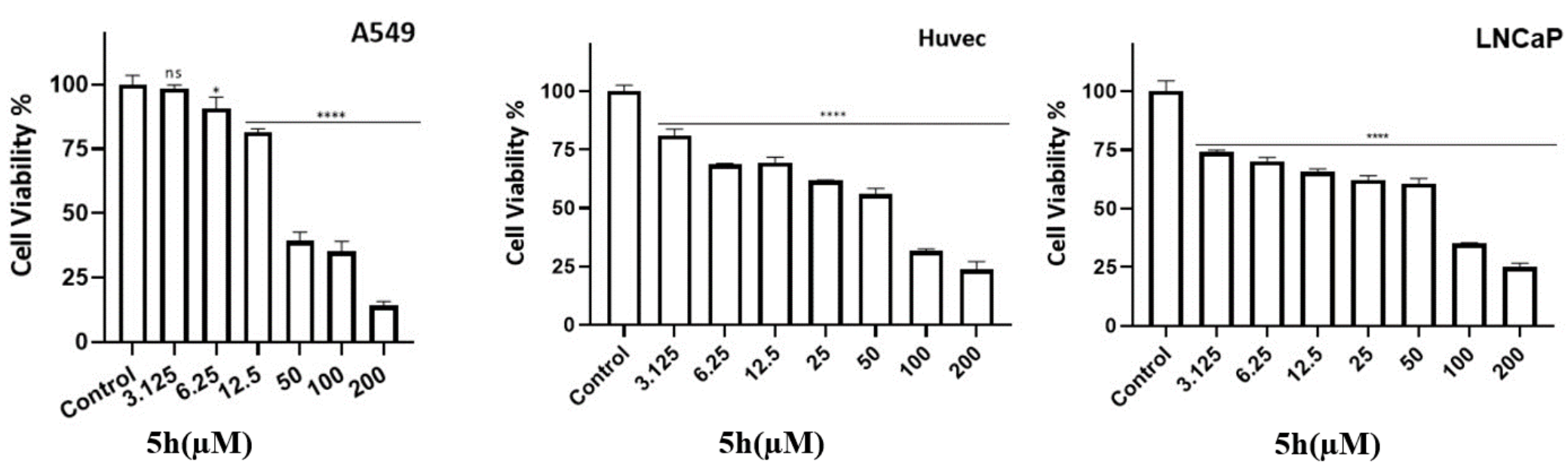

IC_{50} Graphics of 5i

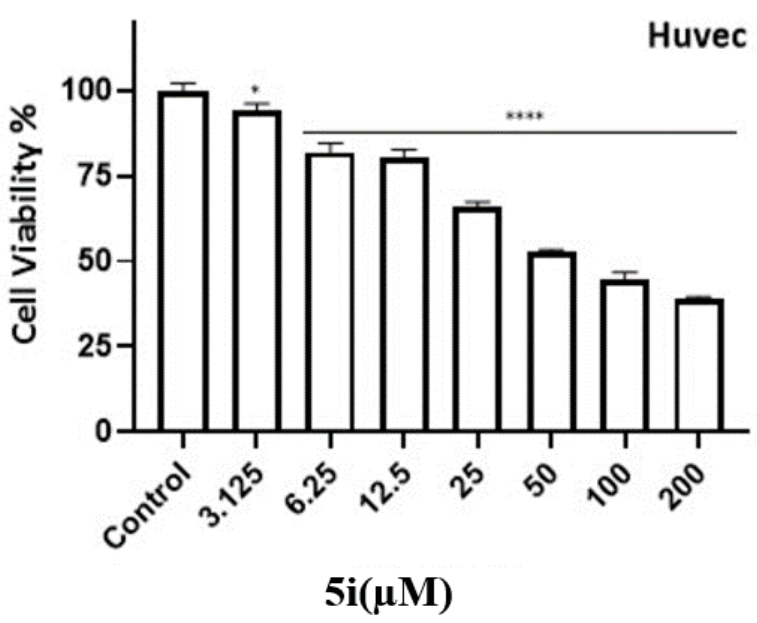

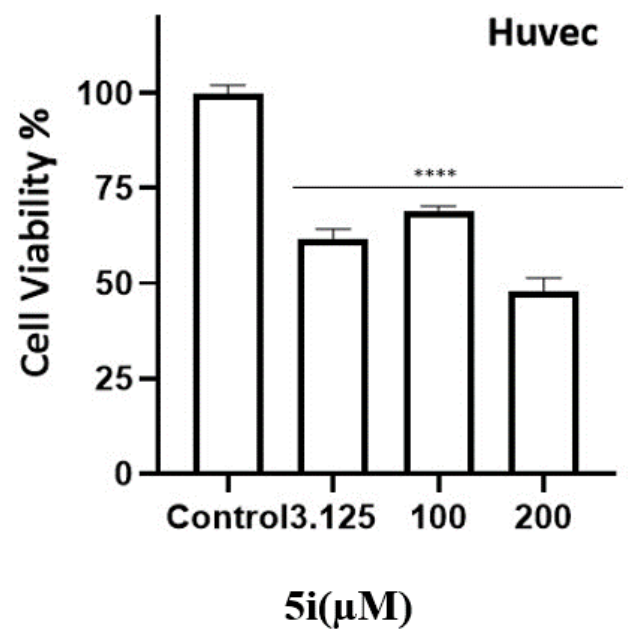

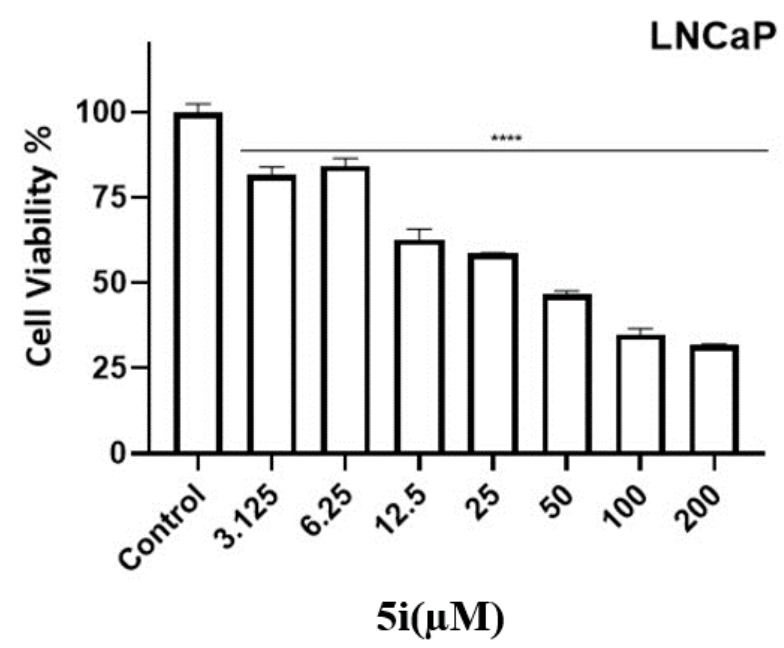

IC_{50} Graphics of 5j

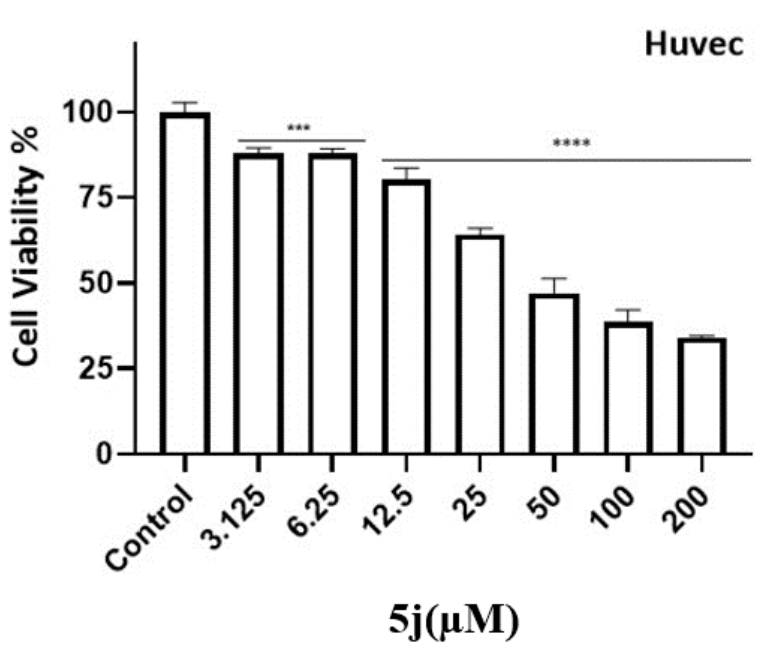

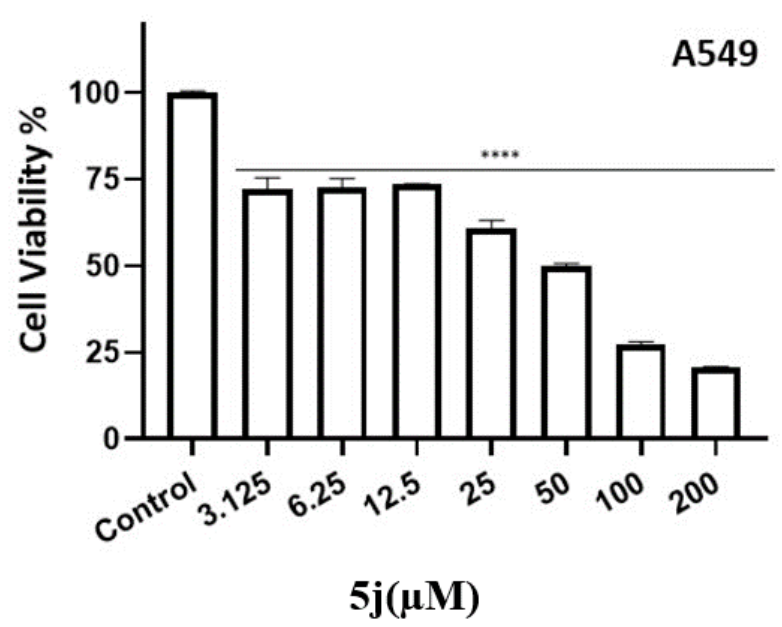

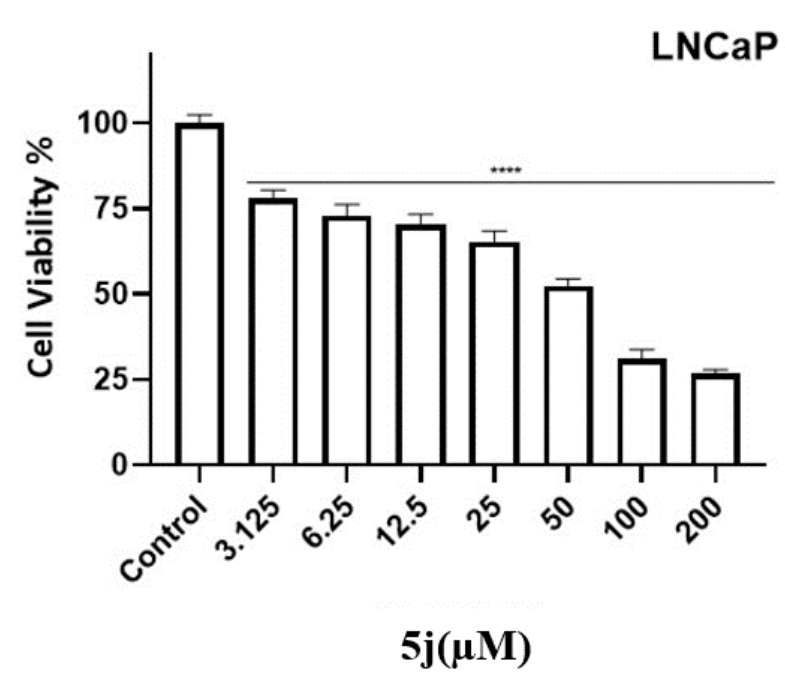

IC_{50} Graphics of 5k

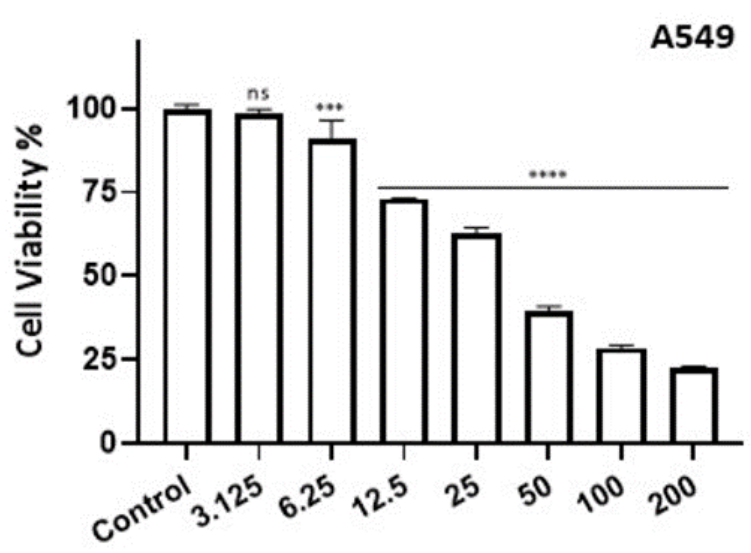

5k(µM)

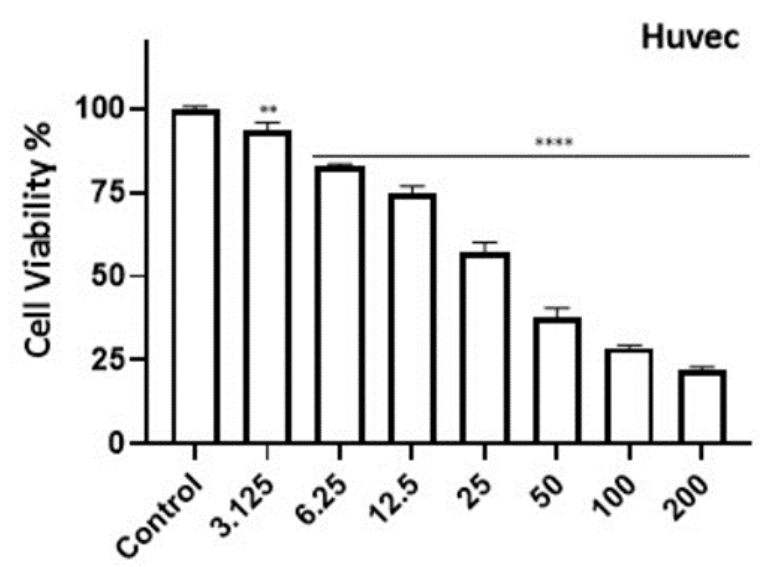

5k(µM)

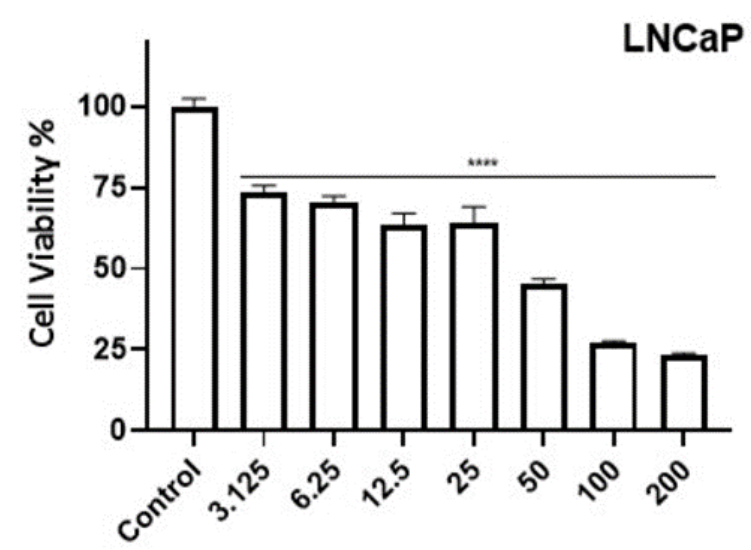

5k(µM)

IC_{50} Graphics of 5l

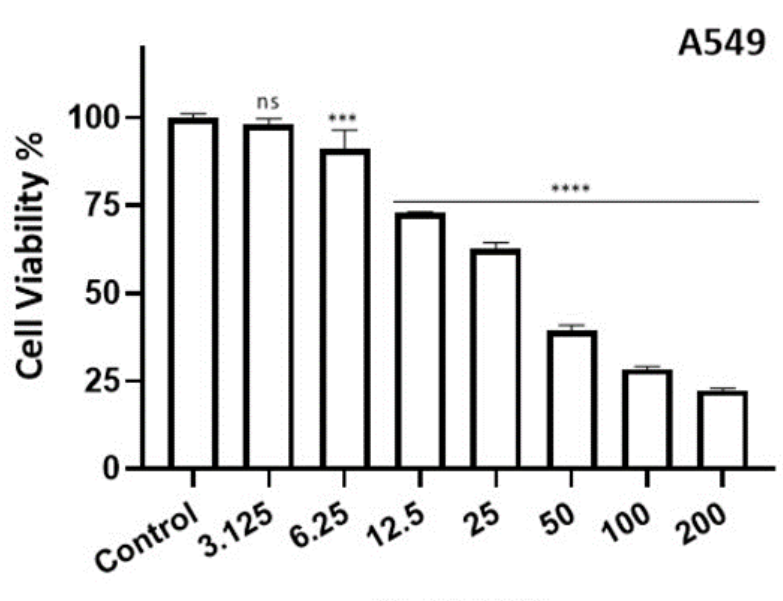

5l(µM)

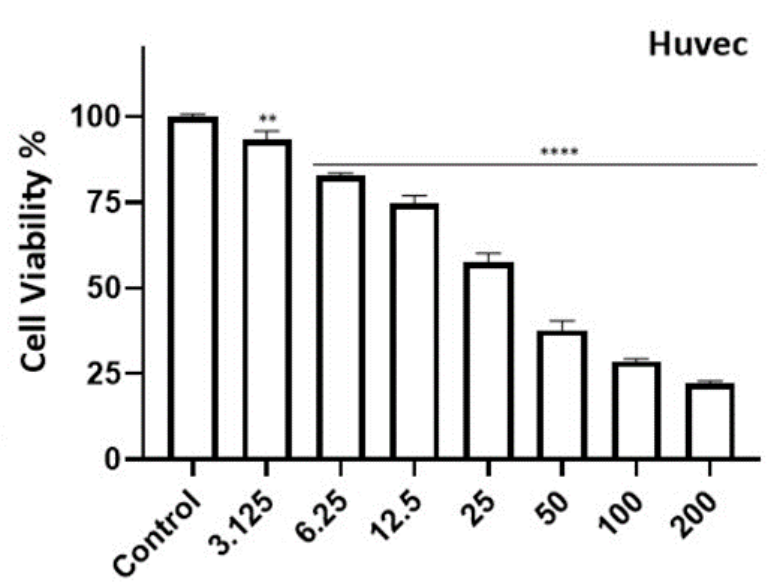

5l(µM)

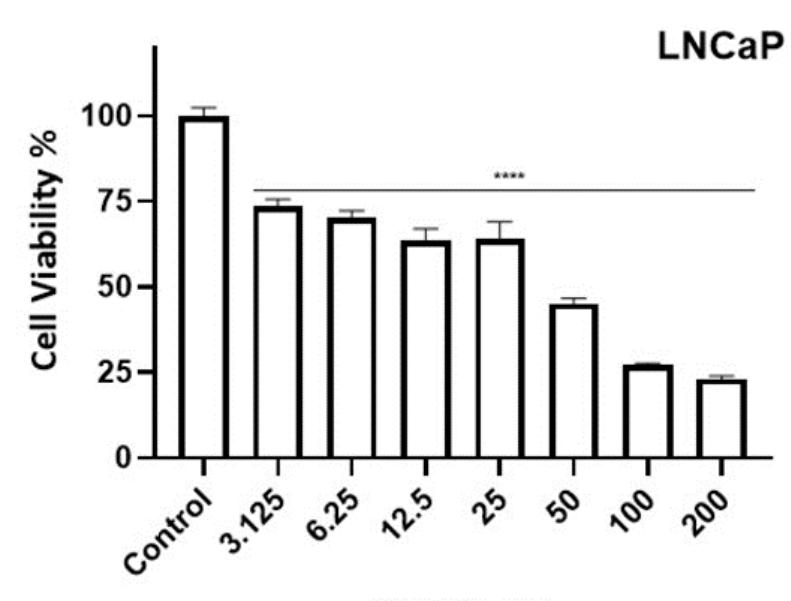

5l(µM)

IC50 Graphics of 5m

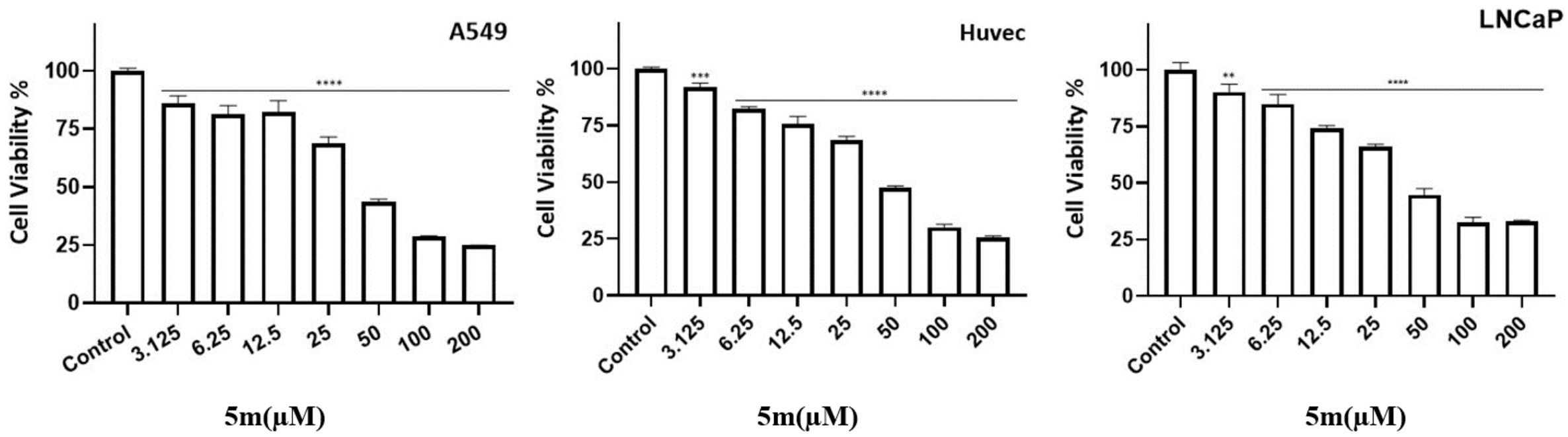

IC50 Graphics of 5n

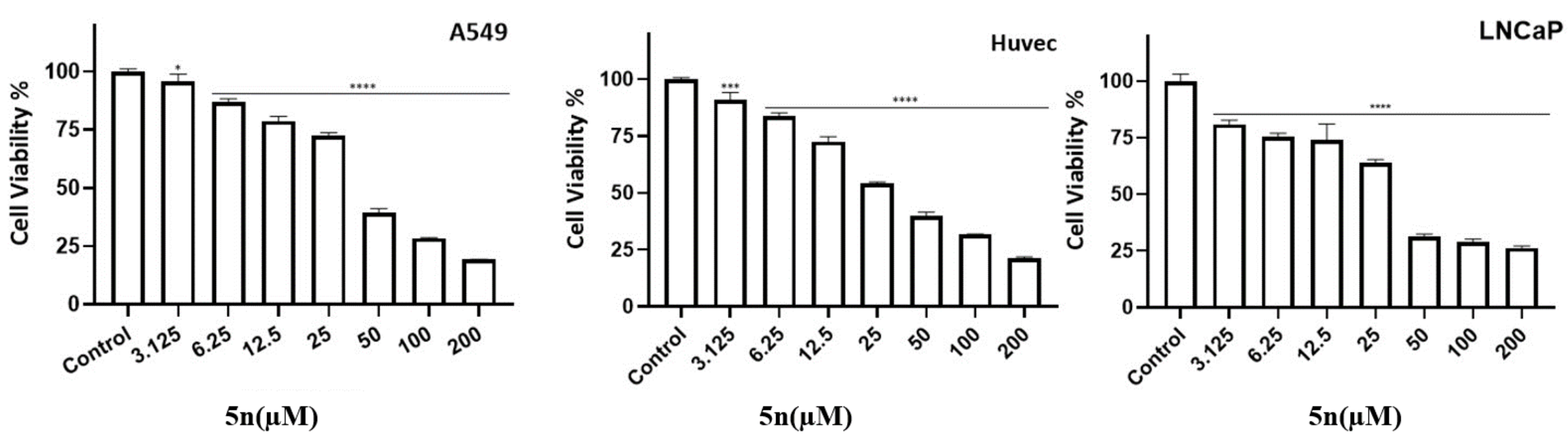

IC_{50} Graphics of 5o

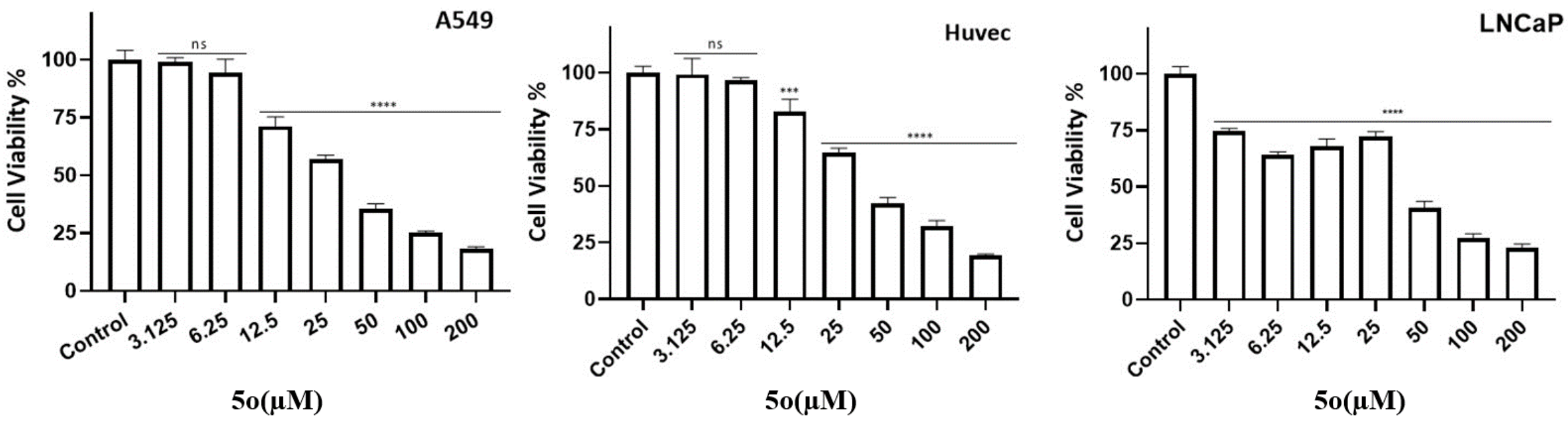

IC_{50} Graphics of 5p

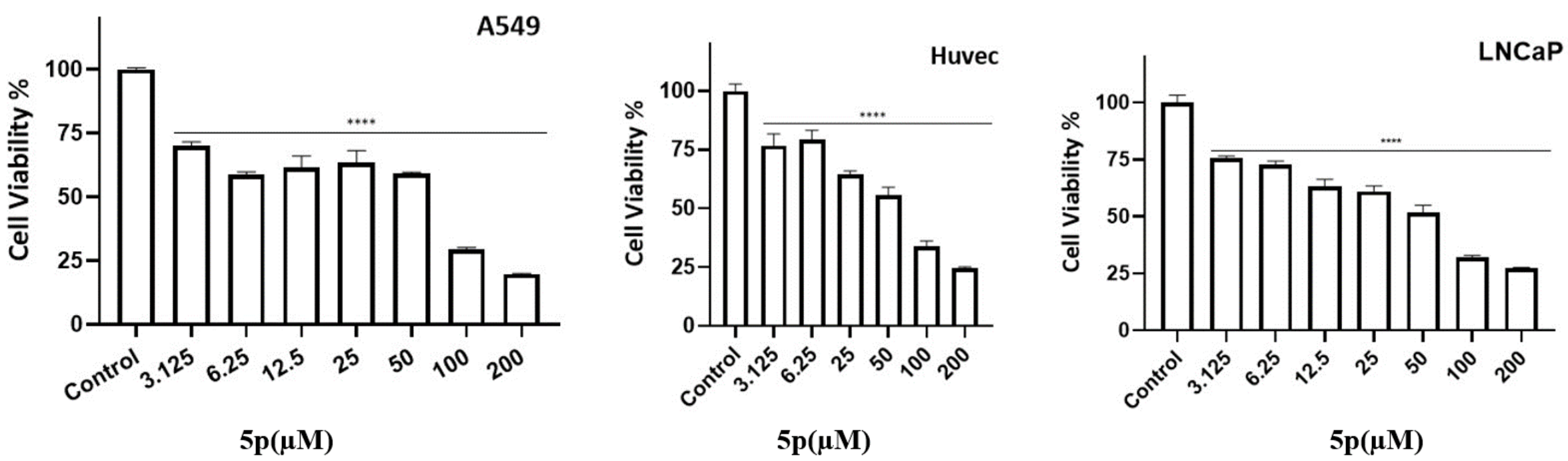

IC50 Graphics of 5q

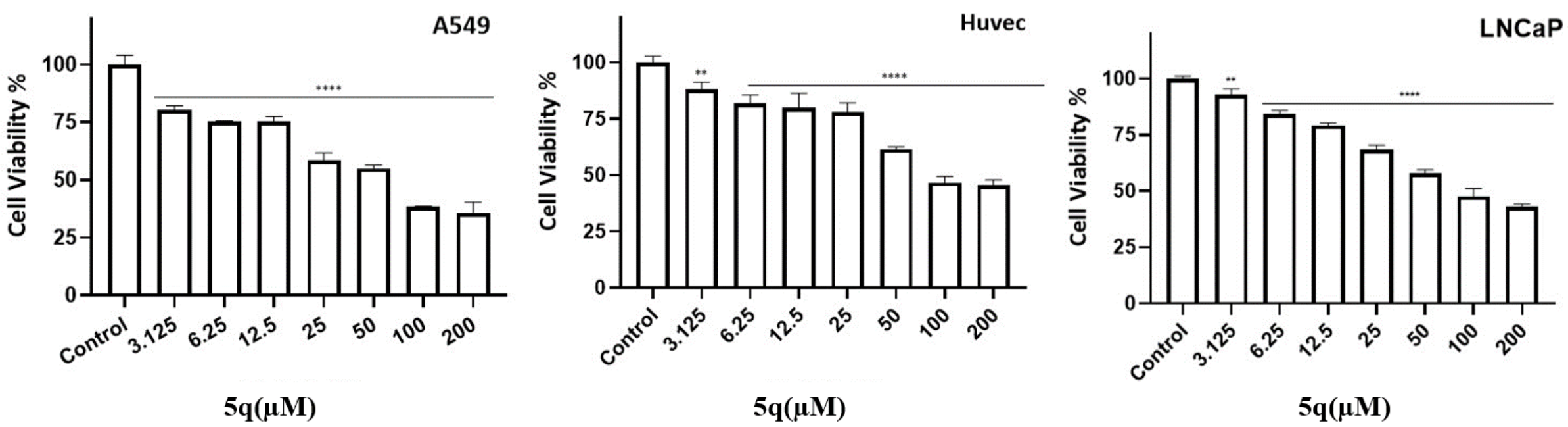

IC50 Graphics of 5r

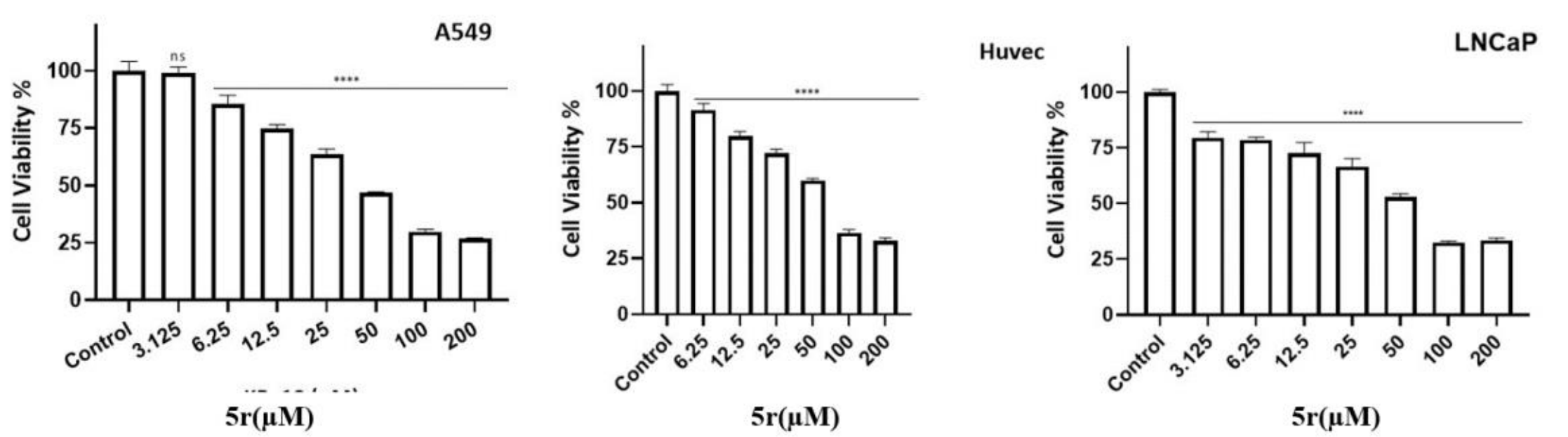

IC$_{50}$ Graphics of 5s

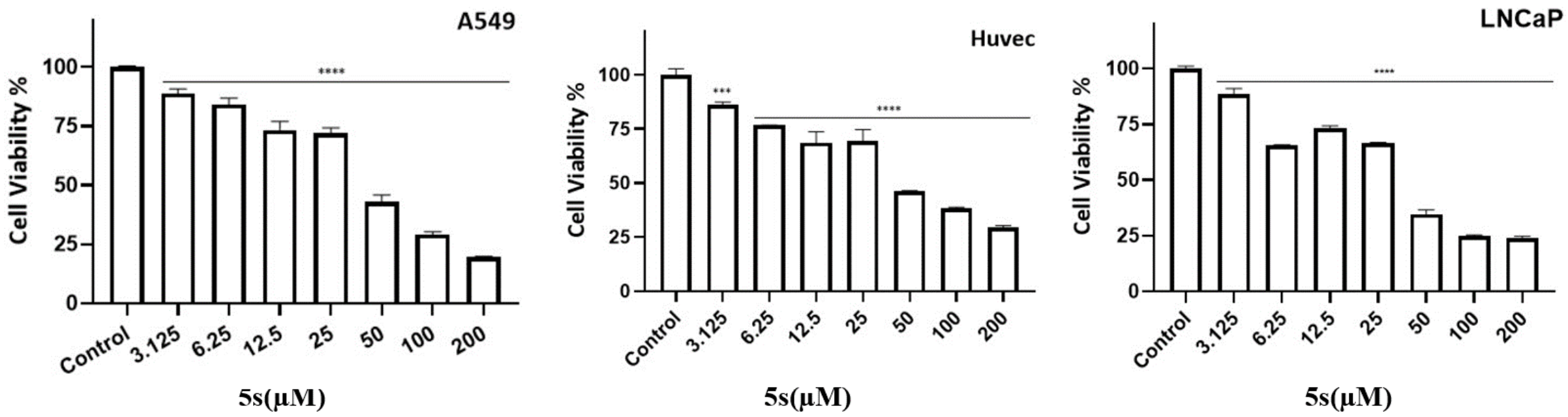

IC$_{50}$ Graphics of 5t

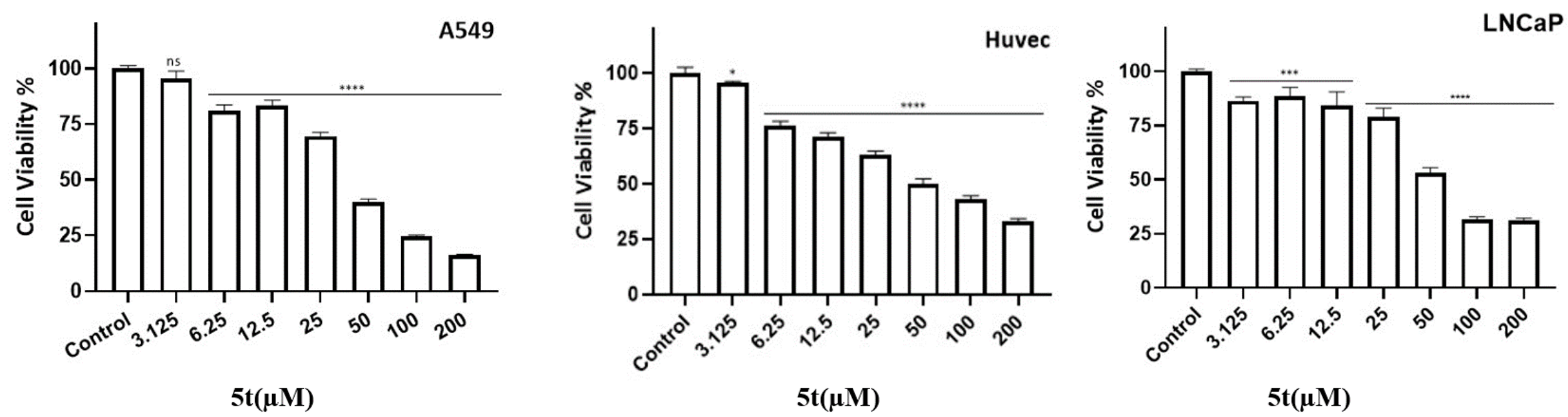

Supplement: Supplementary file 1 [file RA-OLF-D6RA08602K-s001.pdf]
